# Bis(*tert*-butoxydiphenylsilyl)amide Divalent Lanthanide Complexes

**DOI:** 10.1021/acs.inorgchem.5c00277

**Published:** 2025-05-23

**Authors:** Grant R. Wilkinson, Sarah J. Schultz, Kaitlyn S. Otte, Maximilian G. Bernbeck, Henry S. La Pierre

**Affiliations:** † School of Chemistry and Biochemistry, 1372 Georgia Institute of Technology, Atlanta, Georgia 30332-0400, United States; ‡ Nuclear and Radiological Engineering and Medical Physics Program, Georgia Institute of Technology, Atlanta Georgia 30332-0400, United States

## Abstract

The development of new ligand systems to stabilize “non-traditional/non-classical” divalent lanthanides is key to tuning the chemical and physical properties of their mixed principal quantum number 4f^n^5d^1^ ground states. The design and study of novel ligand systems which stabilize occupation of differing orbitals within the 5d manifold for these ions constitutes an area ripe for exploration. Our efforts toward the development of redox-innocent bulky silylamide ligands to stabilize pseudo-octahedral coordination geometries for divalent lanthanides have resulted in the synthesis of the bis­(*tert*-butoxydiphenylsilyl)­amide ligand, whose coordination complexes with Sm^2+^, Eu^2+^, and Yb^2+^ are reported herein. These systems have been fully characterized by single-crystal X-ray diffraction, elemental analysis, cyclic voltammetry, direct-current magnetometry, and infrared, nuclear magnetic resonance, and electronic absorption spectroscopies. Attempts to extend this system to the more reducing Tm^2+^ ion resulted in an inseparable mixture of products from which crystals of the analogous Tm^2+^ species and a reduced dinitrogen, bimetallic Tm^3+^-Tm^3+^ complex bridged by a η^2^-N_2_^3–^ radical could be identified. Though progress toward six-coordinate complexes of reducing “traditional/classical” divalent ions is noted for these systems, further work is needed to improve the synthetic utility of this ligand framework for the study of “non-traditional/non-classical” divalent lanthanides with a mixed-principal quantum number 4f^n^5d^1^ ground state.

## Graphical abstract

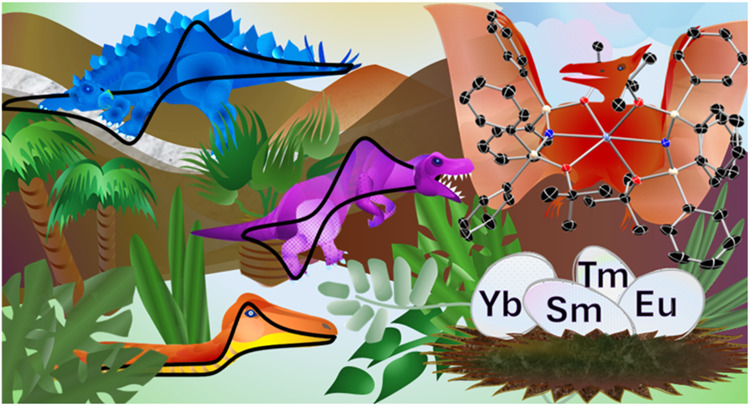

## Introduction

Cyclopentadienyl ligands (Cp) and their derivatives have facilitated the isolation of molecular divalent lanthanides with mixed-principal quantum number ground states, e.g., 4f^n^5d^1^, beginning in 2008.^1^ These ions have been described as “non-traditional/non-classical” ^2–5^ in contrast to long-established divalent ions of Sm, Eu, Tm,^6^ and Yb (and sometimes Nd^7–10^ and Dy^7,9–11^ ) which have been termed “traditional/classical” with 4f^*n*+1^ ground states.^4,5^ The “non-traditional/non-classical” divalent lanthanide ions have driven interest in their physical properties and bonding in several areas including molecular magnetism,^12–16^ quantum information science,^17–20^ and metal–metal bonding,^14,16,21^ principally due to their unique singularly occupied 5d_z^2^_ orbital. With few exceptions,^15,22^ complexes containing “non-traditional/non-classical” divalent lanthanides reported in the literature are supported by either substituted Cp ligand fields,^13,14,16,21,23,24^ 3-fold coordination geometries,^18,25–27^ or often both.^1–3,10,19,28–30^ These complexes stabilize occupation of the 5d_z^2^_ orbital and result in 4f^n^5d_z^2^_^1^ ground states (with symmetry and energy dependent mixing with the 6s valence orbital).^13,17,18,24^ Only one ligand system, [La­(NHAr^iPr6^)_2_] (NHAr^iPr6–^ = [N­(H)­C_6_H_3_-2,6-(C_6_H_2_-2,4,6-*i*Pr_3_)_2_]^−^),^22^ facilitates the stabilization of another 5d orbital, namely 5d_*x* ^2^–*y* ^2^_, in a La^2+^ complex. Realization of *O*_*h*_ symmetry in a 6-coordinate complex would be particularly exciting as it would favor the partial occupation of a ground T state.

Given current synthetic methodologies such a goal is quite challenging. However, prior group work sought to realize pseudo-octahedral six-coordinate geometries by divalent lanthanides with the bis­(tris-*tert*-butoxysilyl)­amide (BTTSA) ligand.^31^ In contrast to the work of Mills and Murugesu, who have both applied bulky, monodentate, silylamide ligands to stabilize near-linear, pseudoaxial coordination environments,^32–37^ BTTSA complexes of divalent lanthanides yield pseudo-octahedral geometry wherein the neutral *tert*-butoxy groups coordinate around the equatorial plane of the complexed ion, and each ligand contributes two *tert*-butoxy and one amide donor with near linear N–Ln–N bond angles.^31^ Notably, though, both complexes featured abnormally long Ln–N distances, significantly distorting the primary 6-fold coordination sphere. Additionally, the poor crystallinity of BTTSA and its high affinity for potassium ions limited its synthetic utility to lanthanide systems amenable to stable isolation via redox transmetalation between the KCl-occluded Cu^+^ salt of BTTSA and the corresponding Ln^0^ metal, namely the similarly sized Sm and Eu.^31^

Therefore, careful redesign of the ligand backbone was undertaken to maintain the primary coordination sphere of BTTSA, while allowing for more flexibility toward the desired six-coordinate setting, improved crystallinity, and broader synthetic utility. We also sought to improve upon the electrochemical instability shown in some known bulky silylamide ligands^33,36^ to develop a more robust, redox-innocent ligand for stabilizing highly reducing divalent lanthanides, with the goal of eventually applying this system to the “non-classical/non-traditional” divalent ions. Herein we present progress toward these synthetic goals through the synthesis and characterization of the novel bis­(*tert*-butoxydiphenylsilyl)­amide ligand and its coordination complexes with the “traditional/classical” divalent lanthanides Sm^2+^, Eu^2+^, Tm^2+^, and Yb^2+^.

## Results and Discussion

### Synthesis and Structure

Synthesis of the protonated bis­(*tert*-butoxydiphenylsilyl)­amine (**1-H**) is shown in Scheme 1 and occurs in two steps from the literature compound *tert*-butoxychlorodiphenylsilane.^38^ Deprotonation of **1-H** with potassium benzyl then affords the potassium amide salt **1-K** in high yield. Single-crystal X-ray diffraction (SC-XRD) confirms the chemical identities of **1-H** and **1-K** (See Supporting Information (SI) for more details). In contrast to the previous generation BTTSA ligand,^31^ **1-K** readily undergoes salt metathesis with [LnI_2_(THF)_2_] (Ln = Sm, Eu, Yb; THF = tetrahydrofuran) to give the “classical/traditional” divalent lanthanide complexes **2-Ln** in good yields. Bulk purity for the isolated **2-Ln** complexes along with the ligand precursors **1-H** and **1-K** was established via elemental analysis and IR and NMR spectroscopies (*vide infra*). Attempts to synthesize the analogous Tm^2+^ species via *in situ* generation of [TmI_2_(DME)_*x*_] (DME = 1,2-dimethoxyethane) and salt metathesis with **1-K** affords an intractable mixture from which crystals of divalent **2-Tm** and the reduced dinitrogen, bimetallic complex **3-Tm** could both be identified by SC-XRD.

**Scheme 1:**
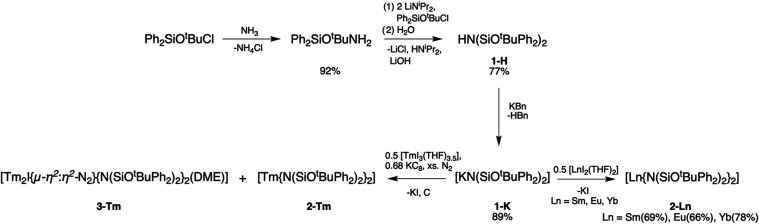
Synthetic Scheme for Ligand Derivatives **1-H**, **1-K**, and Classical Divalent Lanthanide Complexes **2-Ln** (Ln = Sm, Eu, Yb), Along with Reaction Conditions Leading to an Inseparable Mixture of **2-Tm** and the Reduced Dinitrogen Complex **3-Tm**

Crystallization of **2-Ln** (Ln = Sm, Eu, Yb) from concentrated solutions in toluene layered with pentane or hexane at −35 °C (see SI for more details) affords isotypic, strongly colored crystals (Sm: dark yellow-orange, Eu: fluorescent green, Yb: golden yellow) in the space group *P*1̅ (Figure 1). Crystals of **2-Tm** (along with crystals of **3-Tm**) were obtained from a concentrated solution of the crude, filtered reaction mixture in DME layered with pentane as strongly colored yellow-orange crystals which revealed a structure isotypic to that of the other **2-Ln** species.

**Figure 1:**
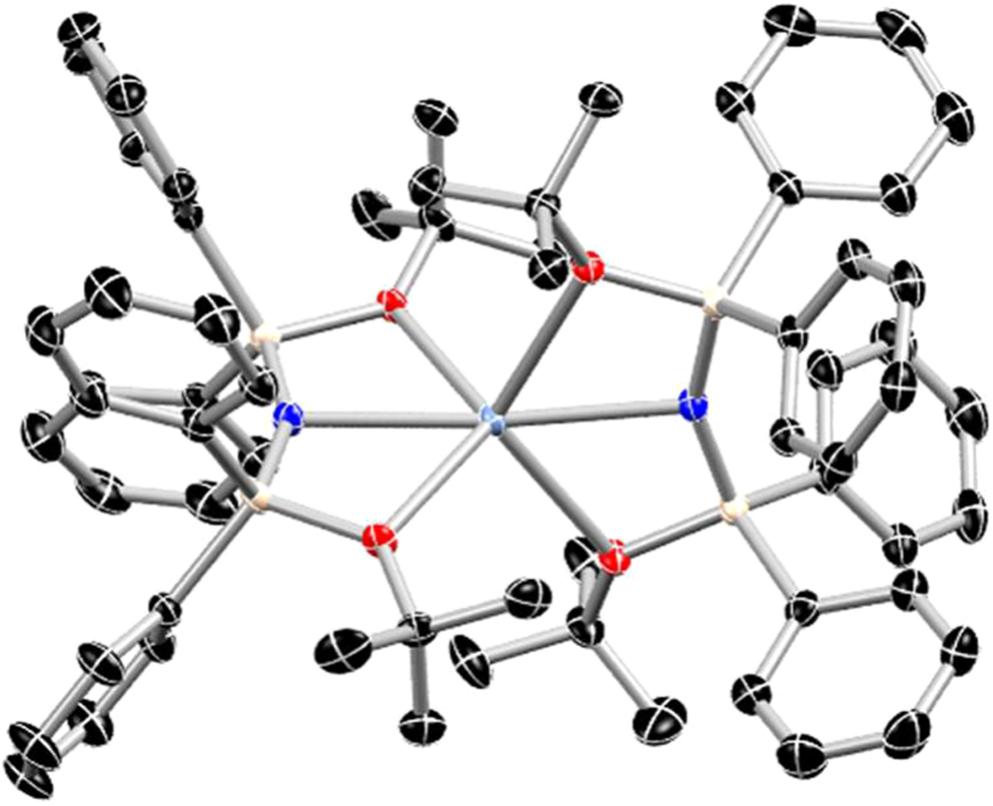
Representative molecular structure of **2-Eu** with thermal ellipsoids at 50%. Black, red, blue, tan, and blue-gray represent C, O, N, Si, and Eu respectively and hydrogen atoms and disordered solvent molecules omitted for clarity. **2-Ln** are isotypic.

The metal centers in the **2-Ln** complexes are coordinated by one amide nitrogen and two neutral oxygen atoms from the *tert*-butoxy groups of each ligand affording an overall six-coordinate metal ion. Bond lengths and angles for these compounds are given in Table 1. The average Ln–N and Ln–O bond lengths for **2-Ln** decrease across the series from Sm to Yb, consistent with the corresponding decrease in six-coordinate ionic radii for the respective metals (Sm^2+^: 1.19 Å, Eu^2+^: 1.17 Å, Tm^2+^: 1.03 Å, Yb^2+^: 1.02 Å).^39–41^ Excepting [Ln­(BTTSA)_2_] compounds with their unusually long Ln–N bond distances, the Ln–N bond lengths for **2-Ln** are longer than the reported range for similar bis­(bis-silylamide) complexes of divalent lanthanides (Sm–N: 2.432(13)-2.532(4) Å,^32,42,43^ Eu–N: 2.443(1)-2.531 Å,^33,36,44,45^ Tm–N: 2.363(3)-2.383(3) Å,^33^ Yb–N: 2.340(6)-2.425(1) Å ^33,35,43,45^ ).

**Table 1:** Selected Structural Metrics for **2-Ln**

	Ln–N_ave_ (Å)	Ln–O_ave_ (Å)	N–Ln–N (°)
**2-Sm**	2.541(3)	2.659(4)	174.2(1)
**2-Eu**	2.540(3)	2.648(4)	175.1(1)
**2-Tm**	2.428(3)	2.586(3)	176.6(1)
**2-Yb**	2.429(3)	2.575(4)	176.3(1)

Similarly, when compared to other complexes of divalent lanthanides with coordinated *tert*-butoxysilyl groups, the Ln–O bond lengths for **2-Ln**, with the exception of **2-Sm**, are at the higher end of, or greater than, the reported range (Sm–O: 2.519(2)-2.881(4) Å,^31,46–50^ Eu–O: 2.517(7)-2.638(2) Å,^31,48^ Yb–O: 2.405(4)-2.571(5) Å ^47,48,51^ ) with no reported species featuring this coordination environment for Tm^2+^. The N–Ln–N bond angles in **2-Ln** are also nearly linear (174.2(1)-176.6(1) °), and akin to other bulky bis­(bis-silylamide) complexes of divalent lanthanides, like [Ln­(BTTSA)_2_],^31^ [Ln­(N^††^)_2_] (N^††–^ = [N­(Si­(^i^Pr)_3_)_2_]^−^),^32,33^ and [Ln­(N­(SiPhMe_2_)_2_)_2_],^36,35^ highlighting the importance of steric bulk to designing pseudoaxial amide complexes.

Continuous shape measure (CShM) analysis as implemented in the SHAPE 2.1 program was employed to evaluate the coordination geometries of the lanthanide ions in **2-Ln**.^52,53^ In this analysis, deviations from all possible idealized coordination geometries for the primary coordination sphere are quantified on a scale from 0 to 100 where 0 corresponds to the undistorted geometry and the lowest value indicating the closest match based upon the bond distances and angles from the central metal ion.^52^ From this analysis, the first coordination spheres of **2-Sm** (14.529), **2-Eu** (14.738), **2-Tm** (13.742), and **2-Yb** (13.323) are best represented as distorted trigonal prismatic, which corresponds to a pseudo-*D*_*3h*_ geometry about the lanthanide ions (See Table S4 for full results). These relatively large values indicate significant geometric distortions from *D*_*3h*_ symmetry, which itself is already far descended from the desirable *O*_*h*_ setting. It is also important to note that this analysis does not consider the disparate identities of the donor atoms, providing only a measure of the polyhedral geometries present rather than the formal point group symmetries. To facilitate comparison with the previous generation [Ln­(BTTSA)_2_] complexes,^31^ CShM analysis was also performed on those structures and indicates that the primary coordination spheres [Sm­(BTTSA)_2_] (15.154) and [Eu­(BTTSA)_2_] (14.904) are also best described as distortions of trigonal prismatic (See Table S5 for full results).

Crystals of the reduced dinitrogen, bimetallic thulium species **3-Tm** (Figure 2) were identified in the same crude mixture as **2-Tm** as visually indistinguishable yellow-orange crystals in the space group *P*1̅. The structure of **3-Tm** contains two unique Tm metal centers, each bearing a single [N­(Ph_2_SiO*t*Bu)_2_]^1–^ ligand and bridged by a η^2^-N_2_ species, with one metal binding an iodide ligand and the other, a chelating DME molecule. The major components of each metal center display similar bond distances to the [N­(Ph_2_SiO*t*Bu)_2_]^1–^ ligand, with an average Tm–N bond distance of 2.294(7) Å and an average Tm–O bond distance of 2.491(10) Å, both meaningfully shorter than the corresponding distances for **2-Tm** and consistent with the decrease in six-coordinate ionic radius from Tm^2+^ to Tm^3+^ (1.03 vs 0.88 Å).^39^ This metric implies both metal centers are trivalent despite the asymmetric primary coordination spheres. Additionally, in the bridging dinitrogen ligand, the N–N bond distance of 1.413(9) Å is longer than that of reported side-on bound N_2_^2–^ species bound to Tm^3+^ (1.19(4)-1.261(3) Å).^54–57^ Rather, it is in line with distances reported for N_2_^3–^ species bound to other Ln^3+^ ions (1.36(1)-1.449(4) Å).^58–64^ Based upon the contracted Tm–N and Tm–O bonds (in comparison to **2-Tm**) and the long N–N distance in the bridging dinitrogen, the core of **3-Tm** is assigned as two Tm^3+^ centers bridged by a side-on N_2_^3–^ radical species, the second structurally identified compound of this type.^57^ Interestingly, as compared to the first example of this type recently reported by Mazzanti and co-workers, the N–N bond distance for **3-Tm** is significantly longer than their reported distance of 1.22(6) Å,^57^ likely due to a combination of differences in ancillary ligand donor capacity, supporting electrostatic interactions, and overall complex sterics.

**Figure 2:**
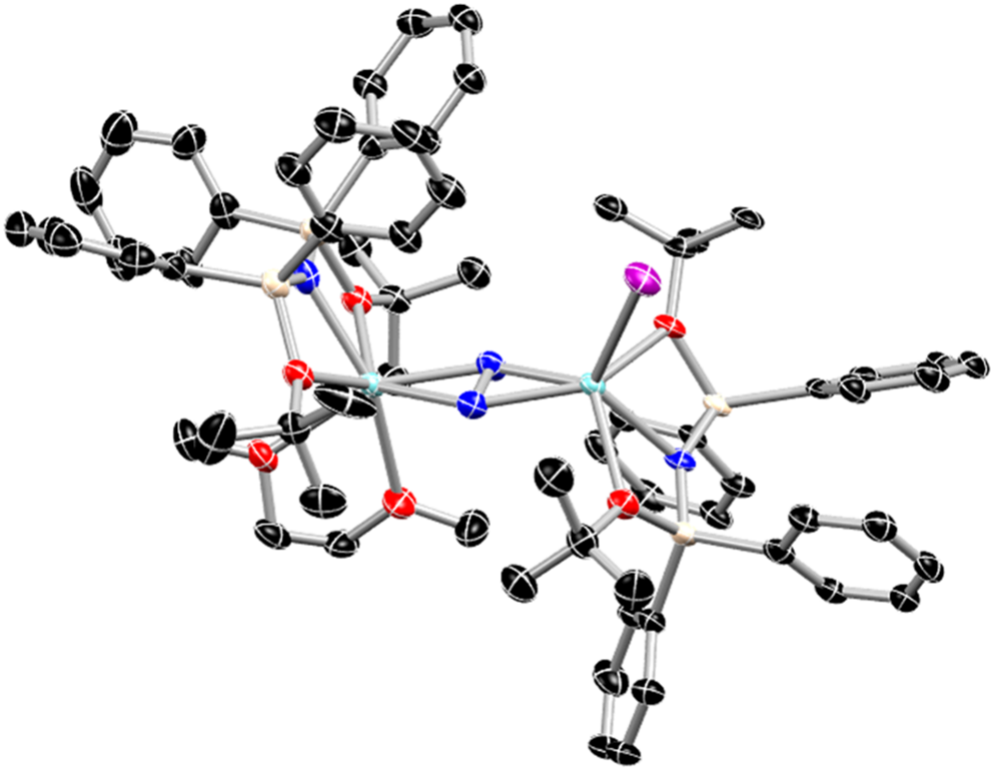
Molecular structure of **3-Tm** with thermal ellipsoids at 50%. Black, red, blue, tan, purple, and cyan represent C, O, N, Si, I, and Tm respectively and hydrogen atoms, noncoordinated solvent molecules, and minor disordered components omitted for clarity.

### NMR Spectroscopy

The diamagnetic species **1-H**, **1-K**, and **2-Yb** were studied by ^1^H, ^13^C­{^1^H}, ^13^C-DEPT135, ^1^H–^13^C HSQC, and ^1^H–^13^C HMBC NMR spectroscopies and the spectra enabled full assignment of the proton and carbon resonances for these compounds. These techniques were also used to study **2-Sm**, which showed dramatic shifts in ^1^H and ^13^C resonances consistent with the paramagnetic nature of Sm^2+^. Excepting the quaternary –*C*(CH_3_)_3_ resonance, however, all expected features for **2-Sm** were observed and assignable within the windows ± 250 ppm. No ^1^H or ^13^C resonances could be found in the windows ± 250 ppm for the highly paramagnetic **2-Eu**.

^29^Si-DEPT24 spectra were also measured for these compounds, and revealed singlet features at δ_Si_ = −34.09 and −35.15 ppm for **1-H** and **2-Yb** respectively, with no ^29^Si resonances observed for the paramagnetic **2-Sm** and **2-Eu** in the window 300 to −500 ppm. Interestingly, no ^29^Si resonances could be identified either for **1-K** in this window, even in a saturated solution. The singlet features observed suggest no appreciable coupling between the Si nuclei and the central N atom in **1-H** and **2-Yb**, nor to the spin-half ^171^Yb nucleus in the case of **2-Yb** is present in these species. The ^29^Si resonance for **2-Yb** is also shifted upfield with respect to those of similar Yb^2+^ silylamide complexes such as [crypt­(K)]­[Yb­(N­(Si*^t^* BuMe_2_)_3_] (δ_Si_ = −12.55 ppm)^43^ and [Yb­(N^††^)_2_] (δ_Si_ = −8.81 ppm),^33^ suggesting a greater degree of electron density at the Si atoms, likely afforded by the *tert*-butoxy groups.

^171^Yb­{^1^H} NMR performed on **2-Yb** revealed a singlet feature at δ_Yb_ = 564.44 ppm referenced to the calibrated ^1^H spectrum (See SI for more details). Similar to the observed ^29^Si resonances, there is no evidence of coupling of the ^171^Yb nucleus to any other spin-active nuclei in the system. Compared to ^171^Yb resonances reported for similar compounds this shift sits between and reasonably close to those noted for [Yb­(N^††^)_2_] (δ_Yb_ = 467.46 ppm)^33,43^ and [crypt­(K)]­[Yb­(N­(Si*^t^* BuMe_2_)_3_] (δ_Yb_ = 419.14 ppm),^43^ and [Yb­(P­(Si^*i*^Pr_3_)_2_)_2_(THF)_2_] (δ_Yb_ = 682 ppm),^65^ suggesting a similar overall electronic environment for the Yb nucleus.

### Electronic Absorption Spectroscopy

The electronic absorption spectra for **1-H, 1-K**, and **2-Ln** (Ln = Sm, Eu, Yb) were collected to further validate the lanthanide oxidation states assigned by crystallography. The UV–vis–NIR spectra of these species were recorded from 250–1500 nm on solutions of the complexes in THF (Figures S45–S49). Abridged spectra are shown in Figure 3 due to the lack of features above 850 nm in any of the compounds. The spectrum of the protonated ligand **1-H** is mostly featureless, with one major double peak feature at 260 and 264 nm, with extinction coefficients of 1040 and 1050 M^–1^ cm^–1^ respectively, and is tentatively assigned as originating from π → π* transitions within the phenyl substituents of the ligand backbone. The spectrum of **1-K** is similarly sparse with no discernible features, likely due to a hypsochromic shift of the π → π* transitions of the ligand into the UV cutoff of THF.

**Figure 3:**
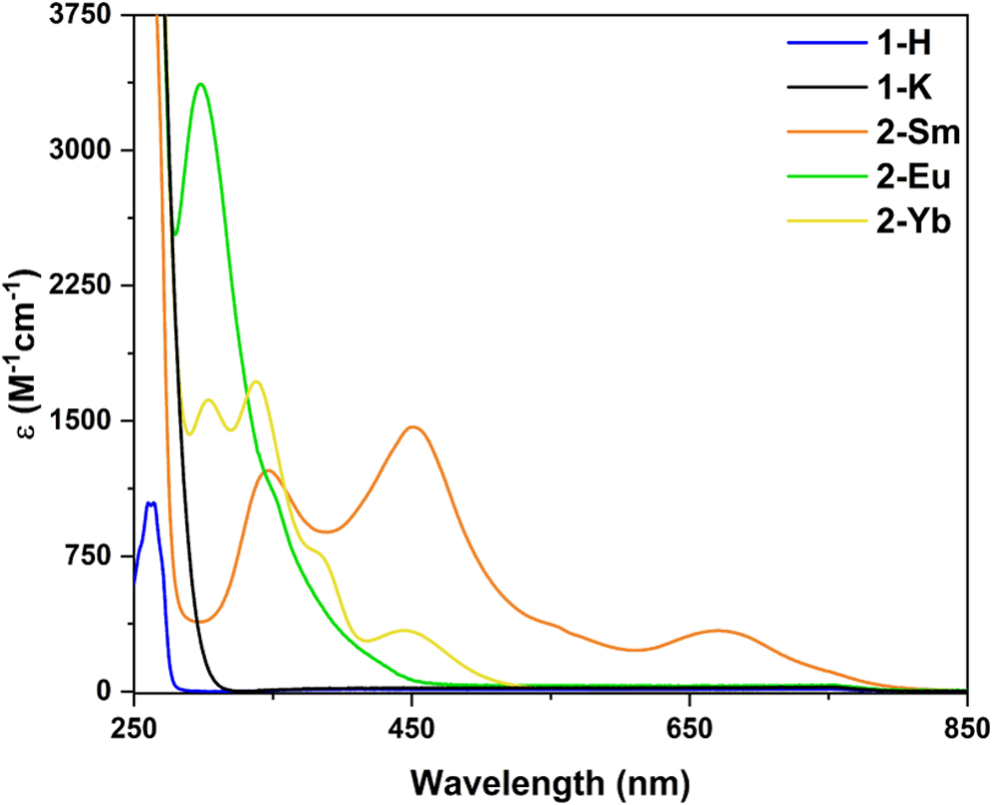
UV–vis spectra for **1-H**, **1-K**, and **2-Ln** (Ln = Sm, Eu, Yb) recorded in THF.

The spectra obtained for **2-Ln** (Ln = Sm, Eu, Yb) bear a strong resemblance to those observed for other near-linear bis-silylamide complexes of “classical/traditional” divalent lanthanides, namely [Ln­(BTTSA)_2_] and [Ln­(N^††^)_2_],^31,33^ indicating a similar crystal field environment imposed on the metal ions. The features observed in these spectra are tentatively assigned based upon similarities to other relevant systems,^31,33^ the proximity of the transition energies to known f-f transition energies,^66^ and the co-occurrence of features across various spectra. For **2-Yb**, three main peaks are observed at 303, 338, and 445 nm, with extinction coefficients of 1610, 1720, and 338 M^–1^ cm^–1^ respectively, along with a shoulder peak around 380 nm. The 4f^14^ configuration of Yb^2+^ precludes assigning these transitions as f-f in origin, and thus they likely originate from other processes, such as ligand-to-metal charge transfers (LMCTs) or spin-allowed f-d transitions.

The UV–vis spectrum of **2-Sm** similarly displays three main peaks at 347, 451, and 670 nm with extinction coefficients of 1220, 1470, and 337 M^–1^ cm^–1^ respectively. The broader low intensity feature at 670 nm has been previously assigned as a ^7^F_0_ → ^5^D_0_ transition,^66^ while the strongest feature at 451 nm has been attributed to a combination of ^7^F_0_ → ^5^D_1_ and ^7^F_0_ → ^5^D_2_ transitions due to their similar energies.^31,33,66^ These features though are quite intense and broad for f-f transitions and may instead arise from an LMCT or spin-allowed f-d transition due to the proximity of this transition to that observed at 445 nm in **2-Yb**. The highest energy feature at 347 nm is close in energy to the feature at 338 nm for **2-Yb** and above the energies for which pure f-f transitions are observed for Sm^2+^, indicating this feature originates from an LMCT or spin-allowed f-d transition.^66,67^

**2-Eu** displays a single strong peak centered at 298 nm with an extinction coefficient of 3370 M^–1^ cm^–1^ with a shoulder peak around 350 nm and a low intensity background feature beginning around 800 nm. The features at 298 and 350 nm have previously been assigned as ^8^S_7/2_ → ^6^I_7/2_ and ^8^S_7/2_ → ^6^P_7/2_ transitions, respectively.^66^ The strong relative intensity of these features despite their formal Laporte-forbidden nature has been ascribed to vibronic coupling in similar near-linear bis-silylamide complexes,^31,33^ though due to their high extinction coefficients and proximity to **2-Yb** features at 303 and 338 nm, may actually arise from LMCTs or spin-allowed f-d transitions.

### Electrochemistry

To gauge the redox stabilization of the divalent oxidation state in the isolated **2-Ln** species, cyclic voltammetry was performed on fluorobenzene (PhF) solutions of **2-Ln** at multiple scan rates (Figures 4 and S50–S52). Both **2-Sm** and **2-Eu** demonstrate quasi-reversible redox couples with *E*_1/2_ values of −1.45 V (Δ*E*_p_ = 489 mV) and −0.27 V (Δ*E*_p_ = 502 mV) vs Fc^+/0^ respectively. By contrast, **2-Yb** demonstrates an irreversible oxidation with an *E*_pa_ of −0.85 V vs Fc^+/0^. The noncoincidence of the voltammograms for the three species suggests the observed features correspond to metal- rather than ligand-centered redox couples, as contrasted to the ligand-centered oxidations noted for [Ln­(N^††^)_2_],^33^ and are assigned as the respective Ln^3+/2+^ couples for **2-Sm** and **2-Eu** and an Yb^2+^ → Yb^3+^ oxidation for **2-Yb**. The relative ordering of the potentials (Eu^2+^ < Yb^2+^ < Sm^2+^) aligns with that of the calculated standard aqueous potentials (Eu^3+/2+^: −0.75 V; Yb^3+/2+^: −1.55 V; Sm^3+/2+^: −1.95 V vs Fc^+/0^; Fc^+/0^: 0.40 V vs NHE).^68,69^

**Figure 4:**
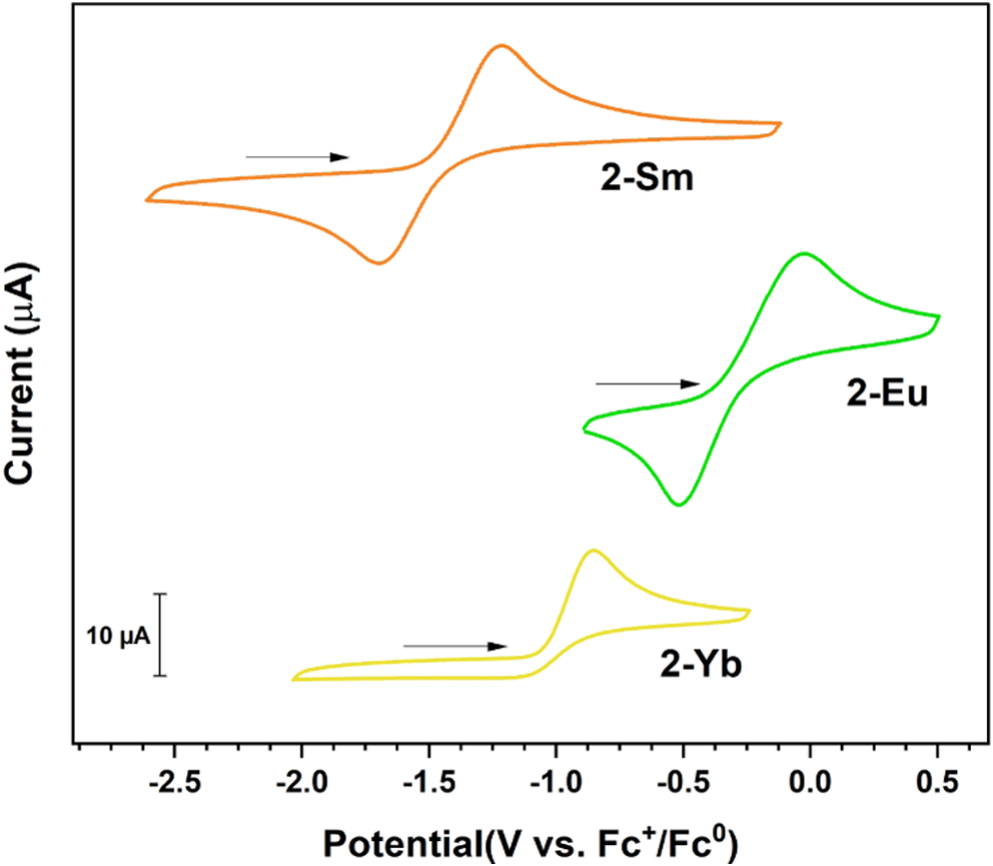
Cyclic voltammograms of 1.9 mM **2-Sm** (top), 2.0 mM **2-Eu** (middle), and 2.0 mM **2-Yb** (bottom) in 200 mM [N­(^*n*^Bu)_4_]­[PF_6_] at 200 mV/s. Additional details in SI.

Compared to other compounds, the observed potentials for **2-Ln** are significantly less negative than those reported for the respective Ln^3+/2+^ couples (*E*_1/2_) for [LnCp_3_] (Ln = Sm: −2.66, Eu: −1.55, Yb: −1.94 V vs Fc^+/0^),^70^ [Ln­(Cp′)_3_] (Cp′^–^ = 1-trimethylsilylcyclopentadienyl) (Ln = Sm: −2.41, Eu: −1.07, Yb: −1.64 V vs Fc^+/0^),^71^ and [Ln­(Cp*)_2_·MeCN] (Ln = Sm: −2.41, Eu: −1.22, Yb: −1.78 V vs Fc^+/0^)^72^ complexes indicating a significant thermodynamic stabilization of the divalent oxidation state for **2-Ln** as compared to these (pseudo-)­3-fold cyclopentadienyl coordination environments. The more recently reported [Ln­(Cp^iPr5^)_2_] (Cp^iPr5–^ = 1,2,3,4,5-pentaisopropylcyclopentadienyl) (Ln = Sm: *E*_1/2_ = −1.14, Eu: *E*_pa_ = −0.29, Yb: *E*_1/2_ = −0.51 V vs Fc^+/0^)^24^ species however afford even less negative potentials for the respective Ln^3+/2+^ couples (with the exception of [Eu­(Cp^iPr5^)_2_]), highlighting the impact of differing numbers of bulky anionic ligands in driving Ln^3+/2+^ potentials.

The irreversible oxidation of **2-Yb** is intriguing given the intermediate value of the corresponding *E*_pa_ compared with that of **2-Eu** and **2-Sm**, suggesting that size of the trivalent cation may be the deciding factor in the stability of the trivalent product of oxidation, with the ligand unable to adequately bind to the Yb^3+^ with its smaller six-coordinate ionic radius of 0.87 Å.^39^ While all three redox couples are thermodynamically accessible, the electrochemical oxidation of **2-Yb** results in a species that is kinetically unstable in the reaction conditions.

Attempts to chemically oxidize **2-Yb** with silver tetrakis­(perfluorophenyl)­borate (AgBArF_20_) in THF yielded a mixture of **2-Yb** and **1-H** by ^1^H NMR, indicating that the oxidized Yb^3+^ species is chemically unstable under these conditions (Figure S37). Further attempts to chemically oxidize **2-Yb** were also performed in PhF using the milder oxidant ferrocenium tetrakis­(perfluorophenyl)­borate (FcBArF_20_) (Ag^+/0^ = 0.41 V vs Fc^+/0^ in THF).^69^ ^1^H NMR analysis of the crude product of this reaction indicates formation of **1-H** and multiple broad resonances that could not be clearly assigned as a singular product (Figure S38).

To investigate if the source of this irreversible event was in fact due to a ligand-based oxidation, cyclic voltammetry was also performed on **1-K** under identical conditions as **2-Ln** (Figures S54 and S55). Only a single irreversible oxidation is observed for **1-K** with an *E*_pa_ of 0.00 V vs Fc^+/0^, far more positive than the oxidation observed at −0.85 V for **2-Yb**. This potential is also significantly more positive than those noted for the ligand-based oxidations of [Ln­(N^††^)_2_] whose *E*_pa_ values range from −0.59 V (Ln = Yb) to −0.54 V (Ln = Sm) vs Fc^+/0^.^33^ Though very close to the oxidative peak of **2-Eu** (*E*_pa_ = −0.02 V vs Fc^+/0^), no distinct ligand oxidation event at 0 V vs Fc^+/0^ is evident for any of the **2-Ln** species, suggesting coordination imparts some electrochemical stability to the ligand. The apparent kinetic instability of the oxidation product of **2-Yb** is thus not clearly the result of secondary redox events from the ligand and likely highly dependent upon reaction conditions and subsequent reactions in the reaction mixture.

### Direct-Current (DC) Magnetometry

The magnetic ground states of **2-Sm** and **2-Eu** were investigated by variable-temperature and -field direct-current (DC) magnetometry (Figures S64–S67). The coplot of χT vs T curves for **2-Sm** and **2-Eu** measured at 1 T are shown in Figure 5. The room temperature moment for **2-Eu** is 7.90 emu·K/mol which agrees well with the expected moment of 7.88 emu·K/mol for an isotropic 4f^7^ ion.^73^ The near constant moment over much of the range investigated is consistent with a singular ^8^S_7/2_ ground state with the sudden decrease in moment as temperatures approach 0 K often attributed to zero-field splitting (ZFS) in other Eu^2+^ systems.^33,36,43,74^

**Figure 5:**
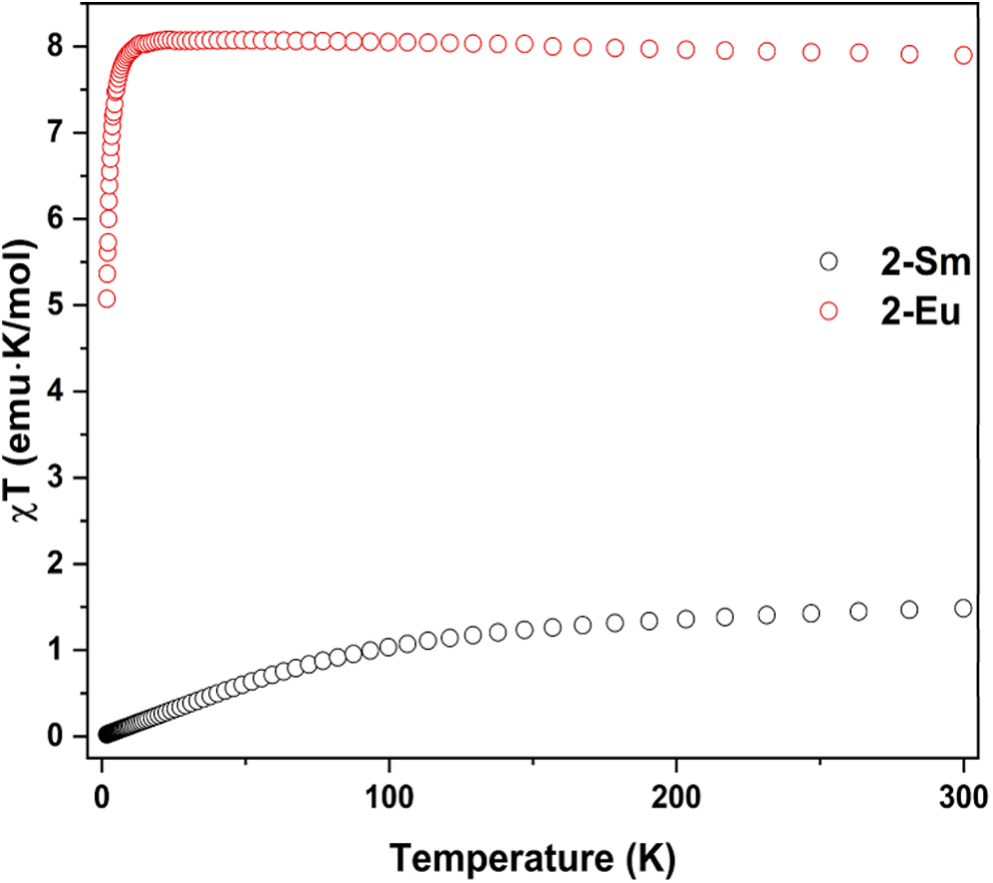
Variable-temperature zero-field cooled magnetic susceptibility curves for **2-Sm** and **2-Eu** measured under an applied field of 1 T.

Formally, the ^7^F_0_ ground state of divalent samarium (4f^6^) affords zero magnetic moment. However, thermal population of excited states imparts moment onto the system, affording an expected moment at room temperature of 1.46 emu·K/mol which trends toward 0 emu·K/mol with decreasing temperature and thermal population.^67,73^ This behavior is clearly seen for **2-Sm** which shows a moment that trends to 0 emu·K/mol at low temperature but increases to 1.60 emu·K/mol at 300 K. Though slightly larger than the expected value and that noted for [Sm­(BTTSA)_2_],^31^ this moment is in good agreement with the reported value for [Sm­(N^††^)_2_] of 1.64 emu·K/mol.^32^

## Conclusions

The development of the bis­(*tert*-butoxydiphenylsilyl)­amide ligand represents progress toward the goal of implementing the pseudo-octahedral coordination sphere of the previous generation BTTSA ligand with improved synthetic versatility. In contrast to BTTSA, this ligand system readily undergoes salt metathesis with [LnI_2_(THF)_2_] (Ln = Sm, Eu, Yb) to form complexes featuring six-coordinate Ln^2+^ ions whose spectroscopic and magnetic properties are in line with those of the corresponding [Ln­(BTTSA)_2_] complexes and strikingly similar to the corresponding [Ln­(N^††^)_2_] species. These spectroscopic and magnetic properties are consistent with the expected 4*f* ^*n*+1^ ground states of these “classical/traditional” divalent ions. Electrochemical data indicates a ligand framework with increased redox stability in the windows of relevant Ln^3+/2+^ potentials, with some size-related instability for **2-Yb**. Attempts to extend this work to the next most easily reduced lanthanide, Tm, afforded an intractable mixture containing crystals of the analogous pseudo-octahedral **2-Tm**, and **3-Tm** a reduced dinitrogen, bimetallic Tm^3+^-Tm^3+^ complex bridged by a η^2^-N_2_^3–^ radical.

## Experimental Section

Synthetic details for reported compounds are given below. General considerations, NMR, IR, and UV–vis–NIR spectra, and details regarding electrochemistry, crystallographic solution and refinement, and magnetometry are reported in the SI.

**CAUTION!** *The use of Schlenk techniques carries significant risks including the condensation of liquid oxygen from ambient air when operating with traps at cryogenic temperatures. Readers should familiarize themselves with standard Schlenk line technique and cryogen safety prior to attempting these syntheses. Ammonia is a highly corrosive, noxious gas and should always be handled in a well-ventilated fume hood with care taken to guard against potential exposure. The reagents lithium diisopropylamide (LDA), potassium benzyl (KBn), and potassium graphite (KC*_8_ *) are pyrophoric, and care should be taken to guard against accidental exposure to oxygen or water.*

### Ph_2_SiO*^t^* BuNH_2_

This compound was synthesized following a modified literature procedure.^31^ In the glovebox, a 1 L round-bottom Schlenk flask was charged with Ph_2_SiO*^t^* BuCl (10.986 g, 37.77 mmol), diethyl ether (Et_2_O) (100 mL), and a PTFE coated magnetic stirring bar. The flask was then sealed, removed from the glovebox, and cycled onto a Schlenk line. While stirring, anhydrous ammonia was then bubbled through the clear solution for 20 min and the flask was sealed under a slight positive pressure of ammonia and the reaction mixture was stirred overnight at room temperature. This bubbling procedure was repeated twice more, and the reaction mixture was stirred for 6.5 and 16.5 h for the second and third dosings, respectively. After these rounds of bubbling and stirring, the flask was then purged with argon for 20 min to flush out excess ammonia. Volatiles were removed *in vacuo*, leaving a white residue that was then suspended in 1:1 hexanes: Et_2_O (total volume, 80 mL) and filtered through a pad of Celite on a glass frit. The clear filtrate was reduced *in vacuo* to give a cloudy, white oil which was used without further purification (9.480 g, 92%). ^1^H NMR (400 MHz, C_6_D_6_): δ 7.79–7.82 (m, 4H, *m*-aryl), 7.17–7.23 (m, 6H, *o*-aryl/*p*-aryl), 1.30 (s, 9H, –*^t^* Bu), 0.95 (bs, 2H, -NH_2_). ^13^C­{^1^H} NMR (101 MHz, C_6_D_6_): δ 138.56 (*i*-aryl), 135.12 (*m*-aryl), 129.71 (*p*-aryl), 127.93 (*o*-aryl), 73.39 (–*C*(CH_3_)_3_), 32.34 (–C­(*C*H_3_)_3_). ^29^Si-DEPT24 (79 MHz, C_6_D_6_): δ −33.22 (s). IR (cm^–1^): *v* 3479 (vw), 3397 (vw), 3068 (vw), 3049 (vw), 2972 (m), 2929 (vw), 2870 (vw), 1590 (vw), 1545 (w), 1485 (vw), 1469 (vw), 1428 (w), 1388 (vw), 1363 (w), 1305 (vw), 1239 (m), 1194 (s), 1112 (s), 1045 (vs), 1022 (vs), 998 (m), 853 (s), 806 (s), 738 (s), 698 (vs), 647 (m), 514 (s), 485 (s), 455 (s), 416 (m). Elemental analysis was not collected on this air sensitive liquid.

### HN­(Ph_2_SiO*^t^* Bu)_2_ (**1-H**)

In the glovebox, a 500 mL round-bottom Schlenk flask was charged with Ph_2_SiO*^t^* BuCl (6.173 g, 21.22 mmol), Ph_2_SiO*^t^* BuNH_2_ (5.759 g, 21.22 mmol, 1.0 equiv), hexanes (200 mL), and a PTFE coated magnetic stirring bar. To this colorless mixture was then added a solution of lithium diisopropylamide (LDA) (4.546 g, 42.44 mmol, 2.0 equiv) in THF (10 mL), before the flask was sealed, removed from the glovebox, and cycled onto a Schlenk line. The flask was cooled in an ice bath (0 °C) and allowed to stir for 1 h before removing the ice bath and the reaction mixture was allowed to warm to room temperature and to continue stirring overnight. The resulting pale-yellow solution was reduced *in vacuo* and the waxy yellow-white residue was resuspended in hexanes (200 mL). To this suspension was then added a solution of H_2_O (0.38 mL, 21.22 mmol, 1.0 equiv) in THF (20 mL), and the reaction mixture was stirred overnight. The colorless solution was reduced *in vacuo* and the white residue triturated with pentane (3 × 10 mL). Recrystallization of the residue in Et_2_O at −35 °C affords the product as large, colorless crystals suitable for XRD analysis (8.559 g, 77%). ^1^H NMR (400 MHz, THF-*d*_8_): δ 7.56–7.58 (m, 8H, *m*-aryl), 7.25–7.29 (m, 4H, *p*-aryl), 7.18–7.22 (m, 8H, *o*-aryl), 2.60 (bs, 1H, –NH), 1.17 (s, 18H, *^t^* Bu). ^13^C­{^1^H} NMR (101 MHz, THF-*d*_8_): δ 139.18 (*i*-aryl), 136.05 (*m*-aryl), 129.79 (*p*-aryl), 127.90 (*o*-aryl), 74.03 (–*C*(CH_3_)_3_), 32.39 (–C­(*C*H_3_)_3_). ^29^Si-DEPT24 (79 MHz, THF-*d*_8_): δ −34.09 (s). IR (cm^–1^): *v*. 3367 (vw), 3069 (vw), 3038 (vw), 3022 (vw), 2974 (m), 2928 (vw), 2901 (vw), 2867 (vw), 1956 (vw), 1885 (vw), 1816 (vw), 1655 (vw), 1590 (vw), 1486 (vw), 1461 (vw), 1428 (m), 1388 (w), 1363 (m), 1331 (vw), 1303 (vw), 1241 (w), 1192 (m), 1167 (s), 1110 (s), 1044 (s), 1023 (s), 998 (m), 966 (w) 921 (s), 817 (m), 758 (m), 734 (s), 693 (vs), 654 (m), 627 (w), 537 (s), 521 (w), 485 (s), 459 (m), 424 (w). Elem anal. Found (calculated) for C_32_H_39_NO_2_Si_2_: C, 73.88 (73.09); H, 7.58 (7.48); N, 2.83 (2.66). Carbon consistently high on duplicate analyses.

### [KN­(Ph_2_SiO*^t^* Bu)_2_] (**1-K**)

In the glovebox, a 20 mL scintillation vial was charged with potassium benzyl (KBn) (0.208 g, 1.60 mmol), toluene (1 mL), and a PTFE coated magnetic stirring bar. To this bright red-orange suspension was added a solution of **1-H** (0.800 g, 1.52 mmol, 0.95 equiv) in toluene (17 mL) and the reaction mixture was then stirred for 1.5 h at room temperature during which the red-orange color of KBn disappeared and a whitish precipitate formed. This off-white precipitate was then isolated on a fine porosity glass frit, washed with hexanes (20 mL), and reduced *in vacuo* on the frit. The precipitate was redissolved on the frit in THF (5 mL) and the resulting yellow solution filtered to yield a black solid material and a colorless filtrate. The colorless filtrate was reduced *in vacuo* and the white solids triturated with pentane (2 × 1 mL) to afford the product as a white powder. (0.767 g, 89%). XRD quality single crystals were grown from either vapor diffusion of hexanes into a concentrated solution of product in THF at −35 °C (Method A) or a concentrated solution of product in DME at −35 °C (Method B), affording two distinct polymorphs. ^1^H NMR (400 MHz, THF-*d*_8_): δ 7.72–7.74 (m, 8H, *m*-aryl), 7.06–7.09 (m, 12H, *o*-aryl/*p*-aryl), 1.14 (s, 18H, *^t^* Bu). ^13^C­{^1^H} NMR (101 MHz, THF-*d*_8_): δ 147.58 (*i*-aryl), 136.32 (*m*-aryl), 127.38 (*p*-aryl), 127.07 (*o*-aryl), 71.49 (–*C*(CH_3_)_3_), 32.87 (–C­(*C*H_3_)_3_). No ^29^Si resonance(s) could be identified for this compound in the window 300 ppm to −500 ppm. IR (cm^–1^): *v*. 3062 (vw), 3044 (vw), 3021 (vw), 2972 (vw), 2961 (vw), 2867 (vw), 2171 (vw), 1472 (vw), 1426 (w), 1388 (vw), 1364 (w), 1317 (m), 1279 (m), 1257 (w), 1237 (w), 1178 (m), 1100 (m), 1062 (vw), 1027 (w), 983 (m), 955 (m), 906 (w), 868 (vw), 793 (w), 746 (w), 701 (m), 686 (m), 650 (w), 637 (w), 620 (w), 525 (m), 499 (m), 484 (w), 473 (m), 434 (w), 407 (w). Elem anal. Found (calculated) for C_32_H_38_KNO_2_Si_2_: C, 66.08 (68.16); H, 6.91 (6.79); N, 2.44 (2.48). Carbon consistently low on duplicate analyses.

### [Sm­(N­(Ph_2_SiO*^t^* Bu)_2_)_2_] (**2-Sm**)

In the glovebox, a 20 mL scintillation vial was charged with [SmI_2_(THF)_2_] (0.1202 g, 0.219 mmol), THF (1 mL), and a glass coated magnetic stirring bar. To this mixture was added a solution of **1-K** (0.2469 g, 0.438 mmol, 2.0 equiv) in THF (7 mL), and the resulting dark brown reaction mixture was stirred for 18 h at room temperature. The mixture was then filtered through a 1 cm pad of Celite on a medium porosity glass frit, and the white solids were washed with additional THF (5 mL). The resulting dark brown filtrate was then reduced *in vacuo* and triturated with pentane (3 × 2 mL) to afford a dark brown residue. The residue was then recrystallized twice from a concentrated solution in toluene layered with pentane at −35 °C, washed with minimal room temperature pentane (3 × 1 mL), crushed, and reduced *in vacuo* to afford the product as a dark brown, free-flowing powder (0.1821 g, 69%). XRD quality single crystals were grown from a concentrated solution in toluene layered with pentane at −35 °C. ^1^H NMR (400 MHz, THF-*d*_8_): δ 15.28 (s, 36H, *^t^* Bu), 5.73 (t, ^3^ *J*_HH_ = 8 Hz, 8H, *p*-aryl), 5.26 (t, ^3^ *J*_HH_ = 8 Hz, 16H, *o*-aryl), 0.78 (d, ^2^ *J*_HH_ = 4 Hz, 16H, *m*-aryl). ^13^C­{^1^H} NMR (101 MHz, THF-*d*_8_): δ 130.79 (*m*-aryl), 126.48 (*p*-aryl), 124.82 (*o*-aryl), 118.79 (*i*-aryl), 78.91 (–C­(*C*H_3_)_3_). No ^13^C peak correlated to the –*C*(CH_3_)_3_ resonance could be identified in the window of ± 250 ppm. No ^29^Si resonance(s) could be identified for this compound in the window 300 ppm to −500 ppm. IR (cm^–1^): *v*. 3065 (vw), 2966 (w), 1588 (vw), 1461 (vw), 1426 (w), 1391 (vw), 1366 (w), 1232 (m), 1171 (m), 1106 (m), 1029 (vw), 950 (s), 910 (m), 797 (m), 752 (m), 738 (m), 722 (m), 700 (vs), 659 (w), 631 (m), 619 (m), 520 (s), 497 (s), 463 (m), 423 (w). Elem anal. Found (calculated) for C_64_H_76_N_2_O_4_Si_4_Sm: C, 62.84 (64.06); H, 6.44 (6.38); N, 2.28 (2.33). Carbon consistently low on duplicate analyses.

### [Eu­(N­(Ph_2_SiO*^t^* Bu)_2_)_2_] (**2-Eu**)

In the glovebox, a 20 mL scintillation vial was charged with [EuI_2_(THF)_2_] (0.0915 g, 0.166 mmol), THF (1 mL), and a glass coated magnetic stirring bar. To this was added a solution of **1-K** (0.1880 g, 0.333 mmol, 2.0 equiv) in THF (5 mL), and the resulting bright yellow-green reaction mixture was stirred for 18 h at room temperature. The mixture was then filtered through a 1 cm pad of Celite on a fine porosity glass frit, and the white solids were washed with additional THF (5 mL). The clear, yellow-green filtrate was then reduced *in vacuo* and triturated with pentane (3 × 2 mL) to afford a pale green residue. The residue was then recrystallized from a concentrated solution in toluene layered with pentane at −35 °C, washed with minimal room temperature pentane (3 × 1 mL), crushed, and remaining volatiles removed *in vacuo* to afford the product as a pale green, free-flowing powder (0.1328 g, 66%). XRD quality single crystals were grown from a concentrated solution in toluene layered with hexane at −35 °C. No ^1^H or ^13^C resonances corresponding to this compound could be identified in the windows ± 250 ppm. No ^29^Si resonance(s) could be identified for this compound in the window 300 ppm to −500 ppm. IR (cm^–1^): *v*. 3065 (vw), 2966 (vw), 1588 (vw), 1463 (vw), 1426 (w), 1391 (vw), 1367 (w), 1235 (m), 1171 (m), 1105 (m), 1028 (vw), 948 (s), 911 (w), 797 (w), 752 (w), 738 (w), 722 (m), 700 (s), 657 (vw), 630 (m), 520 (s), 496 (s), 463 (m), 422 (w). Elem anal. Found (calculated) for C_64_H_76_EuN_2_O_4_Si_4_: C, 63.97 (63.97); H, 6.41 (6.38); N, 2.39 (2.33).

### [Yb­(N­(Ph_2_SiO*^t^* Bu)_2_)_2_] (**2-Yb**)

In the glovebox, a 20 mL scintillation vial was charged with [YbI_2_(THF)_2_] (0.0937 g, 0.164 mmol), THF (1 mL), and a glass coated magnetic stirring bar. To this mixture was added a solution of **1-K** (0.1854 g, 0.329 mmol, 2.0 equiv) in THF (5 mL), and the resulting golden-yellow reaction mixture was stirred for 19 h at room temperature. The mixture was then filtered through a 1 cm pad of Celite on a fine porosity glass frit, and the white solids were washed with additional THF (5 mL). The clear, intensely yellow-orange filtrate was then reduced *in vacuo* and triturated with pentane (3 × 2 mL) to afford a yellow-orange residue. The residue was then dissolved in minimal toluene, and an excess of pentane added to precipitate a white solid overnight at −35 °C. The solids were removed by filtration through a glass pipet packed with Celite, and the clear filtrate reconcentrated *in vacuo*, layered with pentane, and stored at −35 °C to afford bright yellow crystals. These crystals were washed with minimal room temperature pentane (3 × 1 mL), crushed, and remaining volatiles were removed *in vacuo* to yield the product as a yellow, free-flowing powder (0.1562 g, 78%). XRD quality single crystals were grown from a concentrated solution in toluene layered with hexane at −35 °C. ^1^H NMR (400 MHz, THF-*d*_8_): δ 7.37 (d, ^2^ *J*_HH_ = 8 Hz, 16H, *m*-aryl), 7.20 (t, ^3^ *J*_HH_ = 8 Hz, 8H, *p*-aryl), 7.02 (t, ^3^ *J*_HH_ = 8 Hz, 16H, *o*-aryl), 1.45 (s, 36H, *^t^* Bu). ^13^C­{^1^H} NMR (101 MHz, THF-*d*_8_): δ 140.85 (*i*-aryl), 136.85 (*m*-aryl), 129.15 (*p*-aryl), 127.41 (*o*-aryl), 75.40 (–*C*(CH_3_)_3_), 33.58 (–C­(*C*H_3_)_3_). ^29^Si-DEPT24 (79 MHz, THF-*d*_8_): δ −35.15 (s). ^171^Yb­{^1^H} (70 MHz, THF-*d*_8_): δ 564.44 (s) IR (cm^–1^): *v*. 3067 (vw), 2971 (vw), 1588 (vw), 1468 (vw), 1426 (w), 1392 (vw), 1367 (w), 1233 (m), 1170 (m), 1107 (m), 1029 (vw), 942 (s), 912 (w), 799 (w), 753 (w), 738 (w), 724 (m), 700 (s), 658 (vw), 633 (m), 520 (s), 495 (s), 462 (m), 424 (w). Elem anal. Found (calculated) for C_64_H_76_N_2_O_4_Si_4_Yb: C, 62.51 (62.87); H, 6.38 (6.27); N, 2.27 (2.29).

### Reaction of **2-Yb** with AgBArF_20_

In the glovebox, a 20 mL scintillation vial was charged with **2-Yb** (0.0218 g, 0.018 mmol), Et_2_O (1 mL), and a glass coated magnetic stirring bar. To this vial was added a solution of silver tetrakis­(perfluorophenyl)­borate (AgBArF_20_) (0.0141 g, 0.018 mmol, 1.0 equiv) in Et_2_O (4 mL). Immediately following addition, the formation of a dark solid (presumably Ag^0^) was observed and the solution began to turn a pale yellow. The reaction mixture was stirred for 24 h, during which time more dark solids were formed and the entirety of the yellow **2-Yb** starting material had disappeared. The resulting gray-yellow mixture was filtered through a pipet filter packed with glass fiber and Celite and the dark gray solids washed with minimal Et_2_O. The clear, yellow filtrate was reduced *in vacuo* and the residue triturated with pentane (3 × 1 mL). The residue was then extracted with THF (2 mL), filtered through a pipet filter packed with glass fiber and Celite, concentrated to ∼1 mL total volume, and set up for vapor diffusion of pentane into the concentrated THF solution at −35 °C. The crystals yielded from this reaction were analyzed using single-crystal X-ray diffraction and were found to be consistent with a mixture of **2-Yb** and **1-H**. The crystallization mixture was reduced *in vacuo* and the resulting residue dissolved in THF-*d*_8_ for ^1^H NMR analysis (Figure S37). Resonances in the^1^H spectrum were consistent with a mixture of **2-Yb**, **1-H**, and other diamagnetic impurities.

### Reaction of **2-Yb** with FcBArF_20_

In the glovebox, a 20 mL scintillation vial was charged with ferrocenium tetrakis­(perfluorophenyl)­borate (FcBArF_20_) (0.0408 g, 0.047 mmol) and a glass coated magnetic stirring bar. To this vial was then added a solution of **2-Yb** (0.0567 g, 0.046 mmol, 0.98 equiv) in PhF (3 mL). The dark blue color of the suspended FcBArF_20_ quickly faded and the reaction mixture took on a bright yellow color. After stirring overnight, an aliquot of the mixture was taken and reduced *in vacuo*. The residue was then dissolved in THF-*d*_8_ for ^1^H NMR analysis (Figure S38). Resonances in the^1^H spectrum were consistent with the presence of **1-H**, residual solvent(s) and multiple broad, unassigned features.

### Reaction of [TmI_3_(THF)_3.5_] with KC_8_ and **1-K**

In the glovebox, a 20 mL scintillation vial was charged with [TmI_3_(THF)_3.5_] (0.0655 g, 0.082 mmol), KC_8_ (0.0150 g, 0.111 mmol, 1.36 equiv), DME (3 mL), and a glass coated magnetic stirring bar. This mixture was placed in the cold well (*T* < −60 °C) and stirred for 20 min, during which time the suspended KC_8_ faded from bronze to black and the solution took on a deep emerald-green color. To this mixture was then added a prechilled solution of **1-K** (0.0930 g, 0.165 mmol, 2.02 equiv) in DME (4 mL) and the resulting mixture was then stirred for 1 h in the cold well. The dark colored mixture was then filtered through a prechilled pipet filter packed with glass fiber and Celite into a cold, tared vial to afford a dark, brownish filtrate. The filtrate was concentrated to a total volume of ∼3 mL, layered with an equal volume of pentane, and stored at −35 °C to crystallize. After several weeks, the pentane had completely diffused into the DME, and dark crystals had formed alongside a white precipitate. Single crystals taken from the mixture were identified as [Tm_2_I­(μ-η^2^:η^2^-N_2_)­(N­(Ph_2_SiO*^t^* Bu)_2_)_2_(DME)] (**3-Tm**). After several weeks, additional, visually indistinguishable single crystals were taken from this mixture and identified as [Tm­(N­(Ph_2_SiO*^t^* Bu)_2_)_2_] (**2-Tm**).

## Supplementary Material



## Notes

General experimental considerations, NMR, IR, and UV–vis–NIR spectra, SQUID, electrochemical, and crystallographic data (PDF)

## Abbreviations

DEPT: distortionless enhancement by polarization transfer
HSQC: heteronuclear single quantum coherence
HMBC: heteronuclear multiple bond correlation
