# Supplementary material for: Bis(tert-butoxydiphenylsilyl)amide Divalent Lanthanide Complexes
Source: Inorg Chem. 2025 May 23;64(22):10751–60. doi: 10.1021/acs.inorgchem.5c00277 (PMC12152930; doi:10.1021/acs.inorgchem.5c00277)
Supplement: Supplementary file 1 [file ic5c00277_si_001.pdf]

Supporting Information:

bis(*tert*-Butoxydiphenylsilyl)amide Divalent Lanthanide Complexes

Grant R. Wilkinson,[a] Sarah J. Schultz,[a] Kaitlyn S. Otte,[a] Maximilian G. Bernbeck,[a] and Henry S. La Pierre[a,b]*

[a] School of Chemistry and Biochemistry, Georgia Institute of Technology, Atlanta, GA 30332-0400, United States

[b] Nuclear and Radiological Engineering Program, Georgia Institute of Technology, Atlanta, GA 30332-0400, United States

* *hsl@gatech.edu*

Contents

General Considerations S2
NMR Spectra S4
IR Spectra S24
Electronic Absorption Spectroscopy S27
Cyclic Voltammetry S30
Crystallography S33
DC Magnetometry S43
References S46

General Considerations

Unless otherwise noted, all reagents were obtained from commercial suppliers and used without further purification. The syntheses and manipulations described herein were conducted under argon or nitrogen with exclusion of oxygen and water using Schlenk techniques or in an inert atmosphere box (Vigor) under a dinitrogen (<0.1 ppm O_2/H_2O) atmosphere unless otherwise noted. The glovebox is equipped with a -35 ºC freezer and a cold well. All glassware and cannulae were stored in an oven overnight (>8 h) at a temperature of ca. 160 ºC. Celite and molecular sieves were dried under vacuum at a temperature >250 ºC for a minimum of 24 h. C_6D_6 (CIL) was stored over 3 Å molecular and then vacuum-transferred from purple sodium/benzophenone and stored over fresh sieves prior to use. THF-d_8 was degassed by three freeze-pump-thaw cycles, dried over fresh sodium metal for 7 days, vacuum-transferred, and stored over 3 Å molecular sieves prior to use. Pentane, hexanes, diethyl ether (Et_2O), tetrahydrofuran (THF), 1,2-dimethoxyethane (DME), and toluene were sparged with UHP-grade argon (Airgas) and passed through columns containing Q-5 and molecular sieves in a solvent purification system (JC Meyer Solvent Systems). All solvents in the glovebox were stored in bottles over 3 Å molecular sieves. Fluorobenzene (PhF) was refluxed over CaH_2 overnight, distilled, and stored in the glovebox in a media bottle over 3 Å molecular sieves. *Tert*-butanol was refluxed over CaH_2 overnight, distilled, and stored in a Strauss flask under inert atmosphere. Deionized water was purified using a Milli-Q system (Millipore) and degassed by two freeze-pump-thaw cycles. Potassium *tert*-butoxide was sublimed prior to use in synthesis of potassium benzyl. Lithium diisopropylamide (LDA),[1] potassium benzyl (KBn),[2] *tert*-butoxychlorodiphenylsilane (Ph_2SiO^tBuCl),[3] [$LnI_2(THF)_2$] (Ln=Sm, Eu, Yb),[4] [$TmI_3(THF)_{3.5}$],[5] potassium graphite (KC_8),[6] and silver tetrakis(perfluorophenyl)borate ($AgBArF_{20}$)[7] were synthesized following literature procedures. Ferrocenium tetrakis(perfluorophenyl)borate ($FcBArF_{20}$) was also prepared following a modified literature procedure substituting [K][$B(C_6F_5)_4$] for [$Li(Et_2O)_2$][$B(C_6F_5)_4$] and $Fe(NO_3)_3(H_2O)_4$ for $FeCl_3$.[8] Cargile Type NVH immersion oil was degassed on a Schlenk line by stirring under active vacuum with gentle heating (to facilitate stirring, ca. 40 ºC) overnight prior to use. Tetrabutylammonium hexafluorophosphate ([$^nBu_4N$][PF_6]) (Oakwood) was recrystallized three times from absolute ethanol and dried under vacuum at 85 ºC for 24 h prior to use. Elemental analysis was performed at the University of Iowa MATFab Facility and presented C, H, and N values are given as the average of duplicate analyses.

NMR spectra were obtained on a Bruker Advance III 400 MHz at 298 K unless otherwise noted and are processed and presented using MestReNova® v14.1.0-24037. ^1H and $^{13}C\{^1H\}$ NMR chemical shifts are reported in δ, parts per million, and are referenced to the ^1H/^{13}C resonance(s) of the deuterosolvent. ^{29}Si-DEPT24 spectra are referenced to the absolute frequency of the ^1H resonance(s) of the deuterosolvent using MestReNova and chemical shifts are reported in δ, parts per million. $^{171}Yb\{^1H\}$ chemical shifts are also reported in δ, parts per million, and referenced using the absolute frequency of $\delta_{^1H} = 0$ ppm for of the corresponding referenced ^1H spectrum following the recommended IUPAC methodology ($\Xi_{^{171}Yb}$ = 17.499306% for [$Cp^*_2Yb(THF)_2$] (Cp^{*-} = 1,2,3,4,5-pentamethylcyclopentadienyl))[9] and applying a linear offset of $\delta_{^{171}Yb} =$ -0.31 ppm to account for the use of THF-d_8 as solvent.[10] Peak position is listed, followed by peak multiplicity, integration value, and proton or carbon assignment where applicable. Multiplicity and shape are indicated by the following abbreviations: s (singlet); d (doublet); t (triplet); q (quartet); dd (doublet of doublets); td (triplet of doublets); m (multiplet); b (broad). Solvent and other common were identified by comparison to known impurity signals.[11] Infrared (IR) spectra of the pure compounds were recorded on a Bruker ALPHA FTIR spectrometer from 400 to 4000 cm^{-1} using attenuated total reflection (ATR) and peaks are listed in wavenumber [cm^{-1}] and intensity is indicated by the following abbreviations: vw (very weak); w (weak); m (medium); s (strong); vs (very strong); br (broad). UV-Vis-NIR spectra were recorded from 250 to 1500 nm using a Hitachi UH4150 scanning spectrophotometer and small-volume screw cap quartz cuvettes (Starna Scientific) with a 1 cm path length.

Crystals suitable for single-crystal x-ray diffraction were immersed in NVH oil and transferred to the diffractometer in sealed 20 mL scintillation vials. The data for all structures were collected from a shock-cooled single crystal at 100(2) K on a Bruker D8 VENTURE dual wavelength Mo/Cu three-circle diffractometer with a microfocus sealed x-ray tube using a mirror optics as monochromator and a Bruker PHOTON III detector. The diffractometer was equipped with an Oxford Cryostream 800 low-temperature device and used Mo-K_α radiation (λ = 0.71073 Å) for the reported collections. Information on structural solutions and refinement details for each dataset collected are described in the Crystallography section. Crystallographic data for the structures reported in this paper have been deposited with the Cambridge Crystallographic Data Centre.[12] CCDC 2415057 and 2411055-2411061 contain the supplementary crystallographic data for this paper. These data can be obtained free of charge from the Cambridge Crystallographic Data Centre via www.ccdc.cam.ac.uk/structures. Crystallographic reports and CIF files were generated using FinalCIF.[13-15] Structures were visualized and analyzed in Mercury (CCDC) and graphics were generated using POV-Ray.[16, 17] Continuous shape measure (CShM) analysis was performed using the SHAPE2.1 program.[18]

Quartz wool was used to immobilize samples for DC magnetometry measurements and was cleaned following a modification of a published procedure.[19] Quartz wool (Technical Glass) was leached with a mixture of oxalic acid (0.1 M) and sulfuric acid (0.5 M) in deionized water in a covered glass beaker for 3 h at 90 ºC with occasional agitation using a glass stirring rod. The quartz wool was then collected on a glass frit, washed with deionized water (1 L), and dried *in vacuo* overnight (> 8 h). The wool was further dried in an oven set to 160 ºC overnight (> 8 h) prior to being brought into the glovebox for use.

NMR Spectra

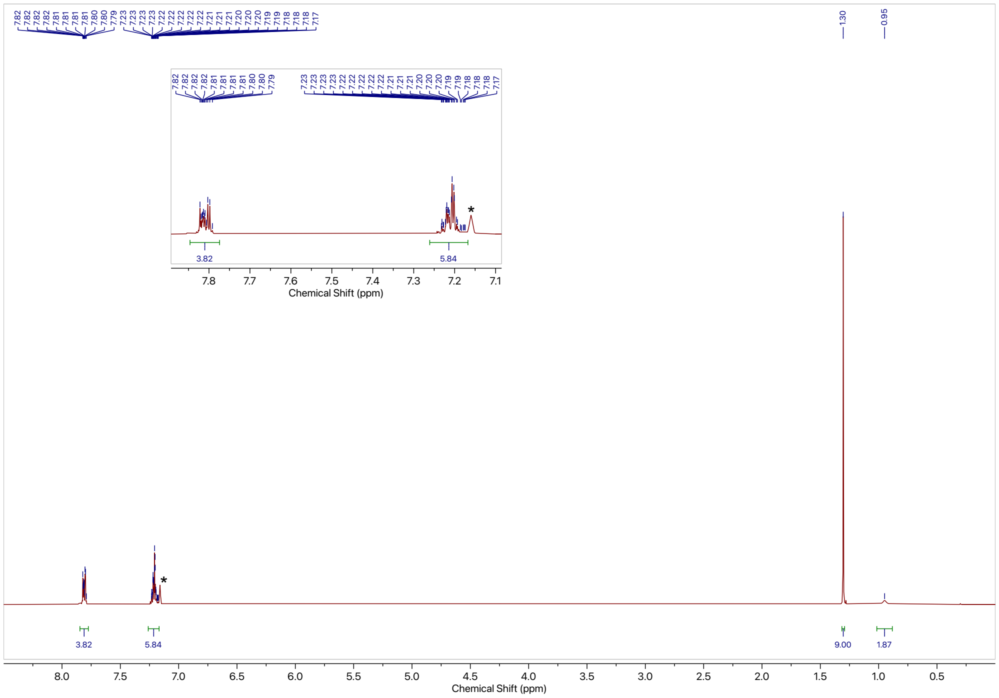

Figure S1. ^{1}H spectrum of $Ph_2SiO^tBuNH_2$ in C_6D_6. Residual C_6D_5H is denoted as *.

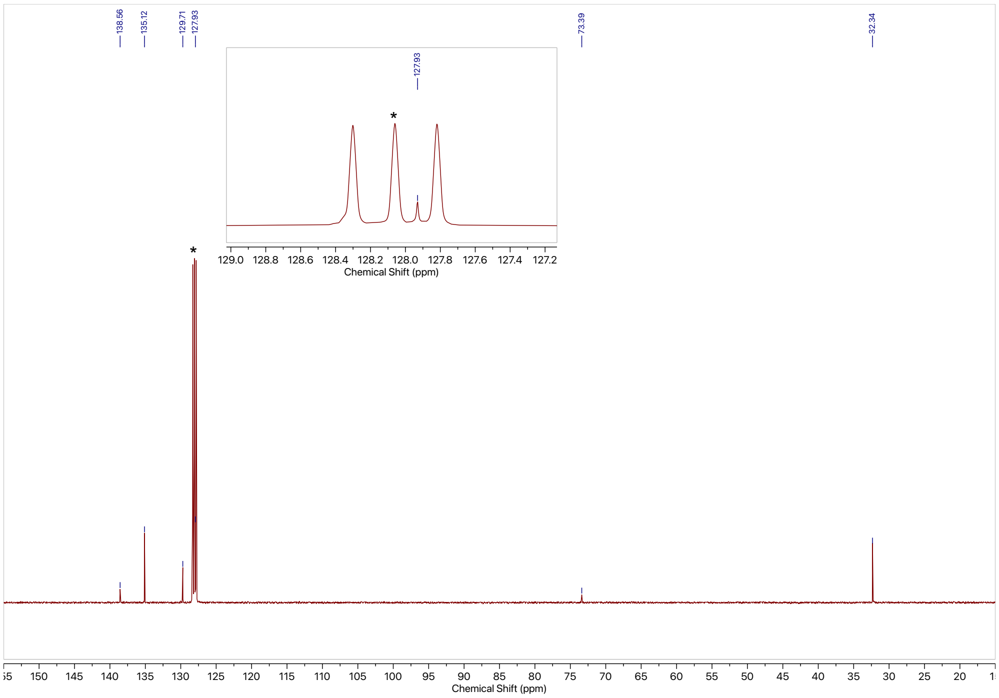

Figure S2. $^{13}C\{^{1}H\}$ spectrum of $Ph_2SiO^tBuNH_2$ in C_6D_6. Residual C_6D_5H is denoted as *.

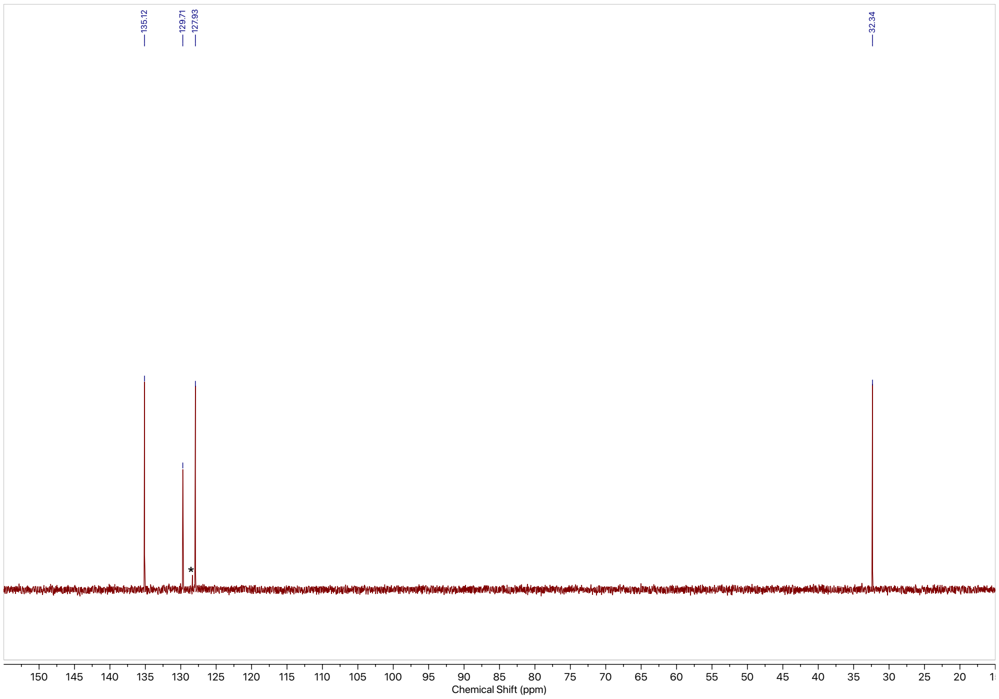

Figure S3. ^{13}C-DEPT135 spectrum of $Ph_2SiO^tBuNH_2$ in C_6D_6. Residual starting material peak(s) (Ph_2SiO^tBuCl) are denoted as *.

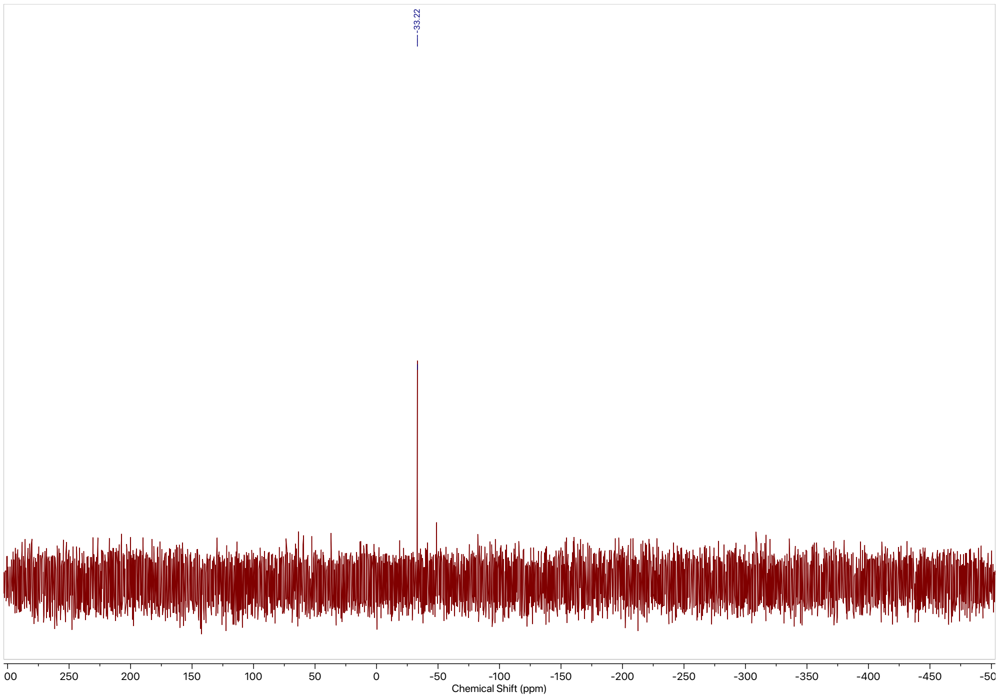

Figure S4. ^{29}Si-DEPT24 spectrum of $Ph_2SiO^tBuNH_2$ in C_6D_6.

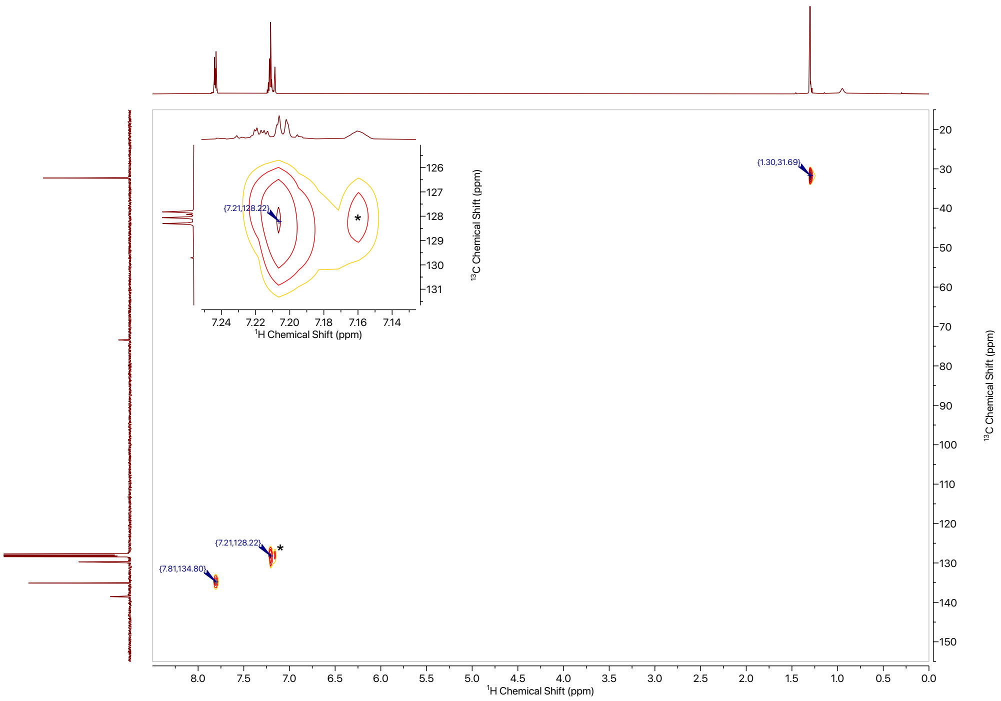

Figure S5. ^{1}H-^{13}C HSQC spectrum of $Ph_2SiO^tBuNH_2$ in C_6D_6. Residual C_6D_5H is denoted as *.

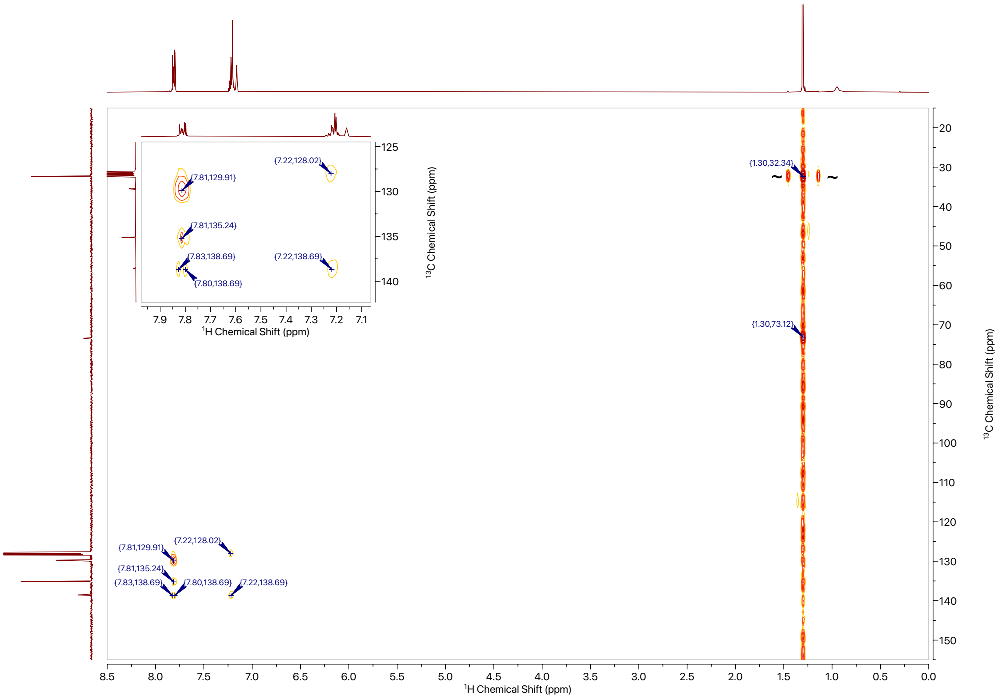

Figure S6. ^{1}H-^{13}C HMBC spectrum of $Ph_2SiO^tBuNH_2$ in C_6D_6. Self-correlation peaks are denoted as ~.

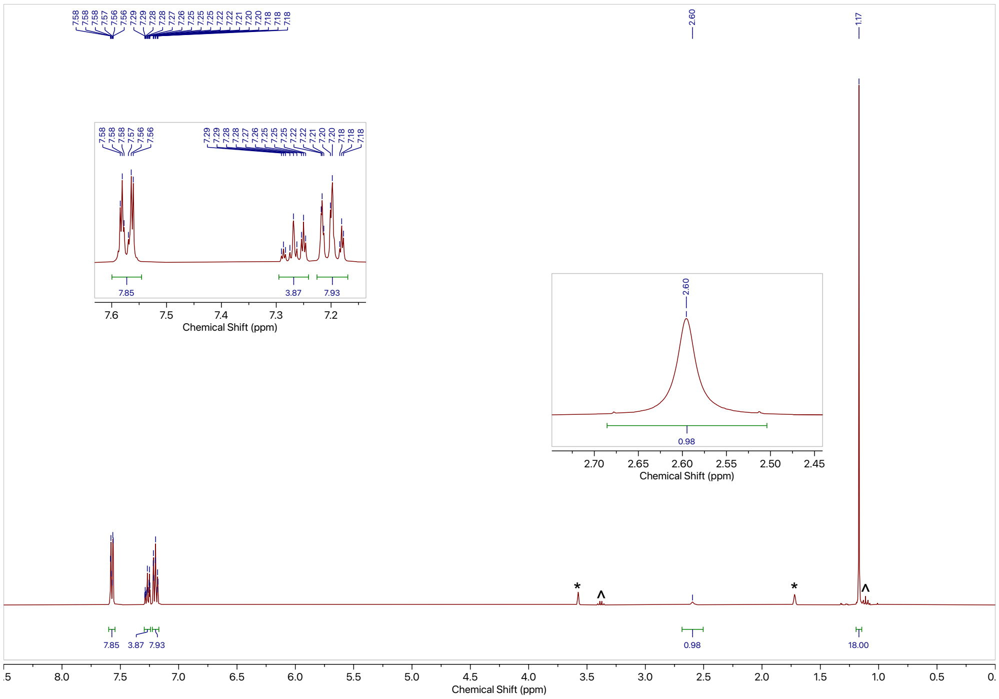

Figure S7. ^{1}H spectrum of **1-H** in THF-d_8. Residual C_4D_7HO and Et_2O are denoted as * and ^ respectively.

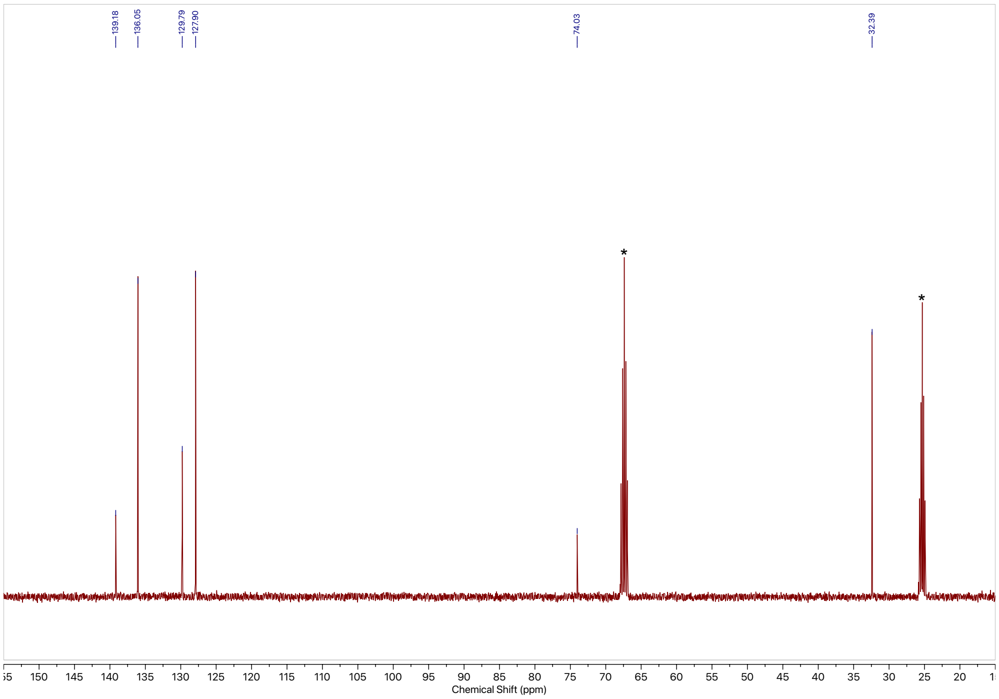

Figure S8. $^{13}C\{^{1}H\}$ spectrum of **1-H** in THF-d_8. Residual C_4D_7HO is denoted as *.

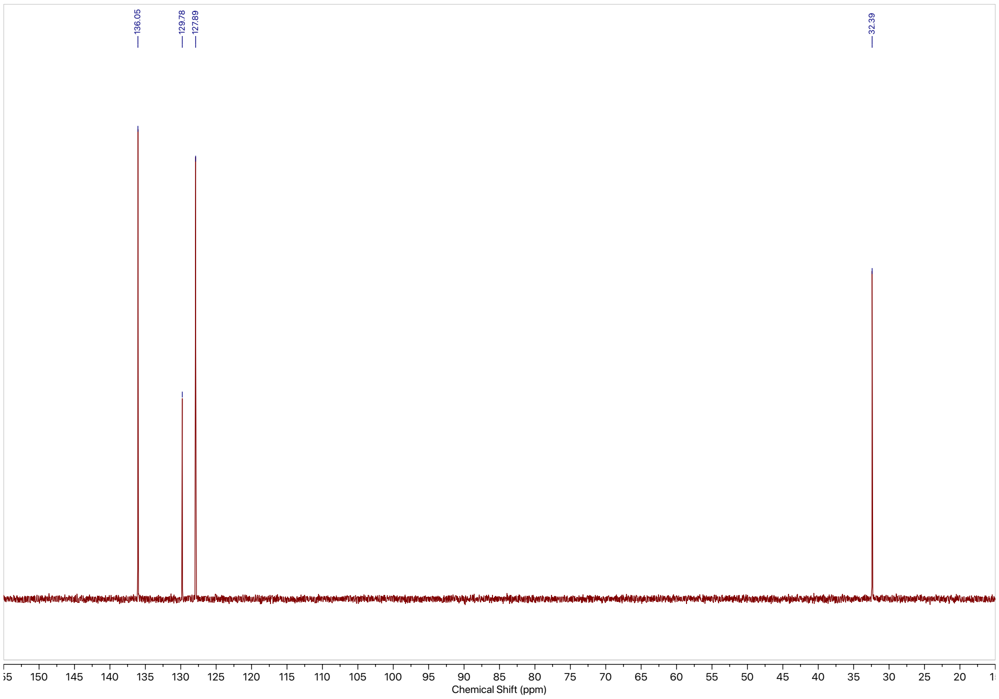

Figure S9. ^{13}C-DEPT135 spectrum of **1-H** in THF-d_8.

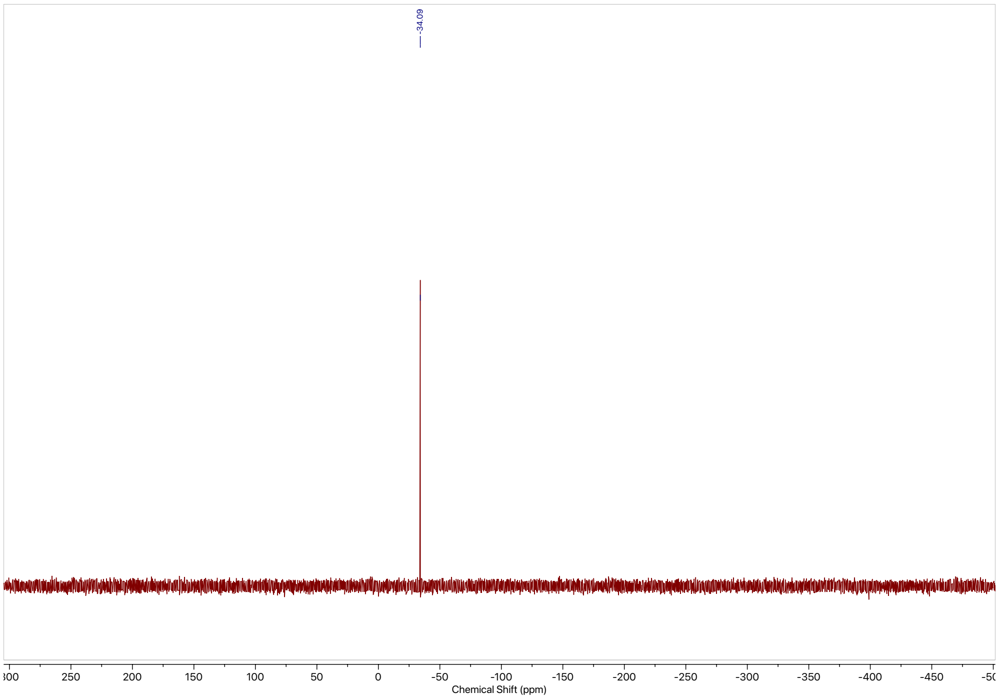

Figure S10. ^{29}Si-DEPT24 spectrum of **1-H** in THF-d_8.

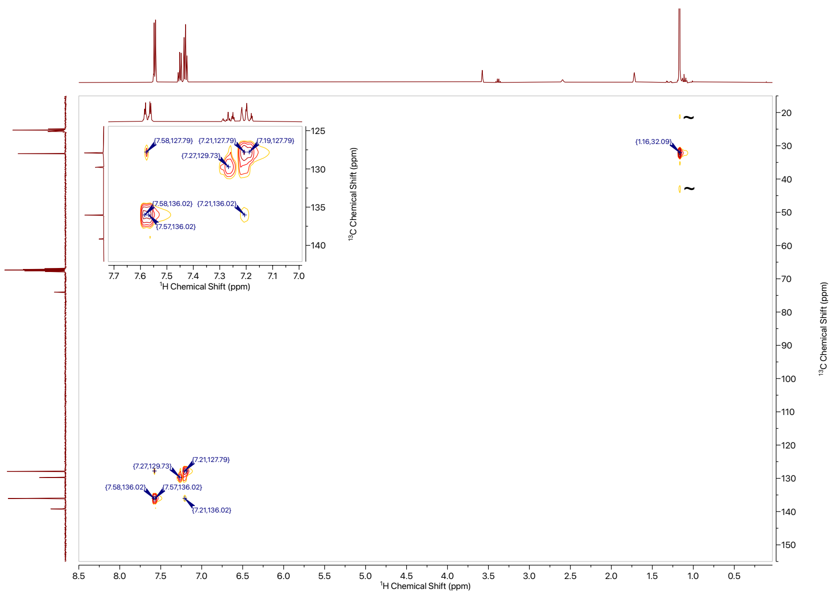

Figure S11. ^{1}H-^{13}C HSQC spectrum of **1-H** in THF-d$_{8}$. Satellite peaks are denoted as ~.

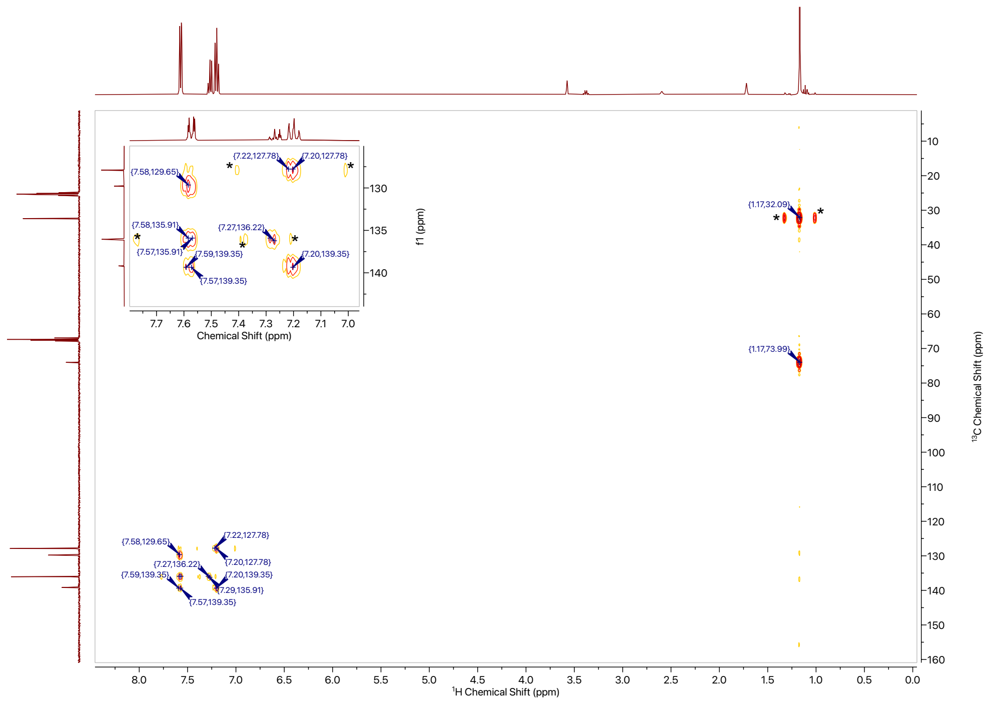

Figure S12. ^{1}H-^{13}C HMBC spectrum of **1-H** in THF-d$_{8}$. Self-correlation peaks are denoted as *.

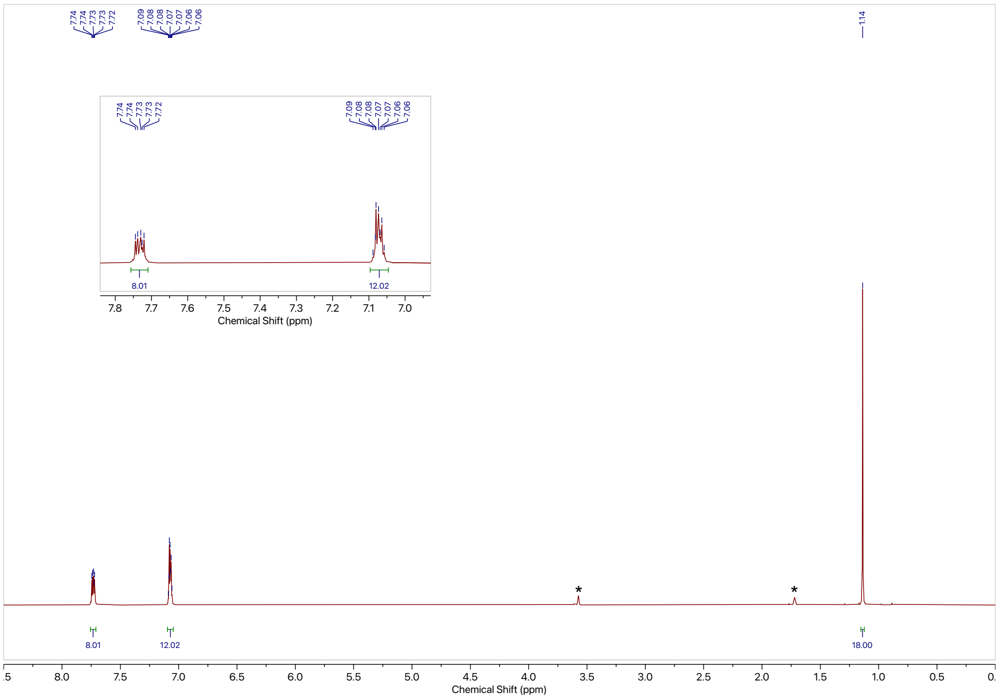

Figure S13. ^1H spectrum of **1-K** in THF-d_8. Residual C_4D_7HO is denoted as *.

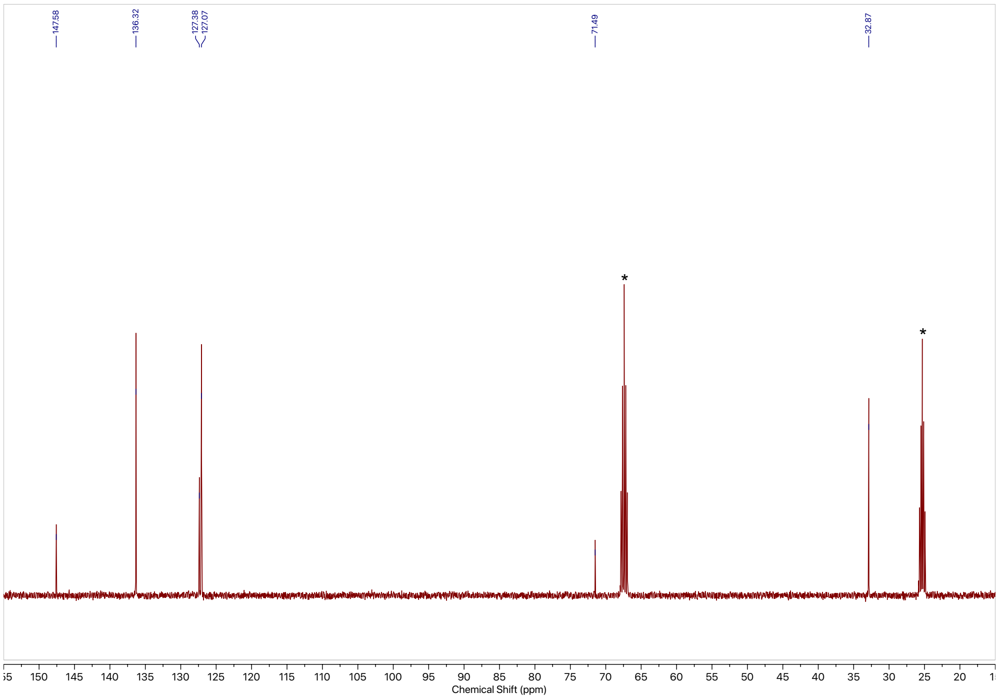

Figure S14. $^{13}C\{^1H\}$ spectrum of **1-K** in THF-d_8. Residual C_4D_7HO is denoted as *.

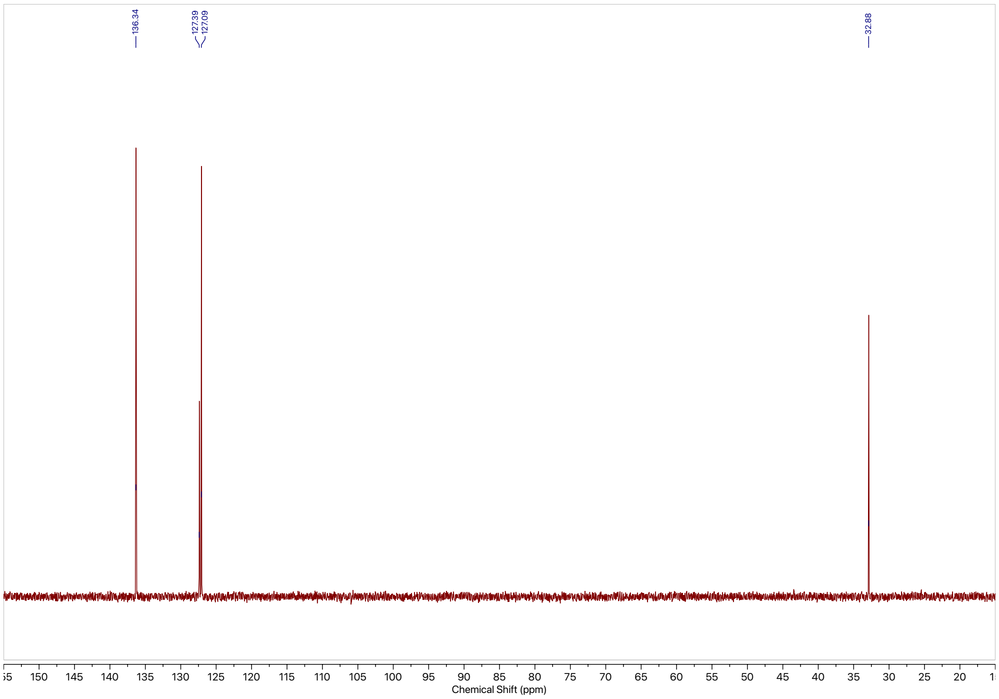

Figure S15. ^{13}C-DEPT135 spectrum of **1-K** in THF-d_8.

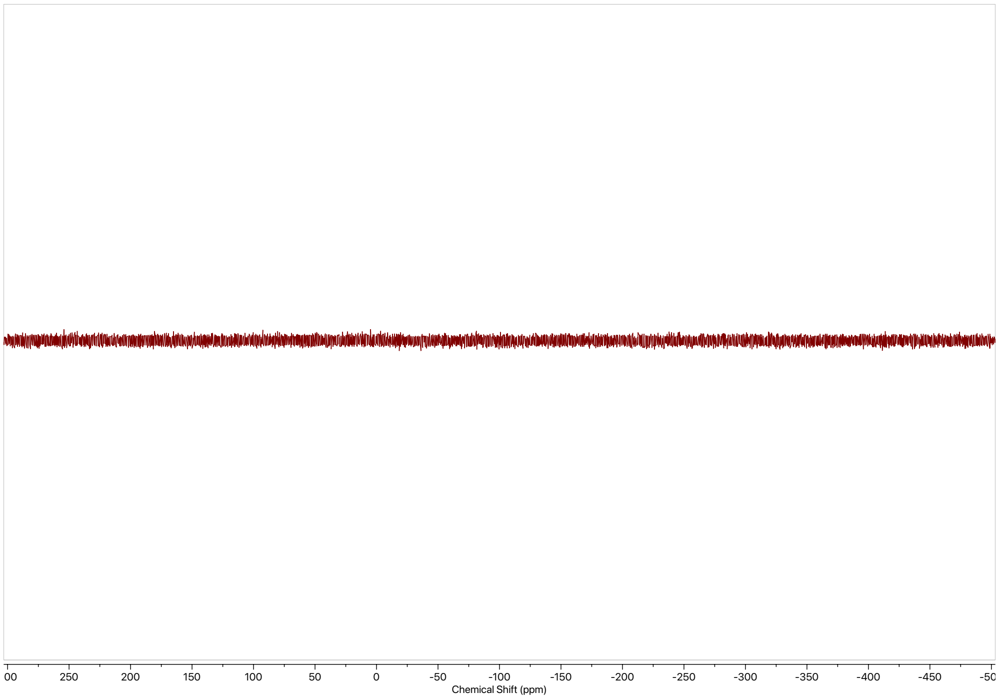

Figure S16. ^{29}Si-DEPT24 spectrum of **1-K** in THF-d_8 in the window 300 ppm to -500 ppm demonstrating no features corresponding to the expected resonance.

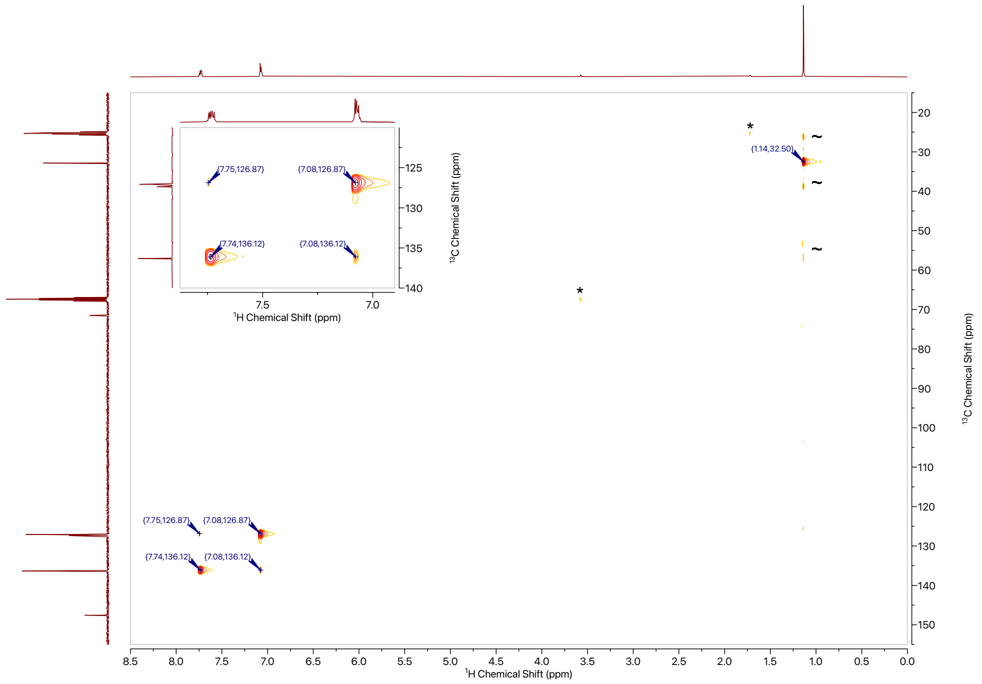

Figure S17. ^{1}H-^{13}C HSQC spectrum of **1-K** in THF-d_8. Residual C_4D_7HO and satellite peaks are denoted as * and ~ respectively.

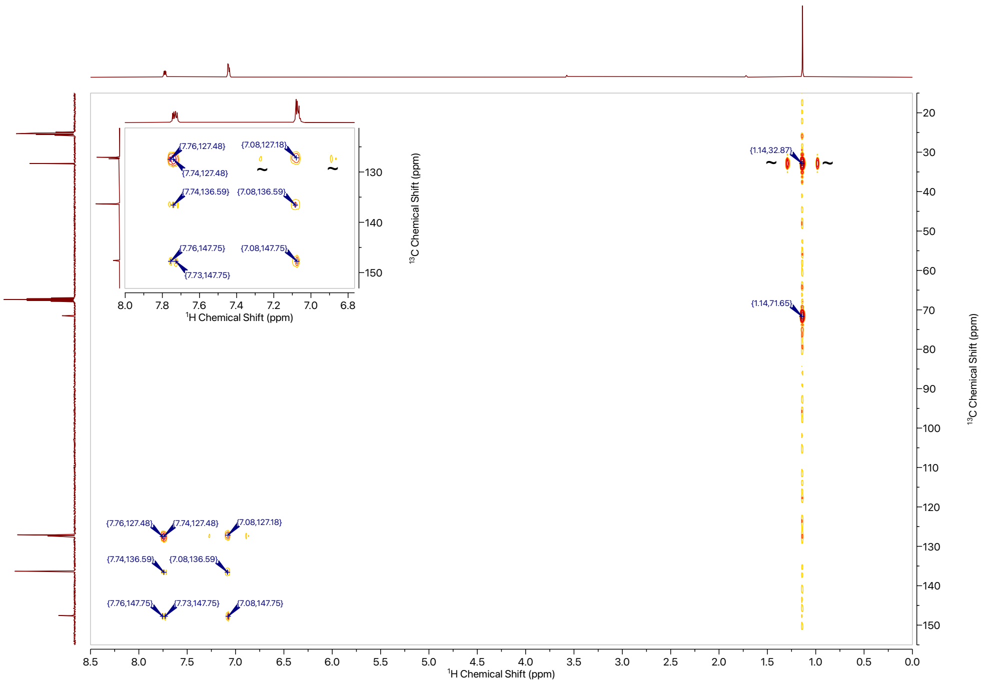

Figure S18. ^{1}H-^{13}C HMBC spectrum of **1-K** in THF-d_8. Self-correlation peaks are denoted as ~.

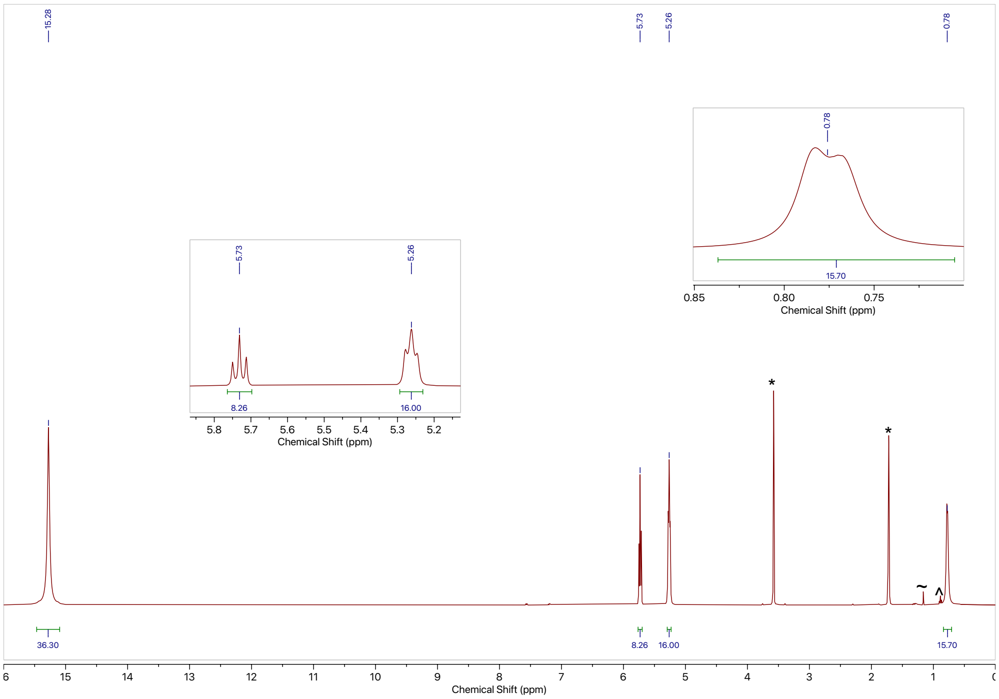

Figure S19. ^{1}H spectrum of **2-Sm** in THF-d_8. Residual C_4D_7HO and pentane are denoted as * and ^ respectively. Trace **1-H** impurity denoted as ~.

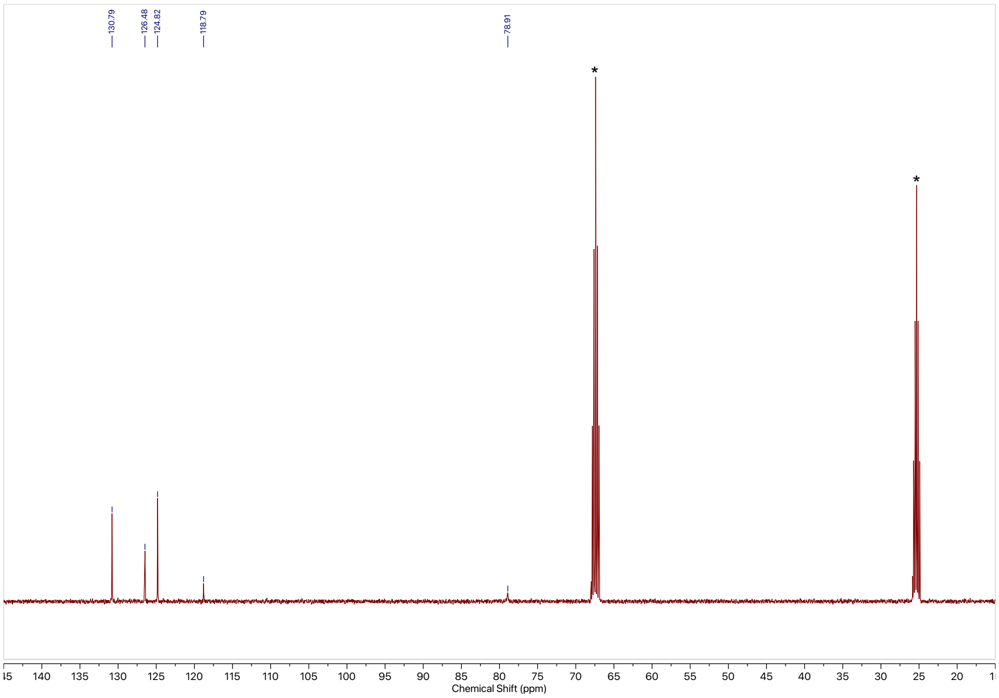

Figure S20. $^{13}C\{^{1}H\}$ spectrum of **2-Sm** in THF-d_8. Residual C_4D_7HO is denoted as *.

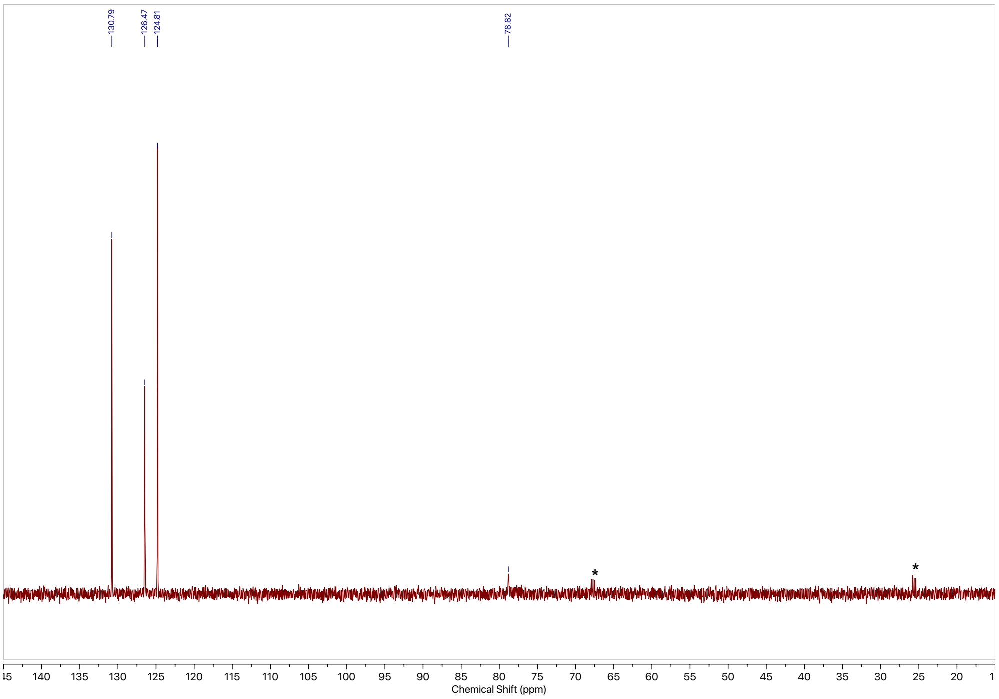

Figure S21. ^{13}C-DEPT135 spectrum of **2-Sm** in THF-d_8. Residual THF is denoted as *.

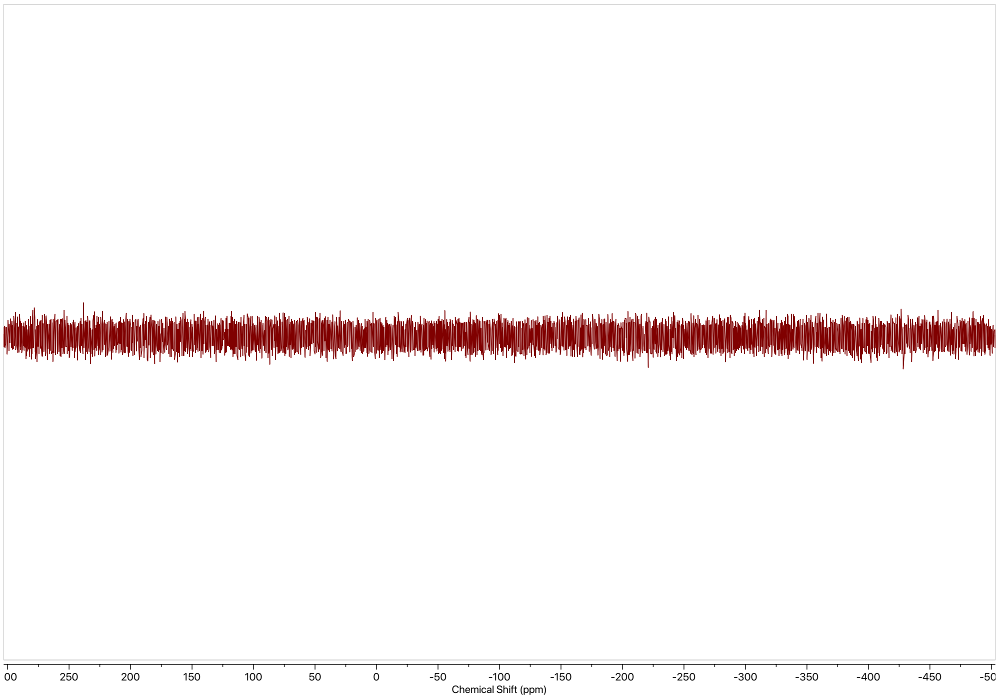

Figure S22. ^{29}Si-DEPT24 spectrum of **2-Sm** in THF-d_8 in the window 300 ppm to -500 ppm demonstrating no features corresponding to the expected resonance.

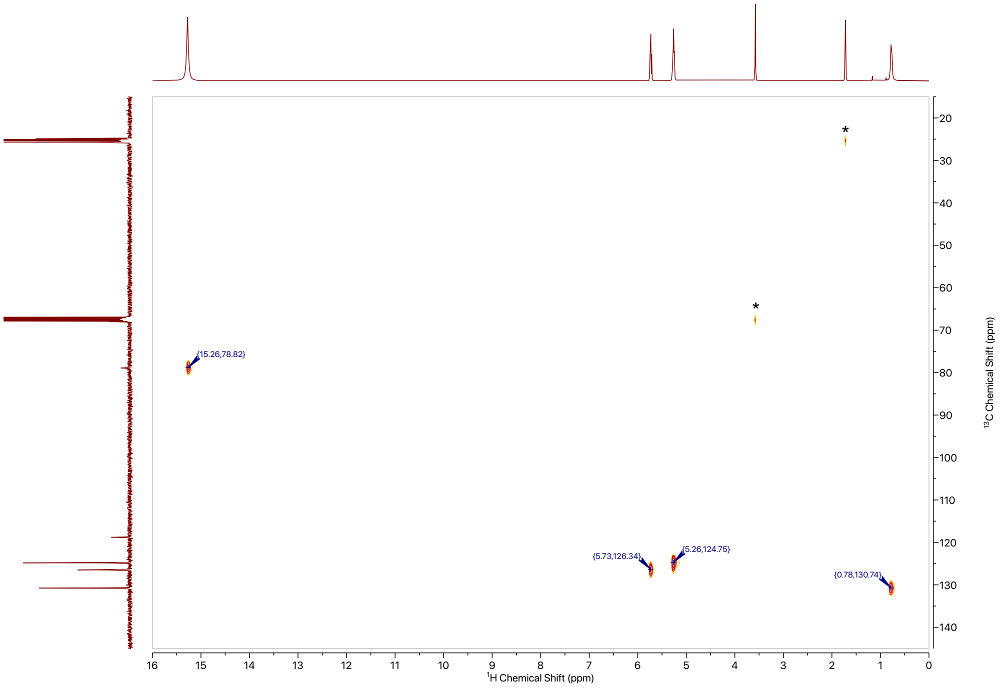

Figure S23. ^{1}H-^{13}C HSQC spectrum of **2-Sm** in THF-d_8. Residual C_4D_7HO is denoted as *.

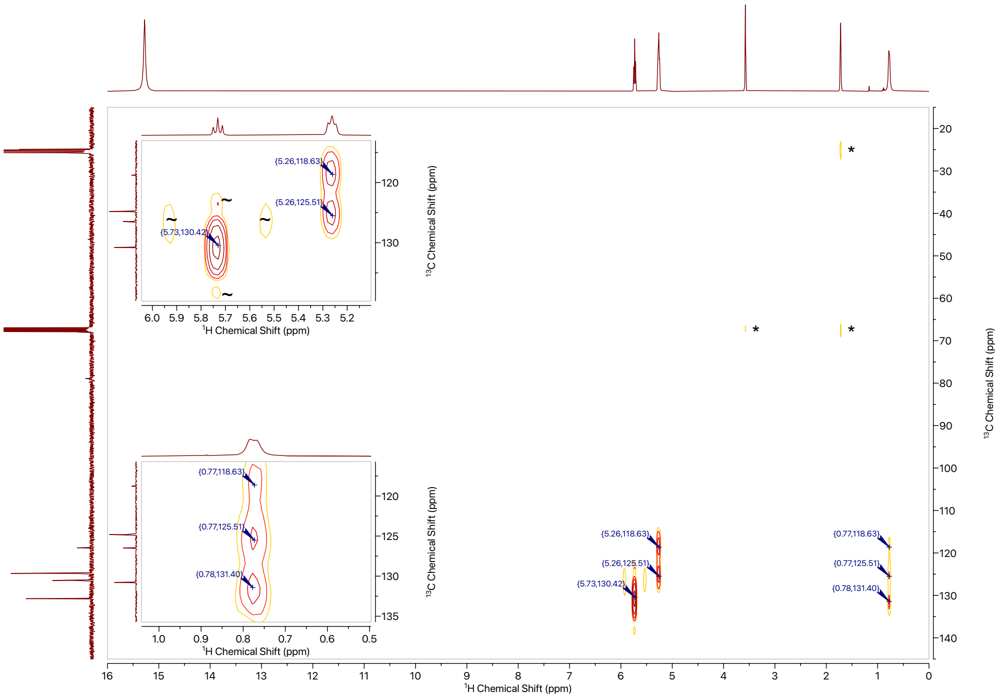

Figure S24. ^{1}H-^{13}C HMBC spectrum of **2-Sm** in THF-d_8. Residual C_4D_7HO and self-correlation peaks are denoted as * and ~ respectively.

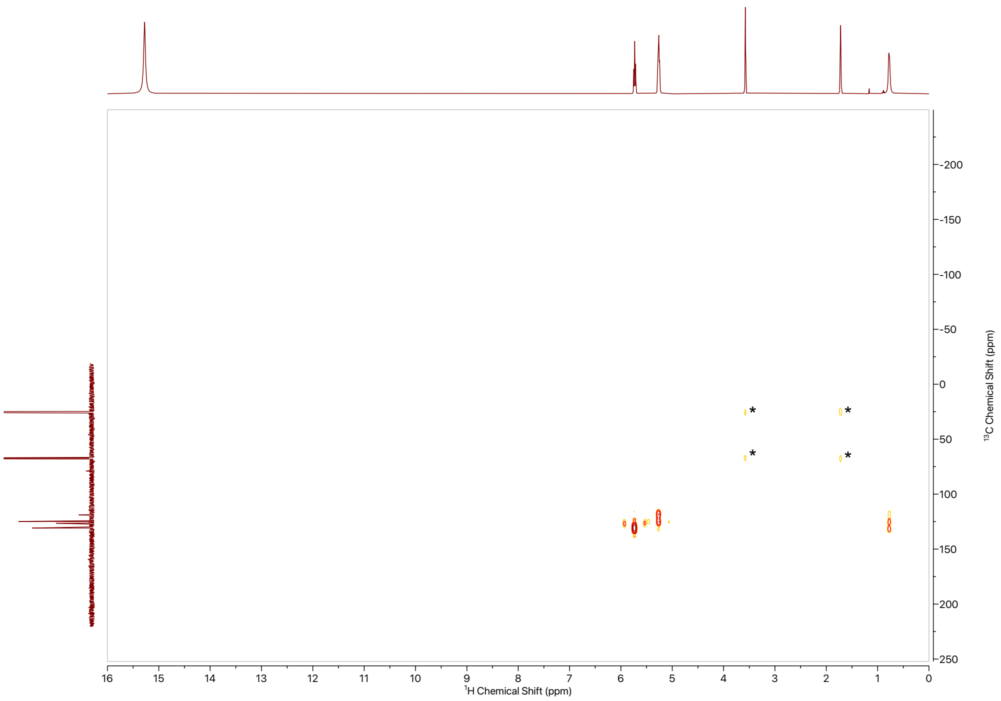

Figure S25. ^1H-^{13}C HMBC spectrum of **2-Sm** in THF-d_8 in the window ± 250 ppm for ^{13}C demonstrating no features corresponding to the expected -*C*(CH_3)$_3$ resonance. Residual C_4D_7HO is denoted as *.

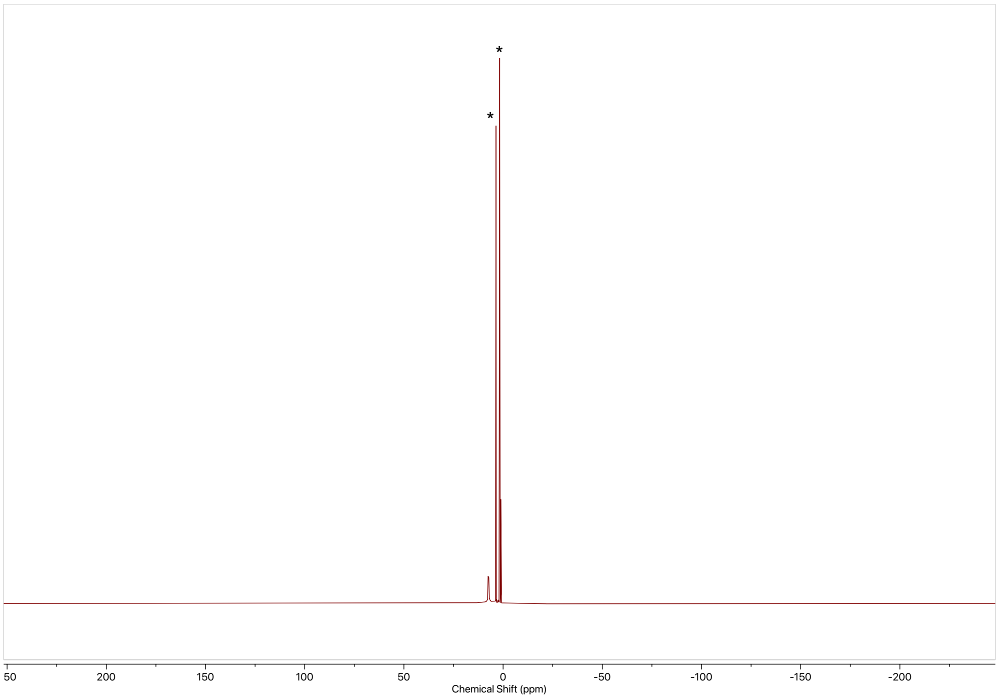

Figure S26. ^1H spectrum of **2-Eu** in THF-d_8 (± 250 ppm) demonstrating no observable features outside the window 0-8.5 ppm. Residual C_4D_7HO is denoted as *.

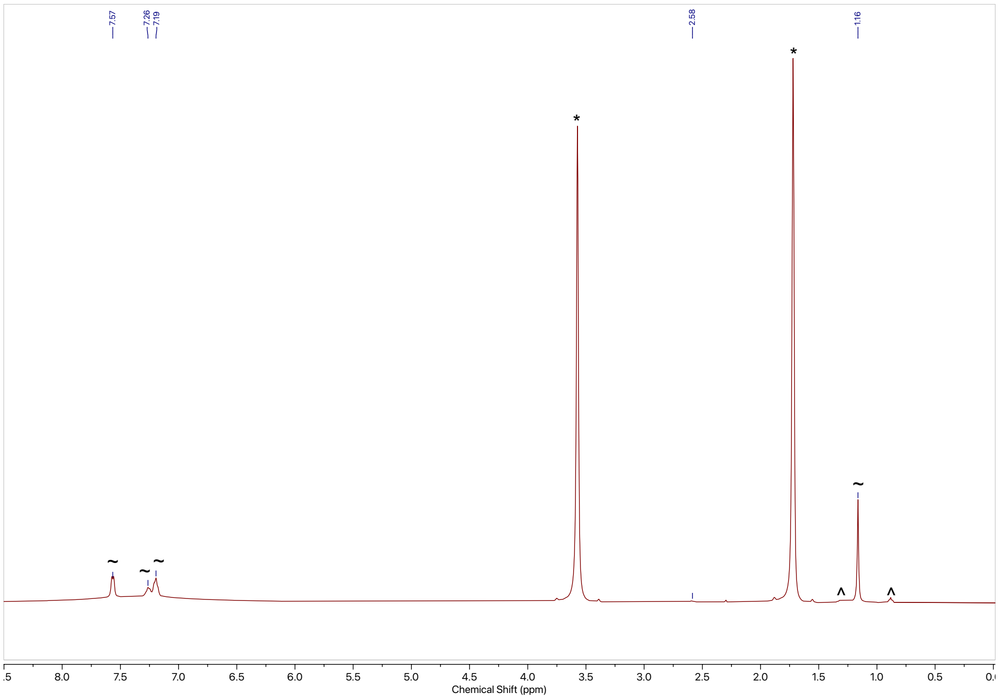

Figure S27. ^{1}H spectrum of **2-Eu** in THF-d_8 (0-8.5 ppm). Residual C_4D_7HO and pentane are denoted as * and ^ respectively. Trace **1-H** impurity is denoted as ~.

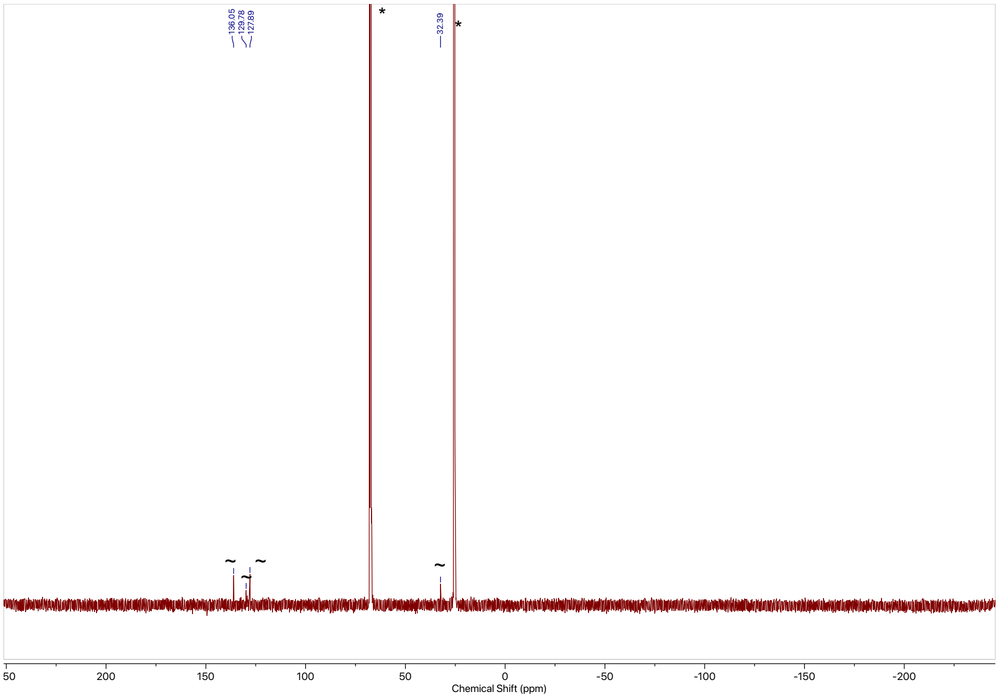

Figure S28. $^{13}C\{^{1}H\}$ spectrum of **2-Eu** in THF-d_8 (± 250 ppm) demonstrating no observable features outside the window 0-150 ppm. Residual C_4D_7HO and trace **1-H** impurity are denoted as * and ~ respectively.

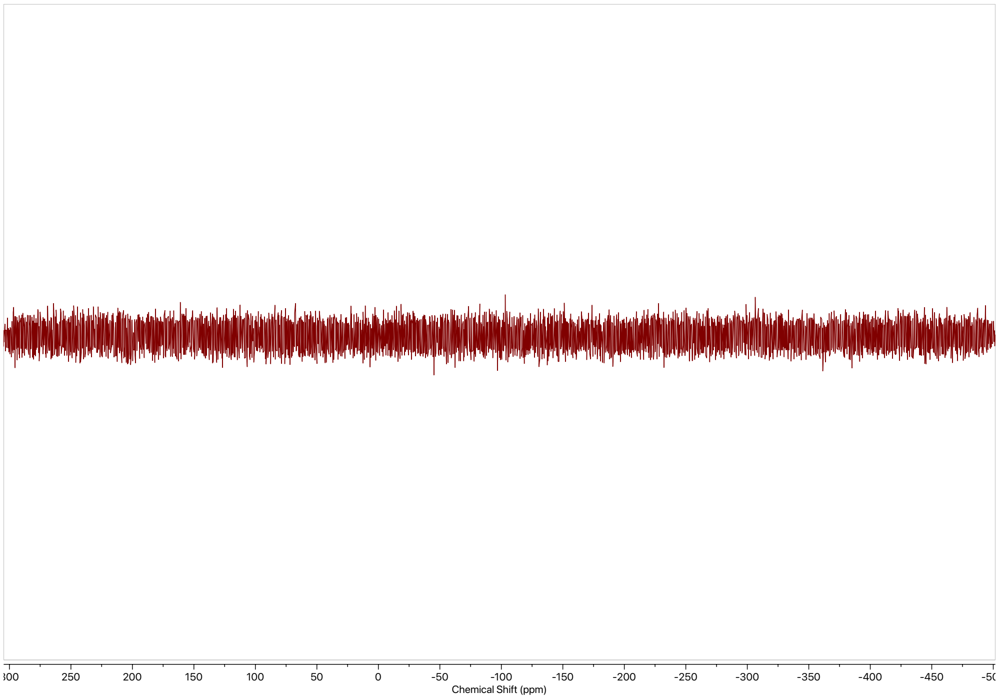

Figure 29. ^{29}Si-DEPT24 spectrum of **2-Eu** in THF-d_8 in the window 300 ppm to -500 ppm demonstrating no features corresponding to the expected resonance.

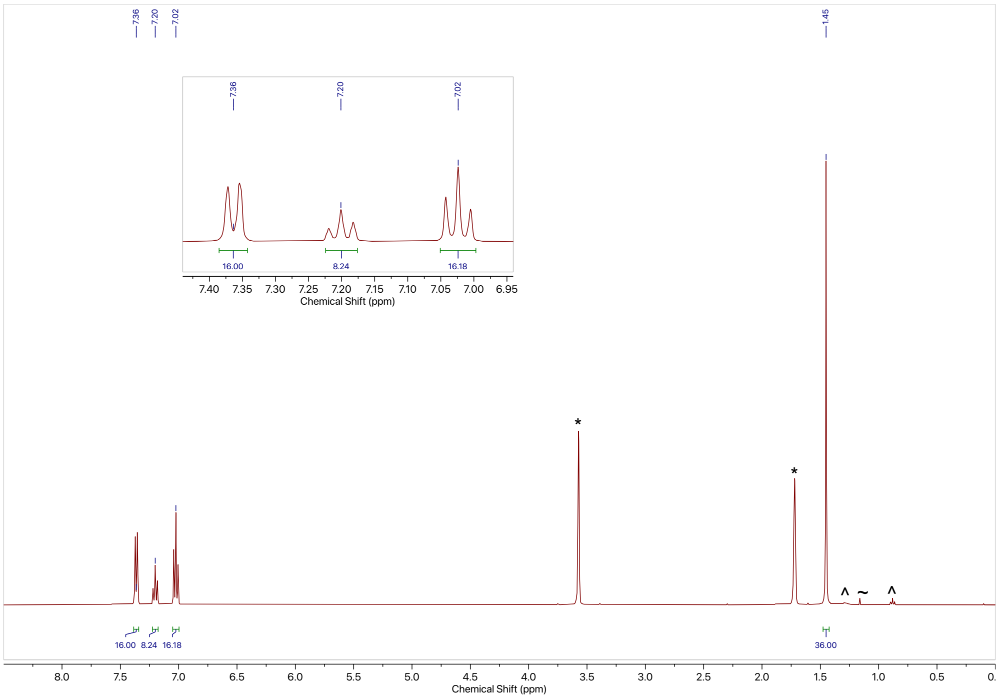

Figure S30. ^{1}H spectrum of **2-Yb** in THF-d_8. Residual C_4D_7HO and pentane are denoted as * and ^ respectively. Trace **1-H** impurity is denoted at ~.

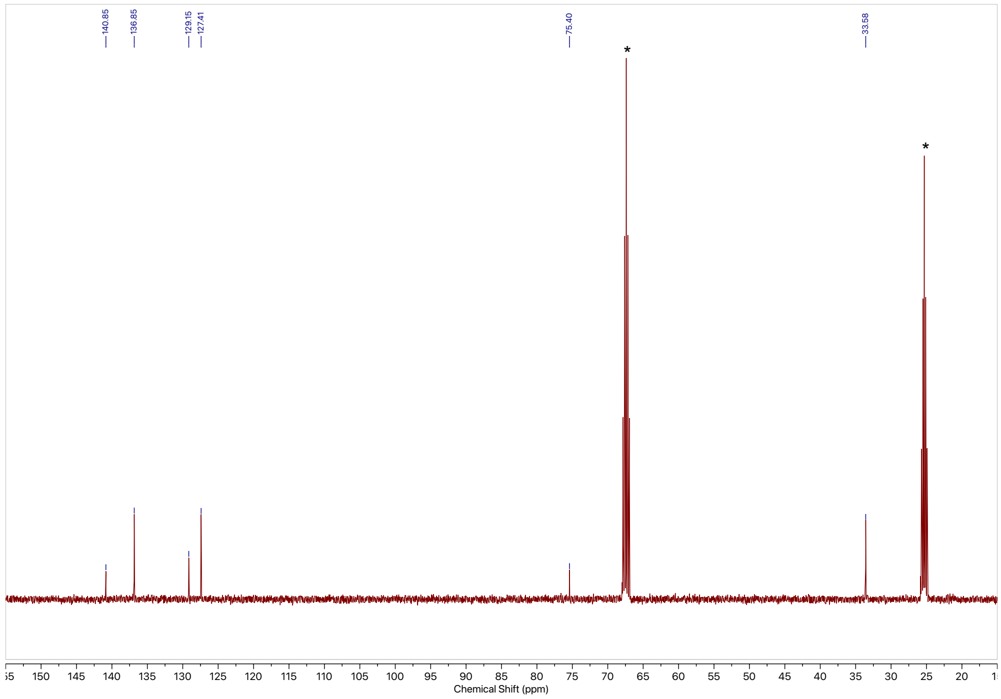

Figure S31. $^{13}C\{^{1}H\}$ spectrum of **2-Yb** in THF-d$_8$. Residual C_4D_7HO is denoted as *.

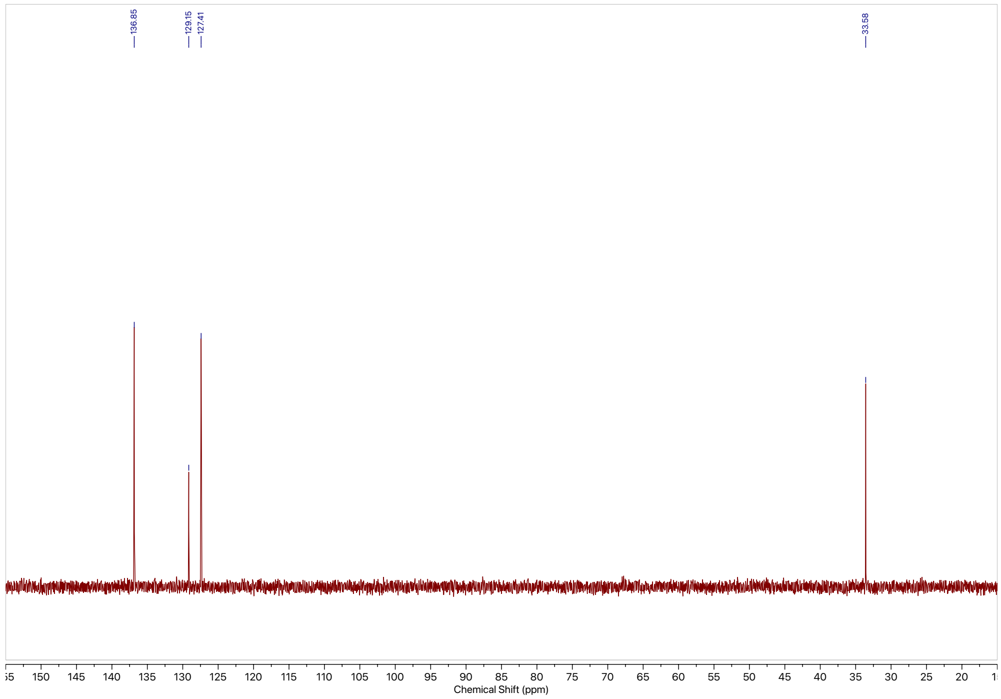

Figure S32. ^{13}C-DEPT135 spectrum of **2-Yb** in THF-d$_8$.

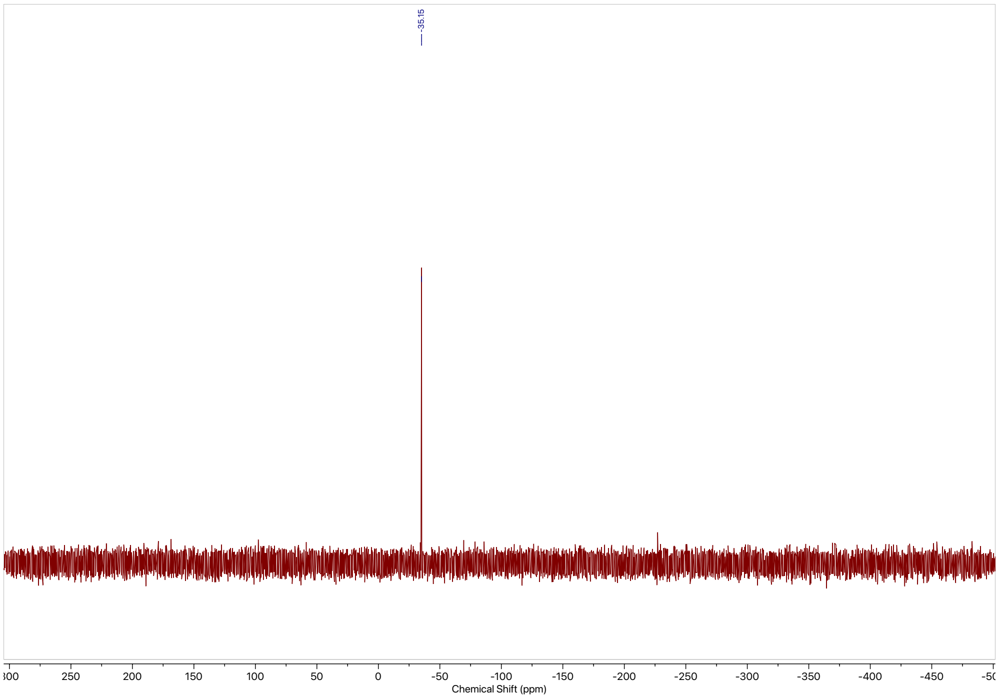

Figure S33. ^{29}Si-DEPT24 spectrum of **2-Yb** in THF-d_8.

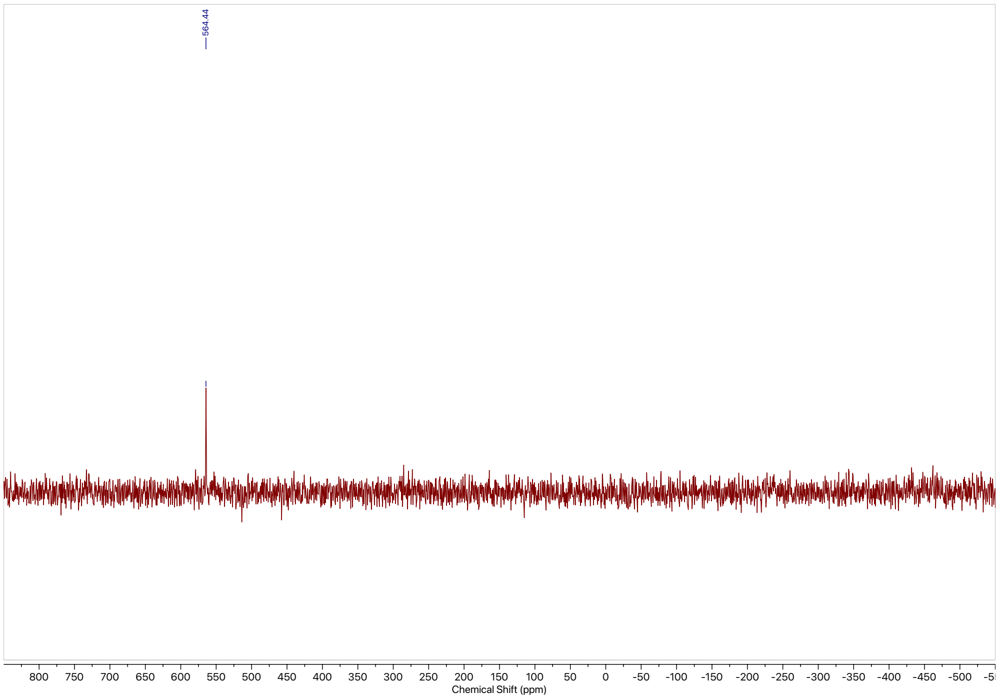

Figure S34. ^{171}Yb{^{1}H} spectrum of **2-Yb** in THF-d_8.

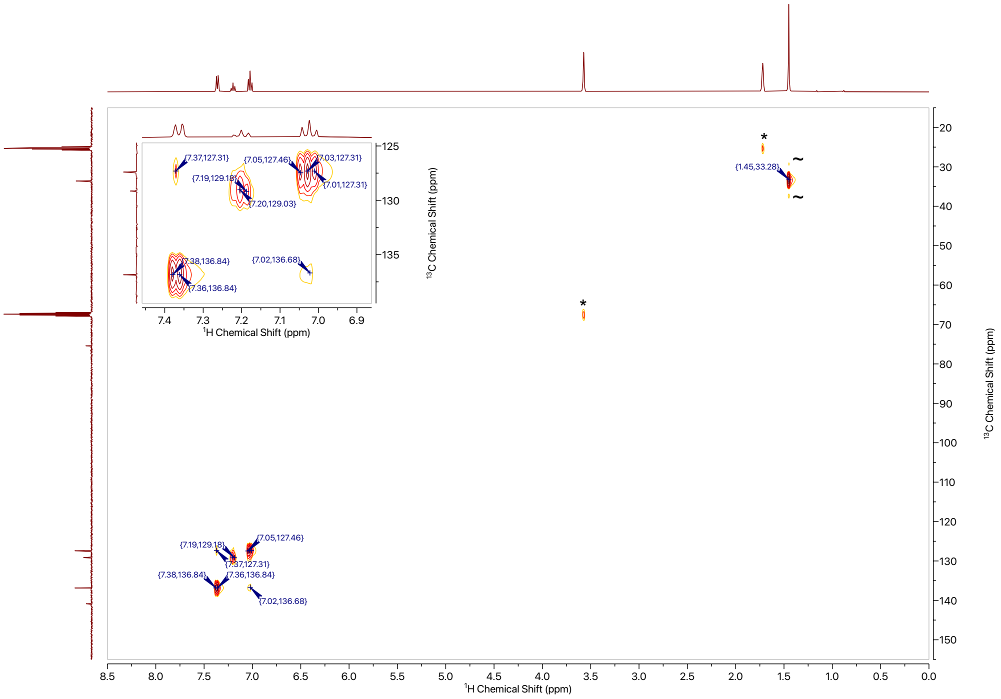

Figure S35. ^{1}H-^{13}C HSQC spectrum of **2-Yb** in THF-d_8. Residual C_4D_7HO and satellite peaks are denoted as * and ~ respectively.

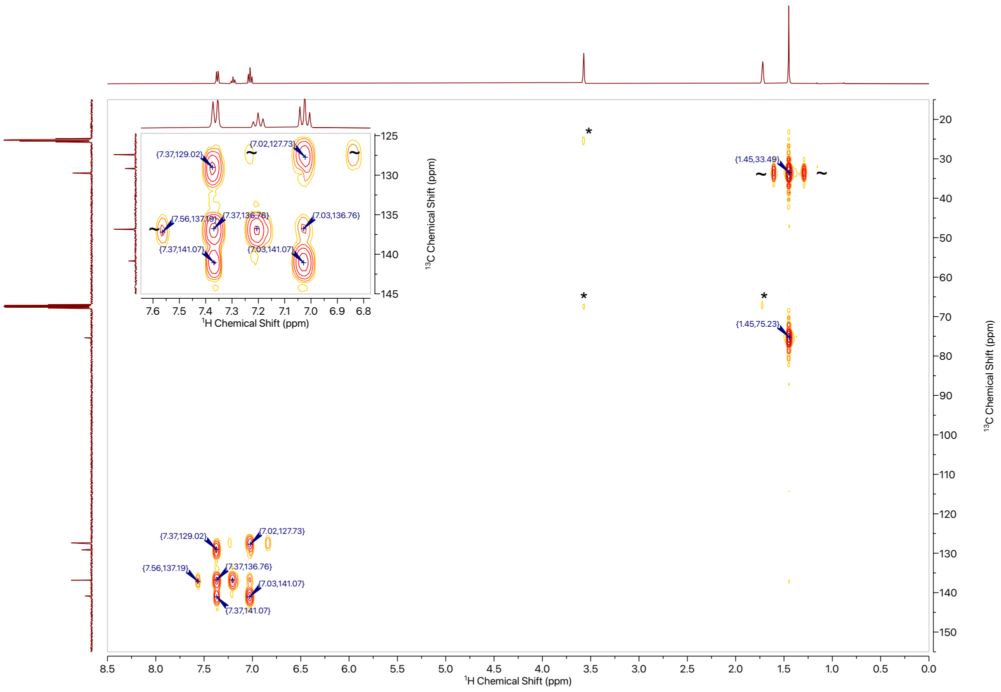

Figure S36. ^{1}H-^{13}C HMBC spectrum of **2-Yb** in THF-d_8. Residual C_4D_7HO and self-correlation peaks are denoted as * and ~ respectively.

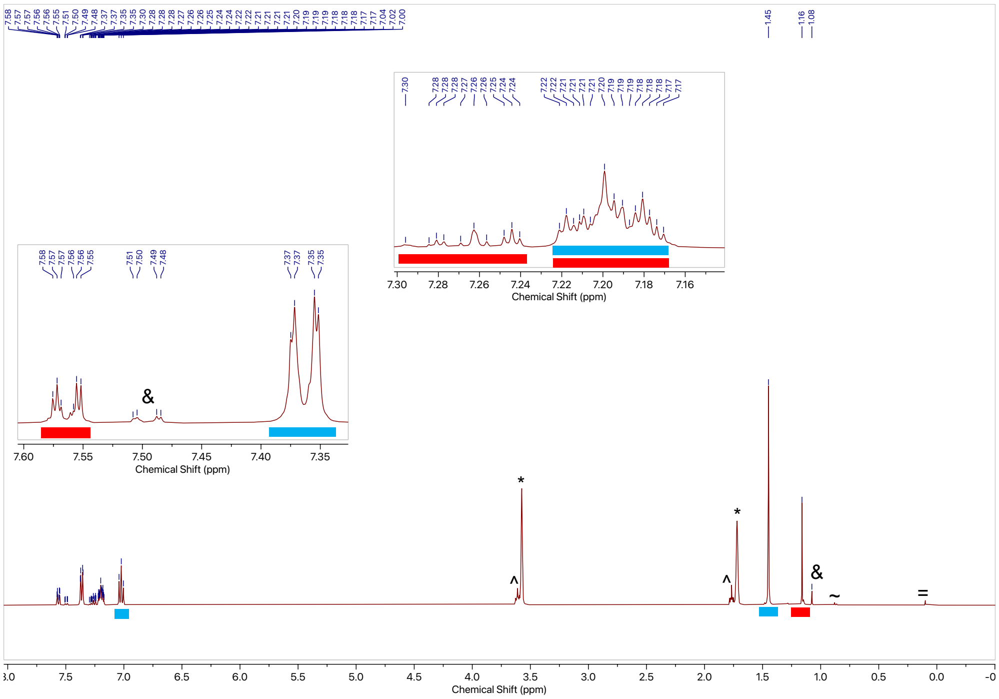

Figure S37. ^{1}H NMR spectrum of the crude product of reaction of **2-Yb** and $AgBArF_{20}$ in THF-d_8. Residual C_4D_7HO, protio-THF, pentane, and Si grease are denoted as *, ^, ~, and = respectively. Features correlated with **2-Yb** and **1-H** are denoted with blue and red colored boxes under the features respectively. Note, the group of features between 7.17-7.22 ppm contains overlapped features from both species. Diamagnetic impurities likely associated with ligand decomposition are denoted as &.

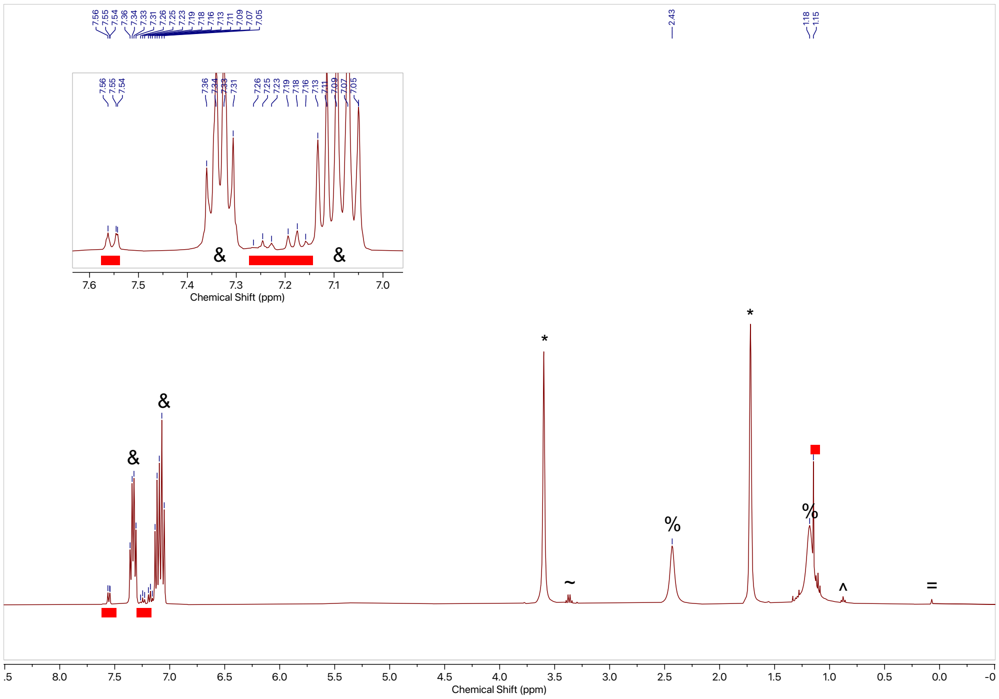

Figure S38. ^{1}H NMR spectrum of the crude product of reaction of **2-Yb** and $FcBArF_{20}$ in THF-d_8. Residual C_4D_7HO, pentane, Et_2O, Si grease, and PhF are denoted as *, ^, ~, =, and & respectively. Features clearly associated with **1-H** are denoted with red colored boxes. Broad, unassigned features are denoted as %.

IR Spectra

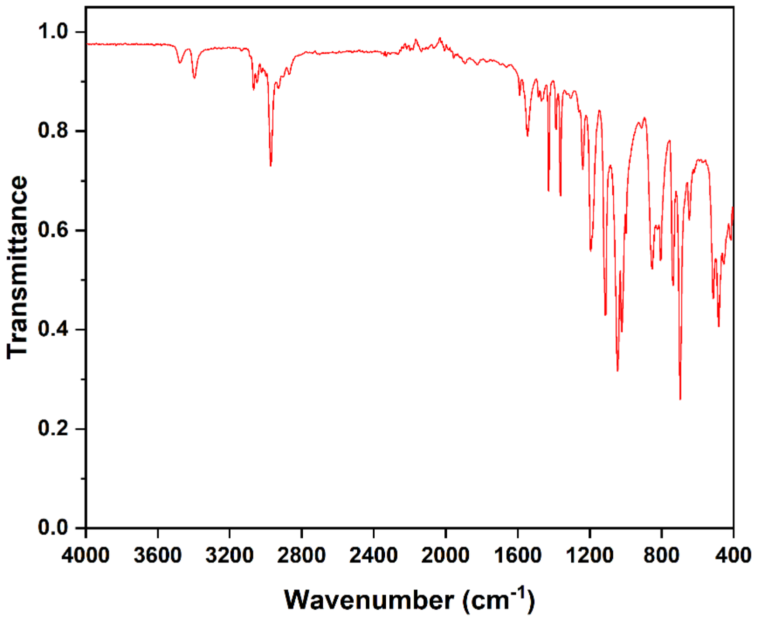

Figure S39. IR spectrum of $Ph_2SiO^tBuNH_2$.

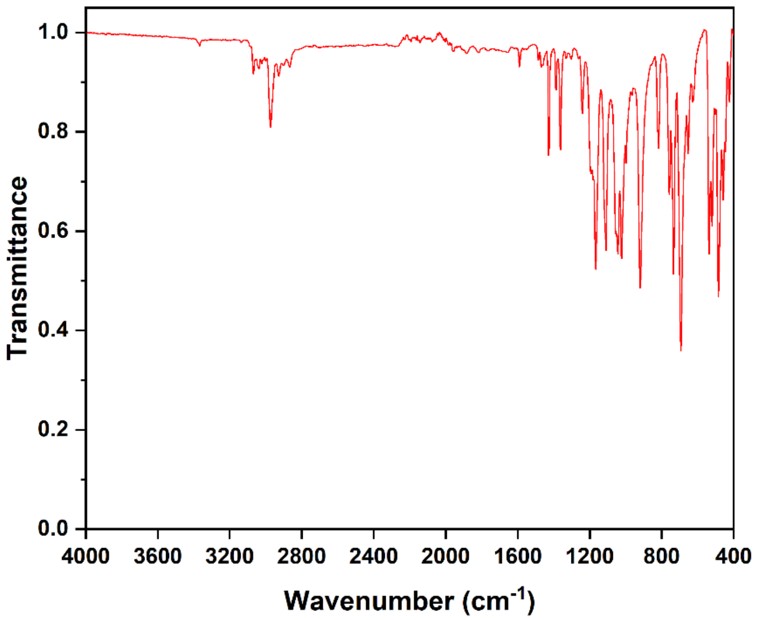

Figure S40. IR spectrum of **1-H**.

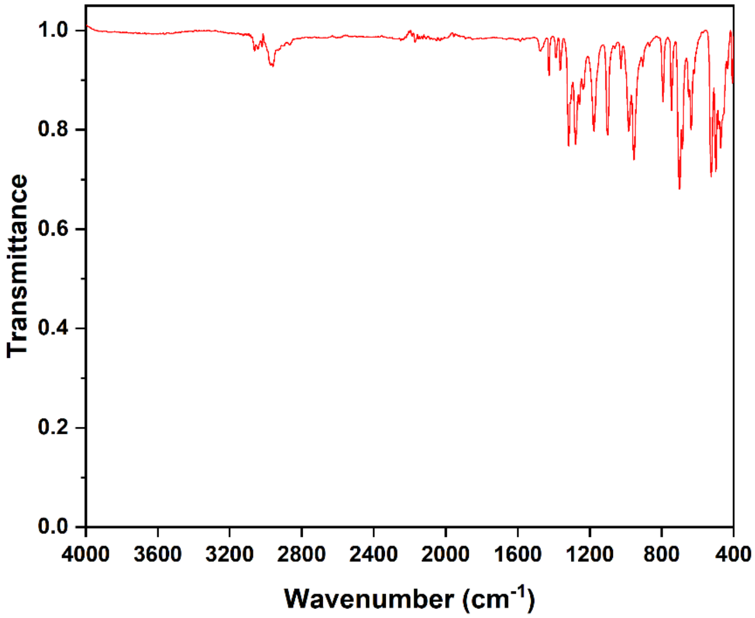

Figure S41. IR spectrum of **1-K**.

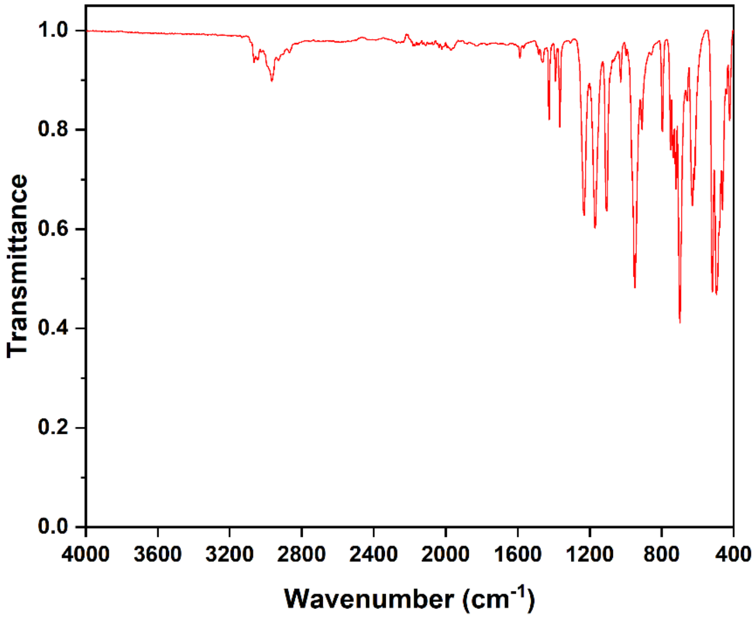

Figure S42. IR Spectrum of **2-Sm**.

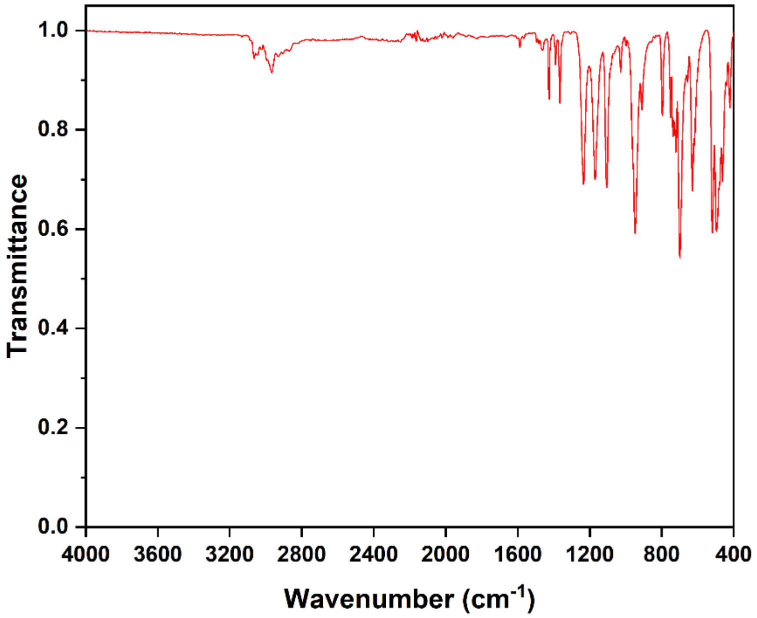

Figure S43. IR spectrum of **2-Eu**.

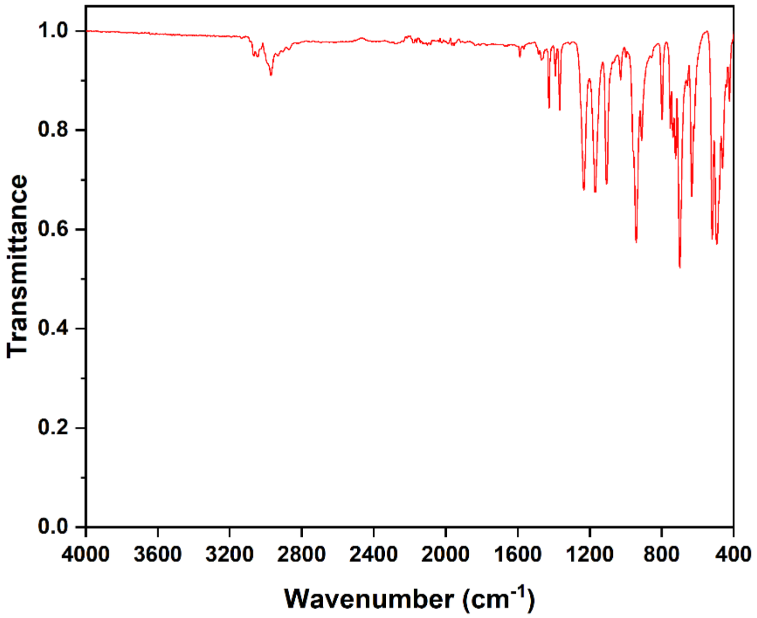

Figure S44. IR spectrum of **2-Yb**.

Electronic Absorption Spectroscopy

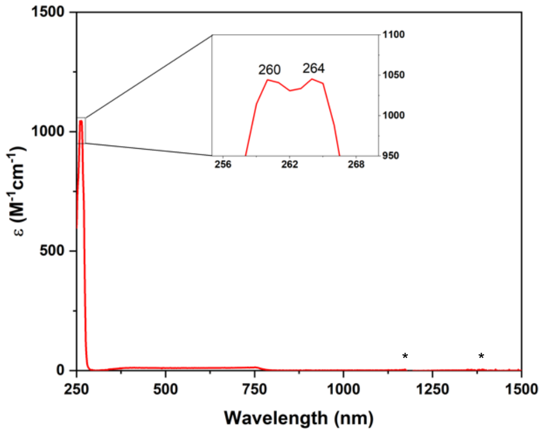

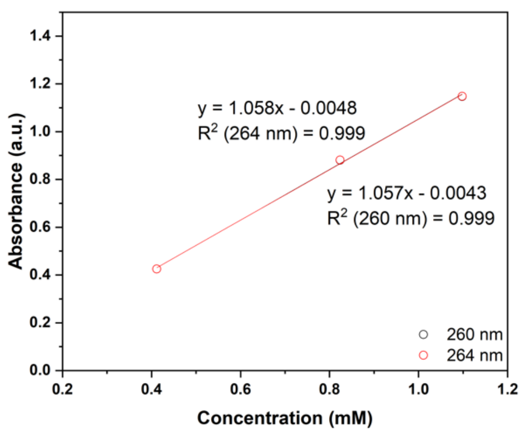

Figure S45. (Left) UV-Vis-NIR spectrum of **1-H** (1.098 mM in THF, * denotes solvent/instrument artifacts) and (right) linear regressions of absorbance for peaks centered at 260 nm and 264 nm at concentrations of 1.098 mM, 823.5 µM, and 411.8 µM.

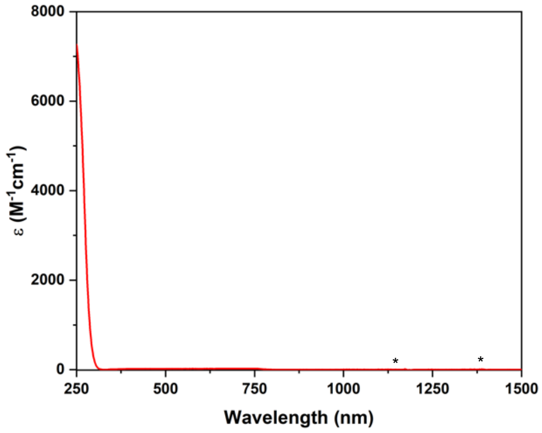

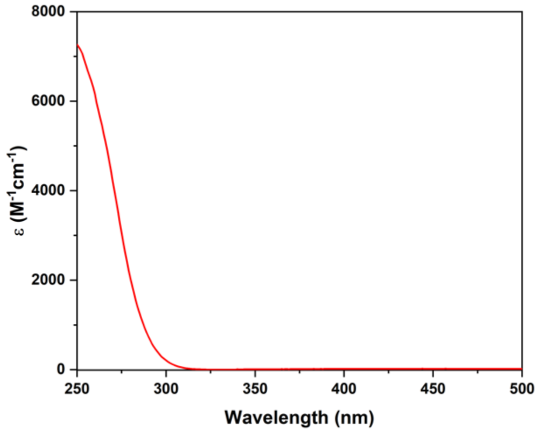

Figure S46. (Left) UV-Vis-NIR spectrum of **1-K** (529.0 µM in THF, * denotes solvent/instrument artifacts) and (right) zoomed portion of UV-Vis-NIR spectrum from 250-500 nm demonstrating no clear absorption peaks in the investigated region(s).

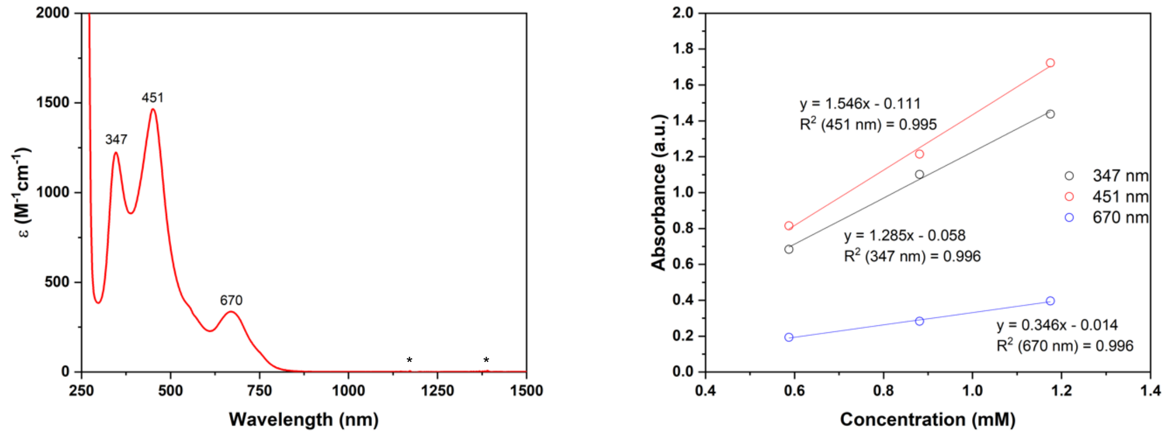

Figure S47. (Left) UV-Vis-NIR spectrum of **2-Sm** (1.175 mM in THF, * denotes solvent/instrument artifacts) and (right) linear regressions of absorbance for peaks centered at 347 nm, 451 nm, and 670 nm at concentrations of 1.175 mM, 881.3 µM, and 587.5 µM.

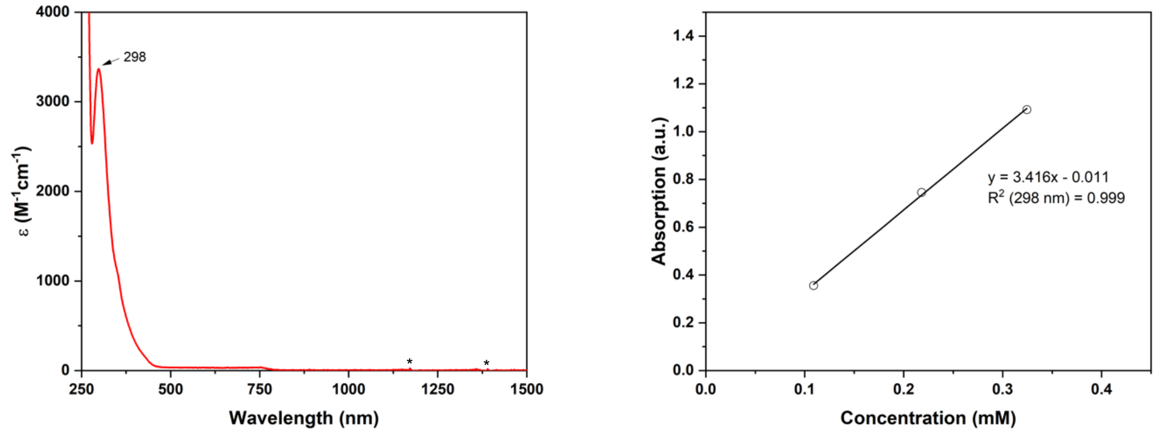

Figure S48. (Left) UV-Vis-NIR spectrum of **2-Eu** (324.5 µM in THF, * denotes solvent/instrument artifacts) and (right) linear regression of absorbance for peak centered at 298 nm at concentrations of 324.5 µM, 218.1 µM, and 109.0 µM.

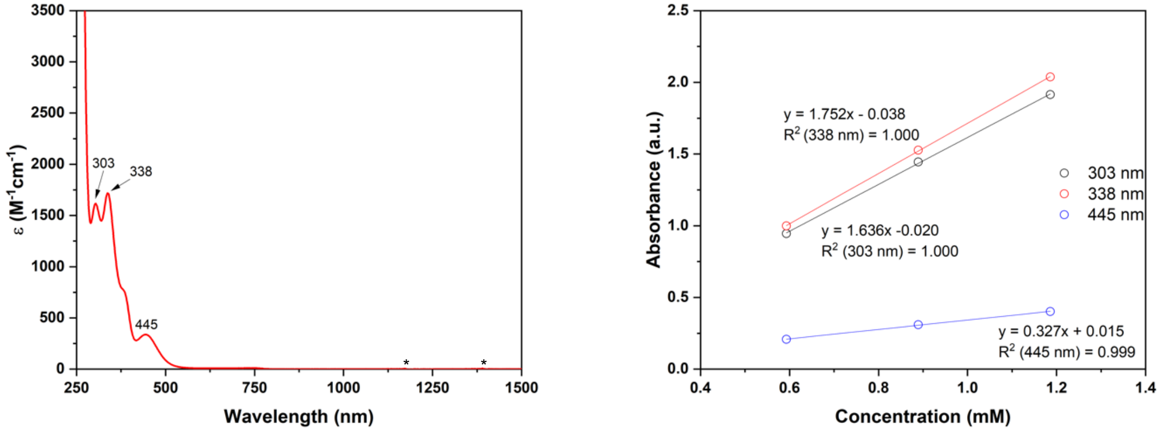

Figure S49. (Left) UV-Vis-NIR spectrum of **2-Yb** (1.186 mM in THF, * denotes solvent/instrument artifacts) and (right) linear regressions of absorbance for peaks centered at 303 nm, 338 nm, and 445 nm at concentrations of 1.186 mM, 889.5 µM, and 593.0 µM.

Cyclic Voltammetry

All electrochemical characterization was performed in an inert-atmosphere glovebox using a Pine WaveDriver 20 potentiostat and a three-electrode cell (WE: glassy carbon; RE: polished Ag wire pseudoreference, fritted; CE: Pt wire). All measurements were recorded at ~25 °C in THF or fluorobenzene (PhF) dried and degassed as outlined in General Considerations. All CVs are plotted according to the IUPAC convention and referenced to the $Fc^{+/0}$ couple via an internal ferrocene and/or decamethyl ferrocene standard. The full electrochemical window of -2.3 V to 1.2 V for 200 mM [N(^{n}Bu)$_4$][PF$_6$] in PhF was examined with no other features unless otherwise noted. Unless otherwise noted graphically or in the caption, the scans shown are sweep segments 3 and 4 (out of 6) ("cycle 2"). The direction of the scan and the starting point of the scan (or segment) is indicated graphically by arrows adjacent to the voltammogram. Applied voltage was corrected for Ohmic drop using positive feedback R_u. E_p values of all redox couples were determined using the first derivative.

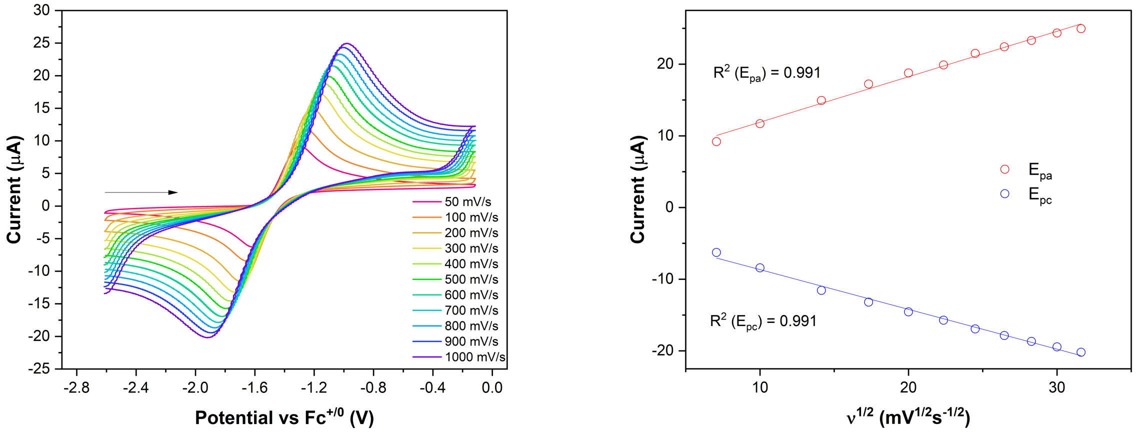

Figure S50. Scan-rate dependence of 1.9 mM **2-Sm** in 200 mM [N(^{n}Bu)$_4$][PF$_6$] in PhF (left) and Randles-Sevcik plot (right).

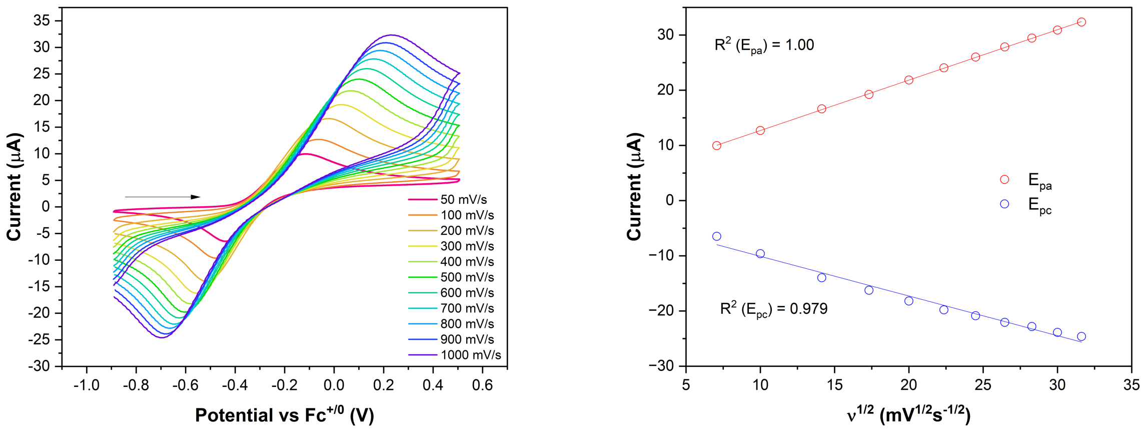

Figure S51. Scan-rate dependence of 2.0 mM **2-Eu** in 200 mM [N(^{n}Bu)$_4$][PF$_6$] in PhF (left) and Randles-Sevcik plot (right).

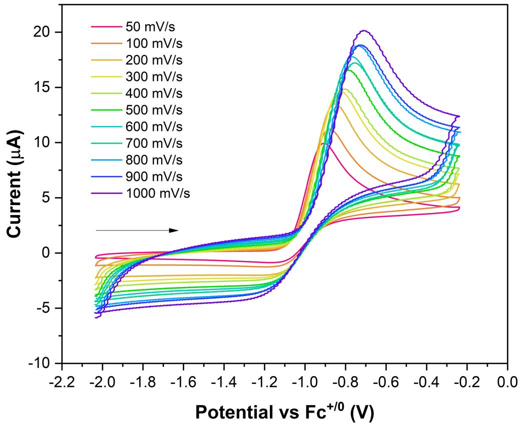

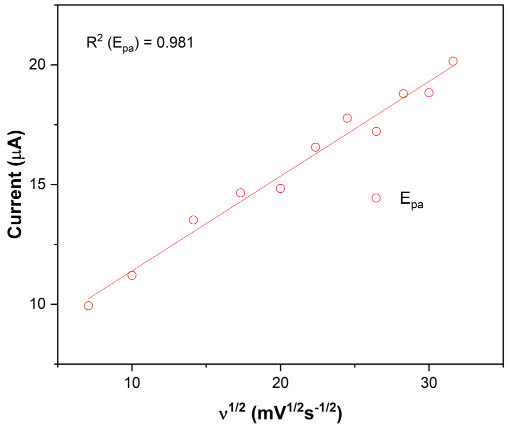

Figure S52. Scan-rate dependence of 2.0 mM **2-Yb** in 200 mM [N(^{n}Bu)$_4$][PF$_6$] in PhF (left) and Randles-Sevcik plot (right).

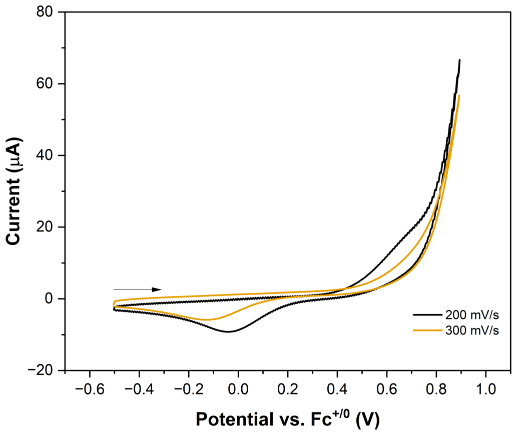

Figure S53. Cyclic voltammograms of 2.1 mM **2-Eu** in 50 mM [N(^{n}Bu)$_4$][BPh$_4$] in THF at 200 mV/s ~5 min after compound was added to electrolyte solution and at 300 mV/s ~10 min after compound was added to electrolyte solution. The disappearance of the E_{pa} peak and drop in magnitude of E_{pc} over a short period of time shows the instability of this compound in this electrolyte and is the reason the [N(^{n}Bu)$_4$][PF$_6$] in PhF was used.

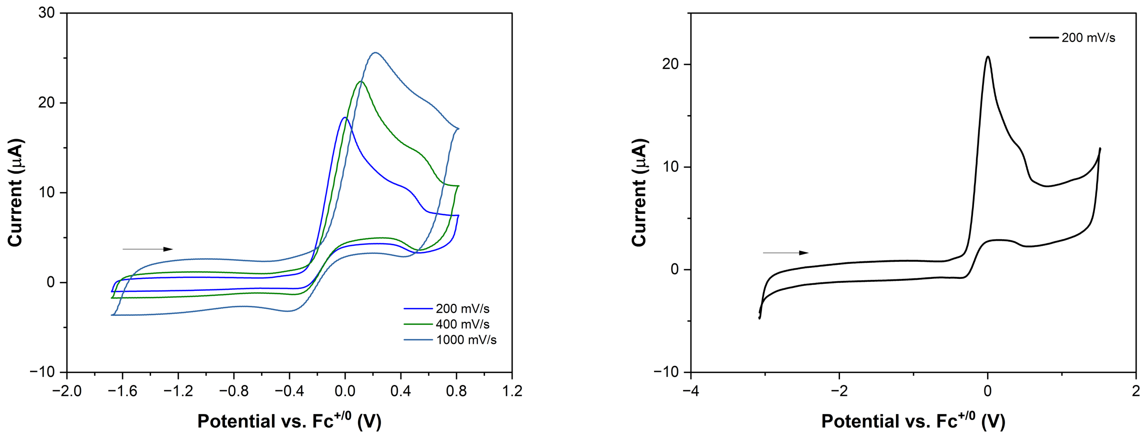

Figure S54. Scan-rate dependence of 2.0 mM **1-K** in 200 mM [N(^{n}Bu)$_4$][PF$_6$] in PhF (left) and full range (right). The E_{pa} peak seen near 0.5 V is the decamethyl ferrocene reference. The compound was not stable in this electrolyte and so only a limited number of scan-rates were gathered prior to significant changes in peak magnitudes and shapes of the scan-rate dependence.

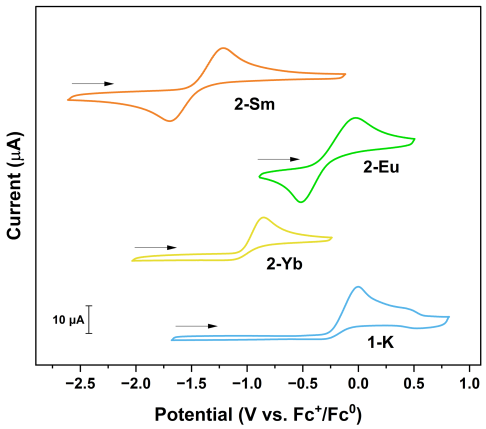

Figure S55. Cyclic voltammograms of 1.9 mM **2-Sm** (top), 2.0 mM **2-Eu** (second), 2.0 mM **2-Yb** (third), and 2.0 mM **1-K** (bottom) at 200 mV/s.

Crystallography

Table S1. Crystallographic data for **1-H** and **1-K** (Methods A and B).

Compound	***1-H***	***1-K** (Method A)*	***1-K** (Method B)*
Empirical formula	$C_{32}H_{39}NO_2Si_2$	$C_{32}H_{38}KNO_2Si_2$	$C_{48}H_{78}KNO_{10}Si_2$
Formula weight	525.82	563.91	924.39
Temperature [K]	100(2)	100(2)	100(2)
Crystal system	Triclinic	Triclinic	Monoclinic
Space group (No.)	$P\bar{1}$ (2)	$P\bar{1}$ (2)	$P2/n$ (10)
a [Å]	8.7119(8)	16.247(3)	14.020(2)
b [Å]	11.2443(10)	18.570(3)	11.6050(15)
c [Å]	15.7371(15)	21.191(3)	17.046(2)
α [°]	93.782(4)	75.166(5)	90
β [°]	102.648(4)	79.661(6)	106.368(5)
γ [°]	94.495(4)	88.257(6)	90
Volume [Å³]	1494.1(2)	6079.0(17)	2661.0(6)
Z	2	8	2
ρ_{calc} [gcm⁻³]	1.169	1.232	1.154
μ [mm⁻¹]	0.147	0.282	0.197
F(000)	564	2400.0	1000.0
Crystal size [mm³]	0.384×0.215×0.169	0.2×0.2×0.2	0.22×0.258×0.276
Crystal color/habit	Colorless blocks	Clear colorless blocks	Clear colorless blocks
Radiation	MoK_α (λ=0.71073 Å)	MoK_α (λ=0.71073 Å)	MoK_α (λ=0.71073 Å)
2θ range [°]	3.65 to 56.90 (0.75 Å)	3.878 to 51.364 (0.82 Å)	4.304 to 54.966 (0.77\` Å)
Index ranges	−11 ≤ h ≤ 11, −15 ≤ k ≤ 15, −21 ≤ l ≤ 21	−19 ≤ h ≤ 19, −22 ≤ k ≤ 22, −25 ≤ l ≤ 25	−18 ≤ h ≤ 18, −14 ≤ k ≤ 15, −21 ≤ l ≤ 21
Reflections collected	64165	188959	41162
Independent reflections	7476 [R_{int} = 0.0825, R_{sigma} = 0.0394]	23088 [R_{int} = 0.1493, R_{sigma} = 0.0802]	6085 [R_{int} = 0.0573, R_{sigma} = 0.0345]
Completeness to θ = 25.242°	100.0 %	99.9%	99.6%
Data / Restraints / Parameters	7476/0/344	23088/18/1393	6085/0/288
Goodness-of-fit on F²	1.033	1.044	1.051
Final R indexes [I≥2σ(I)]	R_1 = 0.0423, wR_2 = 0.1001	R_1 = 0.0754, wR_2 = 0.1604	R_1 = 0.0350, wR_2 = 0.0822
Final R indexes [all data]	R_1 = 0.0534, wR_2 = 0.1077	R_1 = 0.1253, wR_2 = 0.1844	R_1 = 0.0458, wR_2 = 0.0897
Largest peak/hole [eÅ⁻³]	0.44/-0.44	1.438/-0.360	0.351/-0.278
CCDC number	2415057	2411061	2411055

Table S2. Crystallographic data for **2-Ln** (Ln = Sm, Eu, Yb).

Compound	***2-Sm***	***2-Eu***	***2-Yb***
Empirical formula	$C_{64}H_{76}N_2O_4Si_4Sm$, C_7H_8, 0.5(C_5H_{12})	$C_{64}H_{76}N_2O_4Si_4Eu$, 1.5(C_6H_{14})	$C_{64}H_{76}N_2O_4Si_4Yb$, C_7H_8
Formula weight	1328.18	1330.84	1314.8
Temperature [K]	100(2)	100(2)	100(2)
Crystal system	Triclinic	Triclinic	Triclinic
Space group (No.)	$P\bar{1}$(2)	$P\bar{1}$(2)	$P\bar{1}$ (2)
a [Å]	13.675(3)	13.386(2)	13.622(2)
b [Å]	14.844(3)	15.438(2)	15.037(3)
c [Å]	19.461(4)	19.558(3)	19.281(4)
α [°]	91.156(7)	92.004(5)	90.293(7)
β [°]	105.141(8)	104.843(6)	104.734(6)
γ [°]	112.662(7)	113.692(5)	113.121(6)
Volume [Å³]	3486.4(12)	3534.1(9)	3488.3(11)
Z	2	2	2
ρ_{calc} [gcm^{-3}]	1.265	1.251	1.252
μ [mm^{-1}]	0.959	1.002	1.455
F(000)	1390	1400	1364
Crystal size [mm³]	0.068×0.075×0.241	0.131×0.133×0.212	0.192×0.197×0.214
Crystal color/habit	Clear dark yellow plates	Clear emerald green blocks	Clear yellow blocks
Radiation	MoK_α (λ=0.71073 Å)	MoK_α (λ=0.71073 Å)	MoK_α (λ=0.71073 Å)
2θ range [°]	4.684 to 61.016 (0.71 Å)	4.386 to 52.744 (0.80 Å)	4.400 to 56.564 (0.75 Å)
Index ranges	-19 ≤ h ≤ 19, -21 ≤ k ≤ 21, -27 ≤ l ≤ 27	−16 ≤ h ≤ 16, −19 ≤ k ≤ 19, −24 ≤ l ≤ 24	−18 ≤ h ≤ 18, −20 ≤ k ≤ 20, −25 ≤ l ≤ 25
Reflections collected	229982	102607	207284
Independent reflections	21281 [R_{int} = 0.0808, R_{sigma} = 0.0418]	14416 [R_{int} = 0.0839, R_{sigma} = 0.0375]	17292 [R_{int} = 0.0973, R_{sigma} = 0.0421]
Completeness to θ = 25.242°	99.9 %	99.7%	99.9%
Data / Restraints / Parameters	21281/147/894	14416/75/800	17292/0/752
Goodness-of-fit on F²	1.141	1.042	1.066
Final R indexes [I≥2σ(I)]	R_1 = 0.0393, wR_2 = 0.0692	R_1 = 0.0268, wR_2 = 0.0702	R_1 = 0.0330, wR_2 = 0.0749
Final R indexes [all data]	R_1 =0.0587, wR_2 = 0.0779	R_1 = 0.0349, wR_2 = 0.0734	R_1 = 0.0429, wR_2 = 0.0790
Largest peak/hole [eÅ^{-3}]	0.933/−1.153	0.788/−0.655	1.094/−1.193
CCDC number	2411060	2411059	2411058

Table S3. Crystallographic data for **2-Tm** and **3-Tm**.

Compound	***2-Tm***	***3-Tm***
Empirical formula	$C_{69}H_{88}N_2O_4Si_4Tm$	$C_{74.5}H_{103}IN_4O_8Si_4Tm_2$
Formula weight	1290.70	1759.72
Temperature [K]	100(2)	100(2)
Crystal system	Triclinic	Triclinic
Space group (No.)	$P\bar{1}$(2)	$P\bar{1}$ (2)
a [Å]	13.3137(10	11.7413(15)
b [Å]	15.3380(9)	15.7207(19)
c [Å]	19.4002(15)	22.881(3)
α [°]	91.513(3)	95.479(5)
β [°]	104.805(3)	103.192(5)
γ [°]	113.884(2)	106.290(4)
Volume [Å³]	3464.1(4)	3889.0(9)
Z	2	2
ρ_{calc} [gcm^{-3}]	1.237	1.503
μ [mm^{-1}]	1.395	2.779
F(000)	1346.0	1778.0
Crystal size [mm³]	0.106×0.216×0.262	0.104×0.152×0.243
Crystal color/habit	Clear orangish yellow block	Clear yellow prism
Radiation	MoK_α (λ=0.71073 Å)	MoK_α (λ=0.71073 Å)
2θ range [°]	4.416 to 66.184 (0.65 Å)	4.078 to 52.744 (0.80 Å)
Index ranges	-20 ≤ h ≤20, -23 ≤ k ≤ 22, -28 ≤ l ≤ 28	-16 ≤ h ≤ 16, -22 ≤ k ≤ 22, 0 ≤ l ≤ 32
Reflections collected	287149	15894
Independent reflections	25154 [R_{int} = 0.0770, R_{sigma} = 0.0506]	15894 [R_{int} = 0.1678, R_{sigma} = 0.0444]
Completeness to θ = 25.242°	99.9 %	99.9 %
Data / Restraints / Parameters	25154/44/756	15894/41/909
Goodness-of-fit on F²	1.059	1.067
Final R indexes [I≥2σ(I)]	R_1 = 0.0361, wR_2 = 0.0611	R_1 = 0.0527, wR_2 = 0.1267
Final R indexes [all data]	R_1 = 0.0577, wR_2 = 0.0678	R_1 = 0.0696, wR_2 = 0.1356
Largest peak/hole [eÅ^{-3}]	1.170/-0.758	1.937/-1.688
CCDC number	2411056	2411057

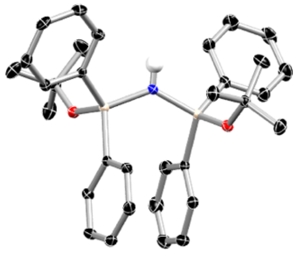

Figure S56. Molecular structure of **1-H**, thermal ellipsoids set at 50 % probability. Black, red, blue, tan, and white represent C, O, N, Si, and H respectively and H atoms (except N-H) are omitted for clarity.

Solution and Refinement Details for 1-H. All data were integrated with SAINT and a multi-scan absorption correction using SADABS was applied.[20, 21] The structure was solved by iterative methods using olex2.solve and refined by full-matrix least-squares methods against F^2 by SHELXL using Olex2.[22, 23] All non-hydrogen atoms were refined with anisotropic displacement parameters. All hydrogen atoms were refined with isotropic displacement parameters. Some were refined freely and some on calculated positions using a riding model with their U_{iso} values constrained to 1.5 times the U_{eq} of their pivot atoms for terminal sp^3 carbon atoms and 1.2 times for all other carbon atoms.

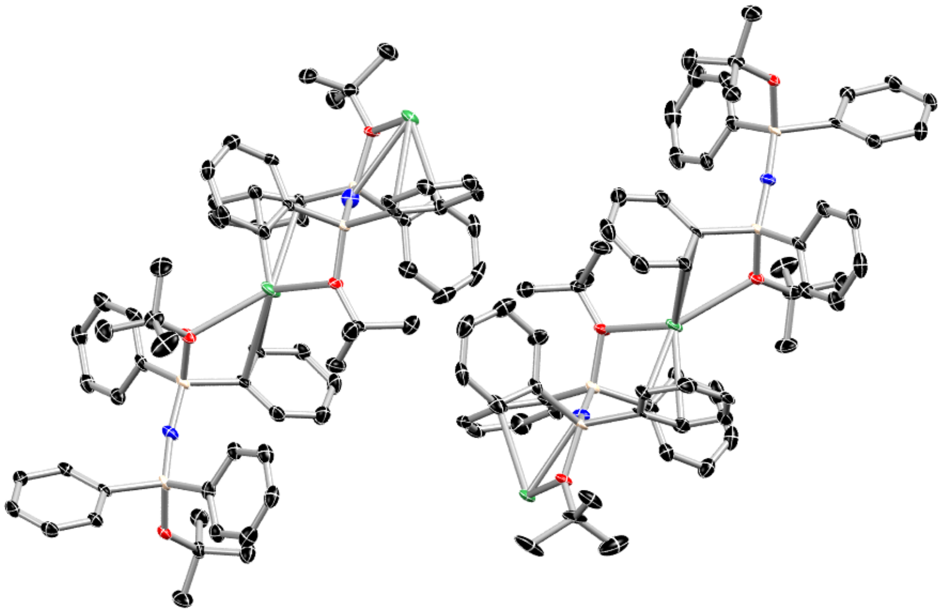

Figure S57. Molecular structure of **1-K** (Method A), thermal ellipsoids set at 50 % probability. Black, red, blue, tan, and green represent C, O, N, Si, and K respectively and H atoms are omitted for clarity.

Solution and Refinement Details for 1-K (Method A). All data were integrated with SAINT and a multi-scan absorption correction using SADABS was applied.[20, 21] The structure was solved by dual methods using SHELXT and refined by full-matrix least-squares methods against F^2 by SHELXL using Olex2.[22-24] All non-hydrogen atoms were refined with anisotropic displacement parameters. All hydrogen atoms were refined isotropically on calculated positions using a riding model with their U_{iso} values constrained to 1.5 times the U_{eq} of their pivot atoms for terminal sp^3 carbon atoms and 1.2 times for all other carbon atoms. Carbons C66-C68 and all attached hydrogens had their thermal parameters retrained with a default RIGU.

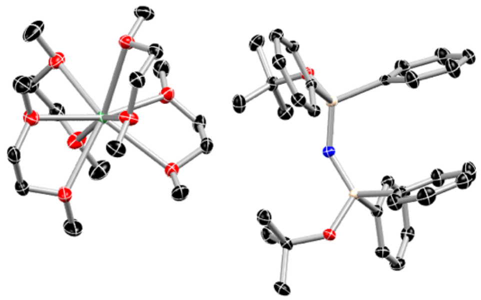

Figure S58. Molecular structure of **1-K** (Method B), thermal ellipsoids set at 50 % probability. Black, red, blue, tan, and green represent C, O, N, Si, and K respectively and H atoms are omitted for clarity.

Solution and Refinement Details for 1-K (Method B). All data were integrated with SAINT and a multi-scan absorption correction using SADABS was applied.[20, 21] The structure was solved by dual methods using SHELXT and refined by full-matrix least-squares methods against F^2 by SHELXL using Olex2.[22-24] All non-hydrogen atoms were refined with anisotropic displacement parameters. All hydrogen atoms were refined isotropically on calculated positions using a riding model with their U_{iso} values constrained to 1.5 times the U_{eq} of their pivot atoms for terminal sp^3 carbon atoms and 1.2 times for all other carbon atoms. No further restraints or disorder modeling were necessary.

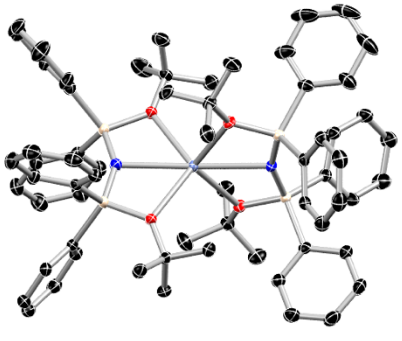

Figure S59. Molecular structure of **2-Sm**, thermal ellipsoids set at 50 % probability. Black, red, blue, tan, and heather-grey represent C, O, N, Si, and Sm respectively. H atoms, minor *tert*-butyl disorder components, and disordered solvent molecules are omitted for clarity.

Solution and Refinement Details for 2-Sm. All data were integrated with SAINT and a multi-scan absorption correction using SADABS was applied.[20, 21] The structure was solved by dual methods using SHELXT and refined by full-matrix least-squares methods against F^2 by SHELXL using Olex2.[22-24] All non-hydrogen atoms were refined with anisotropic displacement parameters. All hydrogen atoms were refined isotropically on calculated positions using a riding model with their U_{iso} values constrained to 1.5 times the U_{eq} of their pivot atoms for terminal sp^3 carbon atoms and 1.2 times for all other carbon atoms. The structure contains disordered solvent electron density consisting of two toluene and one pentane residue within the asymmetric unit that were modelled explicitly. Both toluene residues (C65-C71 and C72-C78) were modeled at a fixed 0.5 occupancy and with all 1,2 and 1,3 C-C distances restrained with DFIX and DANG to 1.4 and 2.5 Å, respectively. The toluene containing C65-C71 lies on a special position, and thus it was modeled in part −1 to prevent erroneous connectivity. The pentane residue (C72A-C76A) was modeled at a fixed 0.5 occupancy and had its 1,2 distances restrained to 1.5 Å with DFIX. All solvent atoms had thermal parameters restrained with RIGU. Finally, C34-C36 are affected by rotational disorder; they were split using the SPLIT SAME command to enforce a SADI restraint between two parts, and their occupancies (summing to 1) and thermal parameters were allowed to refine freely.

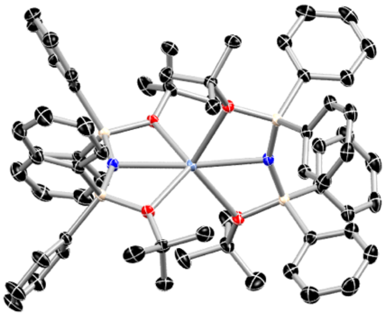

Figure S60. Molecular structure of **2-Eu**, thermal ellipsoids set at 50 % probability. Black, red, blue, tan, and blue-grey represent C, O, N, Si, and Eu respectively. H atoms and disordered solvent molecules are omitted for clarity.

Solution and Refinement Details for 2-Eu. All data were integrated with SAINT and a multi-scan absorption correction using SADABS was applied.[20, 21] The structure was solved by dual methods using SHELXT and refined by full-matrix least-squares methods against F^2 by SHELXL using Olex2.[22-24] All non-hydrogen atoms were refined with anisotropic displacement parameters. All hydrogen atoms were refined isotropically on calculated positions using a riding model with their U_{iso} values constrained to 1.5 times the U_{eq} of their pivot atoms for terminal sp^3 carbon atoms and 1.2 times for all other carbon atoms. The structure contains two disordered hexane moieties in the asymmetric unit that were modelled explicitly. The fragment containing C71-C73 is near a 2-fold rotation axis intersecting the center of the molecule, constructing half of the residue on symmetry generation. All 1,2 and 1,3 distances, including to symmetry-grown atoms, were restrained to 1.5 and 2.5 Å, respectively. The residue containing C65-C70 has positional disorder and is located on a general position away from any symmetry elements. Atoms C65, C66, and C68 can be modeled as full occupancy and present for both conformers of the residue. Atoms C67/C67A, C69/C69A, and C70/C70A are modeled at a fixed 0.5 occupancy. All solvent atoms have had their thermal parameters restrained with RIGU.

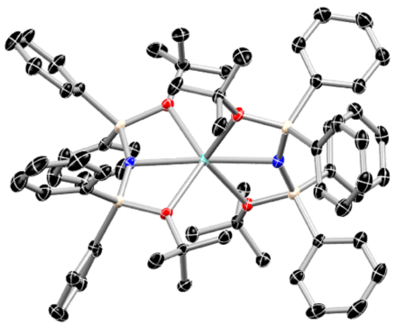

Figure S61. Molecular structure of **2-Yb**, thermal ellipsoids set at 50 % probability. Black, red, blue, tan, and cyan represent C, O, N, Si, and Yb respectively. H atoms and disordered toluene are omitted for clarity.

Solution and Refinement Details for 2-Yb. All data were integrated with SAINT and a multi-scan absorption correction using SADABS was applied.[20, 21] The structure was solved by dual methods using SHELXT and refined by full-matrix least-squares methods against F^2 by SHELXL using Olex2.[22-24] All non-hydrogen atoms were refined with anisotropic displacement parameters. All hydrogen atoms were refined isotropically on calculated positions using a riding model with their U_{iso} values constrained to 1.5 times the U_{eq} of their pivot atoms for terminal sp^3 carbon atoms and 1.2 times for all other carbon atoms. The structure contains an explicitly modeled toluene residue within the asymmetric unit that can be modelled at full occupancy without any additional restraints.

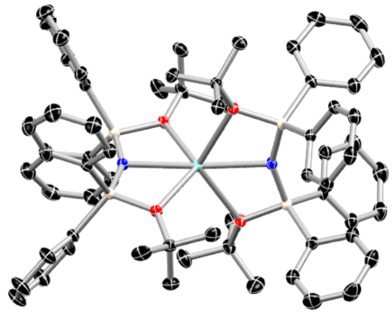

Figure S62. Molecular structure of **2-Tm**, thermal ellipsoids set at 50 % probability. Black, red, blue, tan, and cyan represent C, O, N, Si, and Tm respectively. H atoms and disordered solvent molecules are omitted for clarity.

Solution and Refinement Details for 2-Tm. All data were integrated with SAINT and a multi-scan absorption correction using SADABS was applied.[20, 21] The structure was solved by dual methods using SHELXT and refined by full-matrix least-squares methods against F^2 by SHELXL using Olex2.[22-24] All non-hydrogen atoms were refined with anisotropic displacement parameters. All hydrogen atoms were refined isotropically on calculated positions using a riding model with their U_{iso} values constrained to 1.5 times the U_{eq} of their pivot atoms for terminal sp^3 carbon atoms and 1.2 times for all other carbon atoms. The asymmetric unit contains a pentane residue on a general position with positional disorder on several carbon atoms. Carbons C66 and C69 had their positional disorder modeled with the SPLIT SAME command, which applies a SADI restraint as well, and both carbons had their fractional occupancies tied to the same free variable. All 1,2 and 1,3 C-C distances in the fragment are restrained with DFIX and DANG to 1.4 and 2.5 Å, respectively. The thermal parameters of all carbons in the residue were restrained with a default RIGU. No other restraints were necessary during refinement.

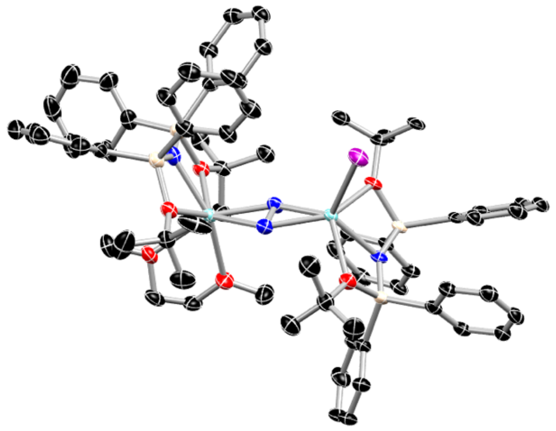

Figure S63. Molecular structure of **3-Tm**, thermal ellipsoids set at 50 % probability. Black, red, blue, tan, purple, and cyan represent C, O, N, Si, I, and Tm respectively. H atoms, minor disordered components on Tm and I sites, and disordered/non-coordinated solvent molecules are omitted for clarity.

Solution and Refinement Details for 3-Tm. All data were integrated with SAINT and a multi-scan absorption correction using TWINABS was applied.[20, 21] The structure was solved by dual methods using SHELXT and refined by full-matrix least-squares methods against F^2 by SHELXL using Olex2.[22-24] All non-hydrogen atoms were refined with anisotropic displacement parameters. All hydrogen atoms were refined isotropically on calculated positions using a riding model with their U_{iso} values constrained to 1.5 times the U_{eq} of their pivot atoms for terminal sp^3 carbon atoms and 1.2 times for all other carbon atoms. The entire structure displays a degree of translational disorder, though this was only able to be modeled in Tm1, Tm2, and I1. The three atoms had their disorder initially modeled with the SPLIT SAME command, setting occupancies of split atoms to sum to 1 and applying a SADI command. The positions and occupancies were then refined isotropically. After the relative occupancies had stabilized, they were fixed, and the atoms were allowed to refine anisotropically with a RIGU restraint. Furthermore, a disordered hexane moiety exists with an inversion special position between the third and fourth carbon. Carbon

atoms C75 and C76 displayed positional disorder and were thus split with the SPLIT command without including SADI. After initial refinement, atoms had their occupancies set to 0.75 in Part 1 and 0.25 in Part 2, and a default RIGU restraint was applied before final anisotropic refinement.

Table S4. Continuous shape measure (CShM) results for **2-Ln**.

Compound	*HP-6*[a]	*PPY-6*[b]	*OC-6*[c]	*TPR-6*[d]	*JPPY-6*[e]
2-Sm	25.499	17.860	18.428	14.529	21.244
2-Eu	25.765	18.366	18.138	14.738	21.796
2-Tm	26.170	18.236	16.487	13.742	21.815
2-Yb	26.700	17.839	16.122	13.323	21.527

[a]HP-6: Hexagon (D_{6h})
[b]PPY-6: Pentagonal pyramid (C_{5v})
[c]OC-6: Octahedron (O_h)
[d]TPR-6: Trigonal prism (D_{3h})
[e]JPPY-6: Johnson pentagonal pyramid J2 (C_{5v})

Table S5. Continuous shape measure (CShM) results for [Ln(BTTSA)$_2$][a].

Compound	*HP-6*[b]	*PPY-6*[c]	*OC-6*[d]	*TPR-6*[e]	*JPPY-6*[f]
[Sm(BTTSA)$_2$]	26.025	19.427	18.306	15.154	22.796
[Eu(BTTSA)$_2$]	26.319	19.466	18.255	14.904	22.690

[a]Major disordered components used when applicable.
[b]HP-6: Hexagon (D_{6h})
[c]PPY-6: Pentagonal pyramid (C_{5v})
[d]OC-6: Octahedron (O_h)
[e]TPR-6: Trigonal prism (D_{3h})
[f]JPPY-6: Johnson pentagonal pyramid J2 (C_{5v})

DC Magnetometry

Magnetic measurements were performed on a Quantum Design MPMS3 magnetometer. Samples were prepared inside a glovebox by adding a measured amount of sample to a shelved quartz tube using a plastic spatula to transfer and a glass pipet as a funnel. A measured amount of quartz wool was then added to the tube and compressed onto the sample using a PTFE rod to fix the sample. The unsealed top of the tube was then attached to a Swagelok UltraTorr adapter, sealed, and taken out of the glovebox. The apparatus was then evacuated on a Schlenk line, and the tube sealed using an H_2/O_2 torch. The sealed tube was pressure-fitted into a plastic straw using PTFE tape and loaded onto the instrument. Diamagnetic corrections were performed for the samples and quartz wool using Pascal's constants (tabulated in Table S6).[25]

Table S6. Molecular weights and diamagnetic corrections for **2-Sm**, **2-Eu**, and quartz wool.

Compound	*Molecular Weight (g/mol)*	*Diamagnetic Correction (10^{-6} emu/mol)*
2-Sm	1200.02	-733.94
2-Eu	1201.63	-732.94
Quartz Wool (SiO_2)	60.08	-2.5

Contributions from the quartz sample tube and plastic straw are negligible compared to the sample and were not considered in this analysis. Variable temperature and field, zero field cooled (ZFC) measurements were measured as previously described.[26] Measurements were performed in duplicate on independently prepared samples of each compound to ensure replicability within error of the measurement.

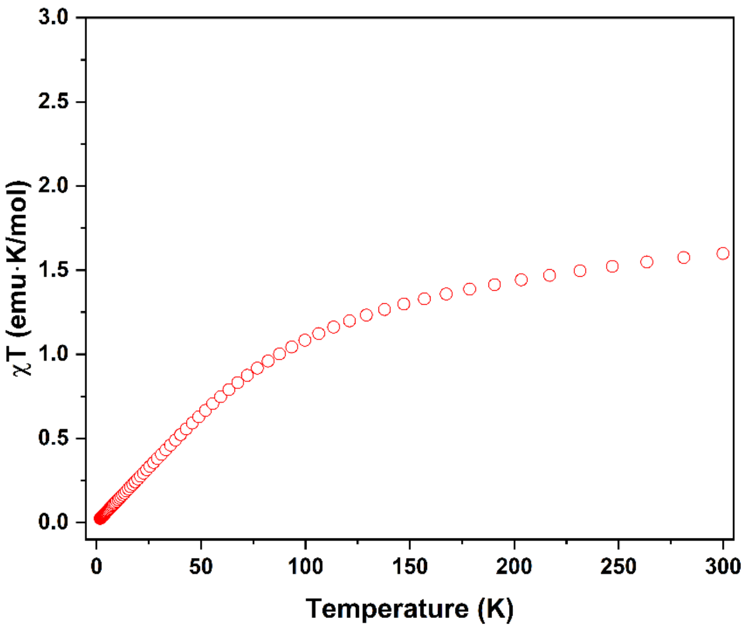

Figure S64. χT vs. T measured at 1.0 T (ZFC) for **2-Sm** from 1.8-300 K.

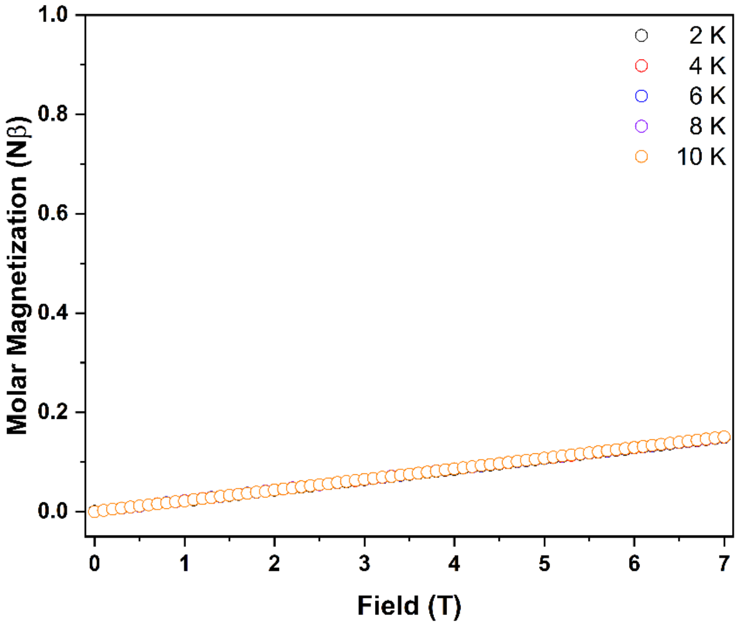

Figure S65. Molar magnetization for **2-Sm** under an applied field of 0-7 T measured at 2, 4, 6, 8, and 10 K (ZFC).

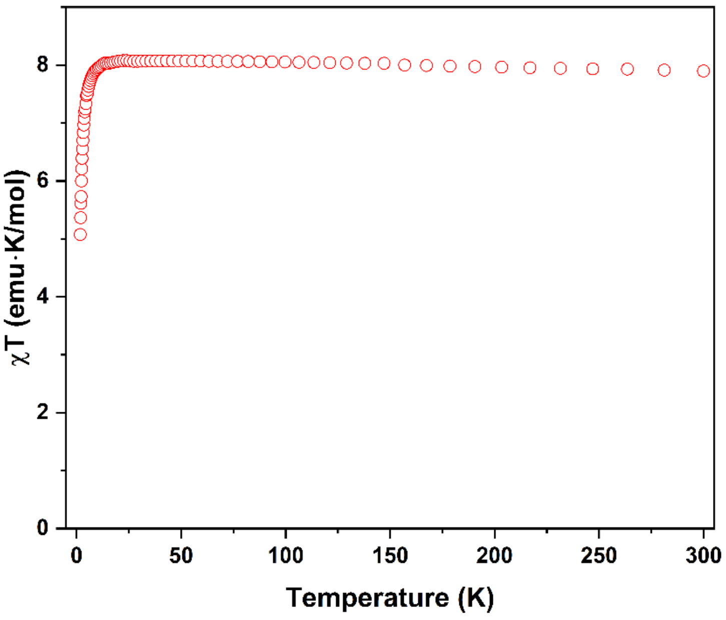

Figure S66. χT vs. T measured at 1.0 T (ZFC) for **2-Eu** from 1.8-300 K.

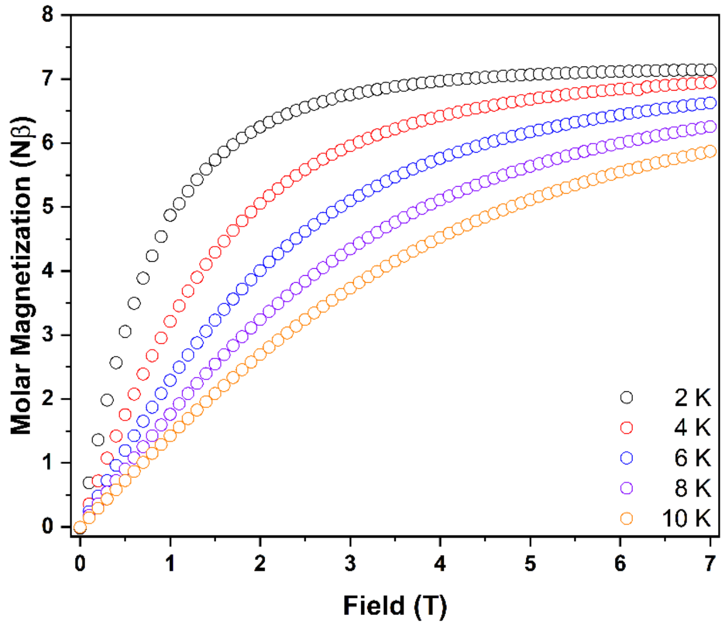

Figure S67. Molar magnetization for **2-Eu** under an applied field of 0-7 T measured at 2, 4, 6, 8, and 10 K (ZFC).

References

(1) Lavinda, O.; Witt, C. H.; Woerpel, K. A. Origin of High Diastereoselectivity in Reactions of Seven-Membered-Ring Enolates. *Angew. Chem. Int. Ed.* **2022**, *61* (14), e202114183. DOI: 10.1002/anie.202114183.

(2) Johnson, S. A.; Kiernicki, J. J.; Fanwick, P. E.; Bart, S. C. New Benzylpotassium Reagents and Their Utility for the Synthesis of Homoleptic Uranium(IV) Benzyl Derivatives. *Organometallics* **2015**, *34* (12), 2889-2895. DOI: 10.1021/acs.organomet.5b00212.

(3) Gillard, J. W.; Fortin, R.; Morton, H. E.; Quesnelle, C. A.; Daignault, S.; Guindon, Y.; Yoakim, C. Symmetrical alkoxysilyl ethers. A new class of alcohol-protecting groups. Preparation of tert-butoxydiphenylsilyl ethers. *J. Org. Chem.* **1988**, *53* (11), 2602-2608. DOI: 10.1021/JO00246A038.

(4) Watson, P. L.; Tulip, T. H.; Williams, I. Defluorination of perfluoroolefins by divalent lanthanoid reagents: activating carbon-fluorine bonds. *Organometallics* **1990**, *9* (7), 1999-2009. DOI: 10.1021/om00157a006.

(5) Gompa, T. P.; Rice, N. T.; Russo, D. R.; Aguirre Quintana, L. M.; Yik, B. J.; Bacsa, J.; La Pierre, H. S. Diethyl ether adducts of trivalent lanthanide iodides. *Dalton Trans.* **2019**, *48* (23), 8030-8033. DOI: 10.1039/c9dt00775j.

(6) Roy, M. D.; Gompa, T. P.; Greer, S. M.; Jiang, N.; Nassar, L. S.; Steiner, A.; Bacsa, J.; Stein, B. W.; La Pierre, H. S. Intervalence Charge Transfer in Nonbonding, Mixed-Valence, Homobimetallic Ytterbium Complexes. *J. Am. Chem. Soc.* **2024**, *146* (8), 5560-5568. DOI: 10.1021/jacs.3c13906.

(7) Hayashi, Y.; Rohde, J. J.; Corey, E. J. A Novel Chiral Super-Lewis Acidic Catalyst for Enantioselective Synthesis. *J. Am. Chem. Soc.* **1996**, *118* (23), 5502-5503. DOI: 10.1021/ja960766s.

(8) Spinney, H. A.; Clough, C. R.; Cummins, C. C. The titanium tris-anilide cation $[Ti(N[^tBu]Ar)_3]^+$ stabilized as its perfluoro-tetra-phenylborate salt: structural characterization and synthesis in connection with redox activity of 4,4′-bipyridine dititanium complexes. *Dalton Trans.* **2015**, *44* (15), 6784-6796. DOI: 10.1039/c5dt00105f.

(9) Harris, R. K.; Becker, E. D.; Cabral de Menezes, S. M.; Goodfellow, R.; Granger, P. NMR nomenclature. Nuclear spin properties and conventions for chemical shifts (IUPAC Recommendations 2001). *Pure Appl. Chem.* **2001**, *73* (11), 1795-1818. DOI: 10.1351/pac200173111795.

(10) Harris, R. K.; Becker, E. D.; Cabral de Menezes, S. M.; Granger, P.; Hoffman, R. E.; Zilm, K. W. Further conventions for NMR shielding and chemical shifts (IUPAC Recommendations 2008). *Pure Appl. Chem.* **2008**, *80* (1), 59-84. DOI: 10.1351/pac200880010059.

(11) Fulmer, G. R.; Miller, A. J. M.; Sherden, N. H.; Gottlieb, H. E.; Nudelman, A.; Stoltz, B. M.; Bercaw, J. E.; Goldberg, K. I. NMR chemical shifts of trace impurities: Common laboratory solvents, organics, and gases in deuterated solvents relevant to the organometallic chemist. *Organometallics* **2010**, *29* (9), 2176-2179. DOI: 10.1021/om100106e.

(12) Groom, C. R.; Bruno, I. J.; Lightfoot, M. P.; Ward, S. C. The Cambridge Structural Database. *Acta Cryst. B* **2016**, *72* (2), 171-179. DOI: 10.1107/s2052520616003954.

(13) *FinalCIF, V109*; https://dkratzert.de/finalcif.html.

(14) *FinalCIF, V113*; https://dkratzert.de/finalcif.html.

(15) *FinalCIF, V125*; https://dkratzert.de/finalcif.html.

(16) Macrae, C. F.; Sovago, I.; Cottrell, S. J.; Galek, P. T. A.; Mccabe, P.; Pidcock, E.; Platings, M.; Shields, G. P.; Stevens, J. S.; Towler, M.; Wood, P. A. *Mercury 4.0*: from visualization to analysis, design and prediction. *J. Appl. Cryst.* **2020**, *53* (1), 226-235. DOI: 10.1107/s1600576719014092.
(17) *POV-Ray, v3.7.0*; http://www.povray.org/.
(18) *SHAPE v2.1*; Electronic Structure Group, Universitat de Barcelona: Universitat de Barcelona, Barcelona, Spain, 2013.
(19) Lukens, W. W.; Minasian, S. G.; Booth, C. H. Strengths of covalent bonds in LnO_2 determined from O K-edge XANES spectra using a Hubbard model. *Chem. Sci.* **2023**, *14* (44), 12784-12795. DOI: 10.1039/d3sc03304j.
(20) *SAINT, v8.40B*; Madison, Wisconsin, USA.
(21) Krause, L.; Herbst-Irmer, R.; Sheldrick, G. M.; Stalke, D. Comparison of silver and molybdenum microfocus X-ray sources for single-crystal structure determination. *J. Appl. Cryst.* **2015**, *48* (1), 3-10. DOI: 10.1107/s1600576714022985.
(22) Dolomanov, O. V.; Bourhis, L. J.; Gildea, R. J.; Howard, J. A. K.; Puschmann, H. OLEX2: a complete structure solution, refinement and analysis program. *J. Appl. Cryst.* **2009**, *42* (2), 339-341. DOI: 10.1107/S0021889808042726.
(23) Sheldrick, G. M. Crystal structure refinement with *SHELXL*. *Acta Cryst. C* **2015**, *71* (1), 3-8. DOI: 10.1107/s2053229614024218.
(24) Sheldrick, G. M. *SHELXT*– Integrated space-group and crystal-structure determination. *Acta Cryst. A* **2015**, *71* (1), 3-8. DOI: 10.1107/s2053273314026370.
(25) Bain, G. A.; Berry, J. F. Diamagnetic Corrections and Pascal's Constants. *J. Chem. Ed.* **2008**, *85* (4), 532. DOI: 10.1021/ed085p532.
(26) Boggiano, A. C.; Chowdhury, S. R.; Roy, M. D.; Bernbeck, M. G.; Greer, S. M.; Vlaisavljevich, B.; La Pierre, H. S. A Four-Coordinate Pr^{4+} Imidophosphorane Complex. *Angew. Chem. Int. Ed.* **2024**, *63* (43), e202409789. DOI: 10.1002/anie.202409789.
